# Regiospecific Hydrogenation of Bromochalcone by Unconventional Yeast Strains

**DOI:** 10.3390/molecules27123681

**Published:** 2022-06-08

**Authors:** Mateusz Łużny, Dagmara Kaczanowska, Barbara Gawdzik, Alicja Wzorek, Aleksandra Pawlak, Bożena Obmińska-Mrukowicz, Monika Dymarska, Ewa Kozłowska, Edyta Kostrzewa-Susłow, Tomasz Janeczko

**Affiliations:** 1 Department of Food Chemistry and Biocatalysis, Wrocław University of Environmental and Life Sciences, Norwida 25, 50-375 Wrocław, Poland; mat.luzny@gmail.com (M.Ł.); kaczanowskad@gmail.com (D.K.); monika.dymarska@upwr.edu.pl (M.D.); ewa.kozlowska1@upwr.edu.pl (E.K.); edyta.kostrzewa-suslow@upwr.edu.pl (E.K.-S.); 2 Institute of Chemistry, Jan Kochanowski University in Kielce, Uniwersytecka 7, 25-406 Kielce, Poland; b.gawdzik@ujk.edu.pl (B.G.); alicja.wzorek@ujk.edu.pl (A.W.); 3 Department of Pharmacology and Toxicology, Wrocław University of Environmental and Life Sciences, C.K. Norwida 31, 50-375 Wrocław, Poland; aleksandra.pawlak@upwr.edu.pl (A.P.); b.mrukowicz@gmail.com (B.O.-M.)

**Keywords:** biotransformations, sweeteners, dihydrochalcones, yeast

## Abstract

This research aimed to select yeast strains capable of the biotransformation of selected 2′-hydroxybromochalcones. Small-scale biotransformations were carried out using four substrates obtained by chemical synthesis (2′-hydroxy-2″-bromochalcone, 2′-hydroxy-3″-bromochalcone, 2′-hydroxy-4″-bromochalcone and 2′-hydroxy-5′-bromochalcone) and eight strains of non-conventional yeasts. Screening allowed for the determination of the substrate specificity of selected microorganisms and the selection of biocatalysts that carried out the hydrogenation of tested compounds in the most effective way. It was found that the position of the bromine atom has a crucial influence on the degree of substrate conversion by the tested yeast strains. As a result of the biotransformation of the 2′-hydroxybromochalcones, the corresponding 2′-hydroxybromodihydrochalcones were obtained. The products obtained belong to the group of compounds with high potential as precursors of sweet substances.

## 1. Introduction

Chalcones are a group of plant-derived compounds belonging to the flavonoid family, synthesised through the phenylpropanoid pathway, and they are biogenetic precursors of all known flavonoids. Structurally, chalcones are composed of two aryl groups (A- and B-rings) linked by an open-chain, three-carbon unit α,β-unsaturated carbonyl system. There are numerous reports in the literature on the transformation of chalcones by bacteria, yeasts, filamentous fungi and plants [1,2,3,4,5,6]. The biotransformation of chalcones often results in similar yields as chemical synthesis, but is performed under significantly milder process conditions [2,7,8].

Depending on the substrate structure and the biocatalyst used, chalcones may give products from the reduction of the double bond, cyclisation, hydroxylation, *O*-demethylation and glycosidation [9,10,11,12,13,14]. The reduction of the carbonyl group present in the dihydrochalcones leading to the formation of the corresponding alcohol is also described [15,16,17]. However, chalcones are mainly converted into dihydrochalcones by most of the tested biocatalysts [18,19,20,21].

**Figure 1:**
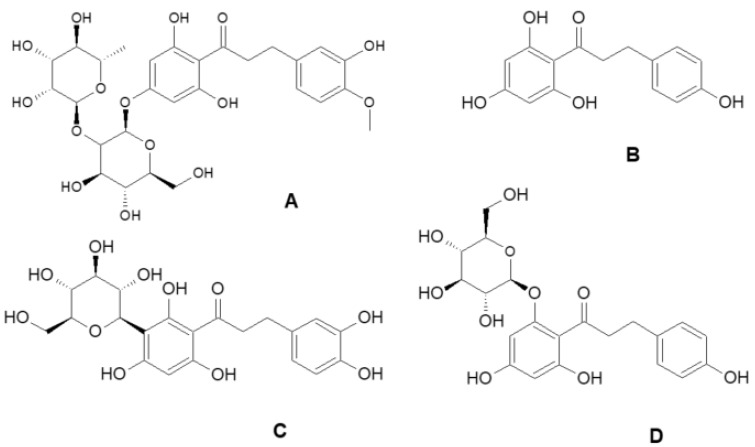
Structure of dihydrochalcones: neohesperidin dihydrochalcone (**A**), phloretin (**B**), aspalathin (**C**) and phloridzin (**D**).

Dihydrochalcones are a class of plant secondary metabolites for which the demand in the food and pharmaceutical industries continues to grow [21]. The best-studied compound of this group, described as an intensely sweet substance, is neohesperidin dihydrochalcone (Figure 1A) [22], which was approved by the European Union to be used as food additive E959 [23]. Moreover, this sweetener (Figure 1A) regulates the expression of genes involved in fatty acid uptake, lipogenesis, adipogenesis, β-oxidation and fat browning in vivo. Thus, neohesperidin dihydrochalcone (Figure 1A) has the promising potential of reducing subcutaneous fat [22].

Two other dihydrochalcones (found in apples, Figure 1)—phloretin (Figure 1B) and phloridzin (Figure 1D)— significantly reduce the risk of the development of cardiovascular diseases and diabetes [24,25,26]. In addition to its antioxidant activity, phloretin (Figure 1B) has been shown to have anti-aging and depigmenting effects [27]. Aspalathin (Figure 1C) (Figure 1), mainly occurring in significant amounts in the leaves of *Aspalathus linearis* (6–13%), reduces oxidative stress and may slow down the ageing process of the organism [28].

In this study, eight unconventional yeast strains—*Rhodotorula rubra* KCh 4 and KCh 82, *Rhodotorula marina* KCh 77, *Rhodotorula glutinis* KCh 242, *Yarrowia lipolytica* KCh 71, *Candida viswanathii* KCh 120, *Saccharomyces cerevisiae* KCh 464 and *Candida parapsilosis* KCh 909—were used to carry out the biotransformation of bromochalcones. These yeast strains were selected because, in our previous studies, we demonstrated their ability to hydrogenate chalcones without forming by products [17,20,29]. We chose the bromine chalcones as substrates because compounds substituted with a bromine atom are highly lipophilic, promoting the compound’s penetration through cell membranes [19]. In addition, the C-Br bond is strongly polarised, and the obtained compounds can be further used in substitution reactions, allowing the replacement of the bromine substituent with another group (hydroxy, methoxy, alkyl and acyl) and thus obtaining substances with the desired properties (i.e., increased solubility or chemical and thermal stability) [30,31].

## 2. Results

**Figure 2:**
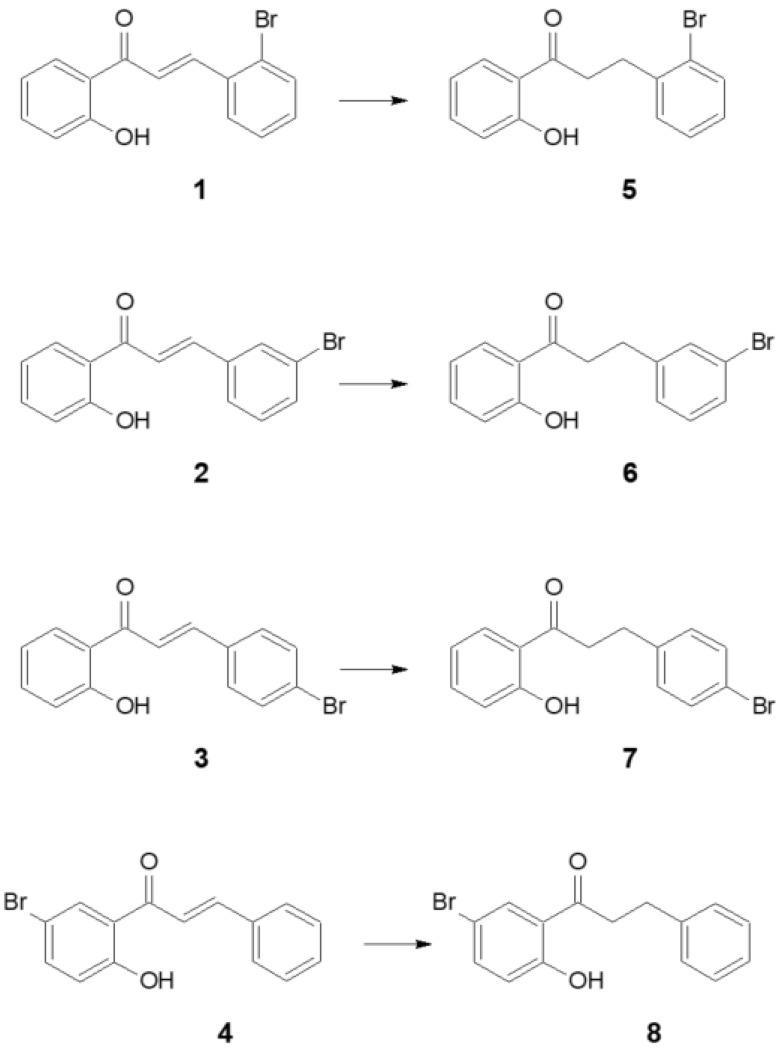
Biotransformation of bromochalcones (**1**–**4**) obtained by chemical synthesis.

The study’s primary purpose was to assess the capacity of various yeast strains for the selective reduction of the double bond in a series of bromochalcones (Figure 2) obtained as a result of chemical synthesis. Additionally, the influence of the position of bromide substituents on the speed of biotransformation was assessed. Eight microorganisms (*Rhodotorula rubra* KCh 4, *Yarrowia lipolytica* KCh 71, *Rhodotorula marina* KCh 77, *Rhodotorula rubra* KCh 82, *Candida viswanathii* KCh 120, *Rhodotorula glutinis* KCh 242, *Saccharomyces cerevisiae* KCh 464 and *Candida parapsilosis* KCh 909) [17,20,29] were chosen based on their previously observed high regioselectivity of hydrogenation during biotransformation, among others such as methoxychalcones and furyl and thienyl analogues of chalcone (3-(2″-furyl)- and 3-(2″-thienyl)-1-(2′-hydroxyphenyl)-prop-2-en-1-one) [20,29].

Based on the obtained results (Table 1), it is possible to determine the preferences for chalcone hydrogenation by selected yeast strains concerning the location of the bromine atom in the structure of the 2′-hydroxychalcone. All strains transformed 2′-hydroxy-2″-bromochalcone (Figure 2) (**1**) and 2′-hydroxy-4″-bromochalcone (**3**) within 10 days with a yield greater than 70%. The lowest degree of conversion (< 10%) was observed for the conversion of 2′-hydroxy-5′-bromochalcone (**4**) by the strains *R. glutinis* KCh 242 and *C. parapsilois* KCh 909 and 2′-hydroxy-3″-bromochalcone (Figure 2) (**2**) by *S. cerevisiae* KCh 464. After 10 days of incubation, it was observed that only three of all tested strains converted substrate **1** with an efficiency of less than 94%: *R. rubra* KCh4 (70%), *R. marina* KCh 77 (61.3%) and *R. rubra* KCh 82 (71.7%). In contrast, the conversion rate of substrate **3** was > 91% in all cases except for the strain *R. glutinis* KCh 242 (87.5%). The five strains converted substrate **2** with a > 90% yield within 10 days. In the *S. cerevisie* KCh 464 strain culture, a product with a conversion rate below 5% was observed, while the conversion rate of the transformation carried out by *R. rubra* KCh 4 and *R. rubra* KCh 82 was 20.8% and 54.3%, respectively. 

The Table 1 and Table 2 show the average conversion values (from three replicates). In none of the measurements did the error in determining the conversion exceed 5%. Based on the results in Table 1, strains that showed the ability to transform the specified substrates with an efficiency of >70% on the first reaction day were selected, and biotransformations were performed by analysing samples after 1, 3, 6 and 12 h of reaction. The analysis of the results (Table 2) shows the ability of all selected strains to transform substrate **1** efficiently. The highest conversion was observed in the culture of the strain *Y. lipolytica* KCh 71 (conversion = 96% after 1 h). Also, substrate **2** was most efficiently transformed by this strain. In the first hour, the reaction yield was 91% and the conversion in the cultures of *R. glutinis* KCh 242 and *C. parapsilosis* KCh 909 did not exceed 30%. After 1 h of incubation, only the *S. cerevisiae* KCh 464 strain showed a moderate substrate conversion capacity of 3–58%, while the reaction yield was 23% in the *C. vaswanathii* KCh 120 culture and only 10% in the *Y. lipolytica* KCh 71 culture. After 12 h of transformation, no significant increase in the amount of product was observed in the *Y. lipolytica* KCh 71 culture (42%) compared to the other two cultures. Compound **4** having a bromine substituent in ring A, unlike the other substrates, was the fastest transformed by the strain *Y. lipolytica* KCh 71. In the culture of this strain, after 12 h of reaction, the degree of conversion of substrates **1**, **2** and **4** was > 97%. On the other hand, substrate **3** was much less accepted by ene-reductases of this strain and the reaction efficiency was significantly different from the other transformations (42%). However, after a longer incubation time, the content of the substrate **3** transformation product in the reaction mixture increased, which confirms the ability of this strain to hydrogenate compound **3** (Figure 2). Based on the above observations, the *Y. lipolytica* KCh 71 strain was selected to perform the biotransformation of all tested compounds on a preparative scale.

One product was obtained as a result of the biotransformation of 2′-hydroxy-2″-bromochalcone (**1**). The analysis of ^1^H NMR and ^13^C NMR spectra (Table 3 and Table 4) allowed us to determine the structure of the obtained product, which was 2′-hydroxy-2″-bromodihydrochalcone (Figure 2). Products **5**–**8** were obtained analogously (Table 4, Table 5, Table 6 and Table 7).

## 3. Discussion

Biotransformations are an alternative to the commonly used chemical syntheses [32]. Due to their high chemoselectivity, enantioselectivity and regioselectivity, biocatalysts are a valuable tool for synthesising chiral products [21]. These compounds can be excellent structural elements for synthesising substances that conventional methods cannot obtain [19]. For this reason, the scientific community is undertaking the identification of new biocatalysts and research on the ability of already known microorganisms to biotransform an ever-increasing group of compounds.

The main aim of the research was to select strains that, by hydrogenating the double bond, most effectively convert selected chalcones to dihydrochalcones. It is essential to synthesise these compounds because when using chemical synthesis to reduce the α,β- double bond, it is necessary to use metals (i.e., palladium or nickel and hydrogen), which may also reduce the carbonyl group [11]. For this reason, biotechnological methods seem to be the most advantageous for obtaining this group of compounds. The available literature provides a great deal of information on the microbial synthesis of dihydrochalcones. The ability to hydrogenate flavonoid compounds was confirmed for many strains of bacteria, yeasts and filamentous fungi [4,11,33,34]. As a result of using *Corynebacterium equi* IFO 3730 bacterial cells, which showed the ability to hydrogenate the double bond selectively, no additional products were detected. The hydrogenation reaction was also effective for substrates having substituents on aromatic rings [1]. Another study involved transforming a series of chalcones with three industrial *Saccharomyces cerevisiae* strains. After optimising the reaction conditions, the degree of substrate conversion was > 99% and the obtained results indicated no correlation between the type of substituents and the transformation efficiency [2]. The use of yeast strains for the hydrogenation of chalcones usually leads to a single product—dihydrochalcone [21,35]. If the biocatalysts are strains of filamentous fungi, it is also possible to obtain other products [9,36]. The dihydrochalcones obtained due to biotransformations are vital products in synthesising flavours, anthocyanins and homoisoflavonoids. They exhibit various pharmacological properties and are often the building blocks of sweeteners [19,21].

During the experiment, transformations of chalcones with bromine in their structure were carried out. This substituent was present at four positions: C-2″, C-3″, C-4″ and C-5″ (Figure 2). In addition, all the substrates had a hydroxyl group in ring A located on the C-2 carbon. The hydroxyl group present on this carbon is a characteristic feature of most natural dihydrochalcones [19,27,34].

The scientific literature shows that microorganisms are less effective in converting 2′-hydroxychalcones than compounds not substituted with a hydroxyl group [16,17,37]. This is probably due to the formation of hydrogen bonds between the electron pair present on the carbonyl oxygen and the hydrogen of the hydroxyl group [19]. It is suspected that this type of steric hindrance makes the substrate less acceptable to the active centres of the enzymes catalysing the biotransformation process [11]. Therefore, it is crucial to search for biocatalysts capable of reducing chalcones containing a hydroxyl group situated at the C-2′ carbon.

Each of the biocatalysts we used showed specificity toward specific substrates, which is particularly noticeable when comparing the course of biotransformations carried out by the same strain. These results indicate the importance of the location of the bromine substituent in the alignment of the compounds to the enzyme’s active sites. The available literature provides information on the preferences of enzymes capable of hydrogenating the chalcone double bond over the location of the halogen substituents. Research indicates better acceptance of substrates with a bromine atom in the para vs. the meta position [19]. Screening tests and GC analysis confirmed the above dependence and showed high efficiency of the transformation of substrates also substituted in the ortho position.

Based on the results of the screening tests, the *Yarrowia lipolytica* KCh 71 strain was selected for biotransformation on the preparative scale. The following amounts of isolated products were obtained from 100 mg of the substrates used: 2′-hydroxy-2″-bromodichydrochalcone—70.4 mg, 2′-hydroxy-3″-bromodihydrochalcone—27.4 mg, 2′-hydroxy-4″-bromodihydrochalcone—5.3 mg and 2′-hydroxy-5′-bromodihydrochalcone—45 mg. The chemical structures of the biotransformation products were determined based on nuclear magnetic resonance (^1^H NMR, ^13^C NMR, COSY, HMBC, HMQC). The comparison of the spectra of the products with the spectra of the substrates allowed for the determination of changes in the chemical shifts of protons present in the three-carbon fragment of chalcone. In all cases, it was confirmed that 2′-hydroxydihydrobromochalcones were obtained, as evidenced by the presence of two multiplets at approx. 3 ppm, derived from protons at carbons C-2 and C-3.

The selection of microorganisms capable of hydrogenating the double bond of 2′-hydroxychalcones, substituted with a bromine atom, is essential due to the properties of the selected substituent. Bromine, belonging to the halogens, is a strong electron-withdrawing group with high reactivity. The C-Br bond is strongly polarised and the consequence of this fact is the increased susceptibility of such a bond to breaking [30,31]. Thanks to this property, the obtained compounds can be used in substitution reactions, allowing the replacement of the bromine substituent with another group and thus obtaining substances with the desired properties (i.e., increased solubility or chemical and thermal stability). In addition, the bromine atom is characterised by high lipophilicity, which promotes the penetration of the compound through the cell membrane [19].

## 4. Materials and Methods

### 4.1. Substrates

The substrates used for biotransformation were obtained by Claisen−Schmidt condensation reaction bromoderivatives of 2-hydroxyacetophenone and benzaldehyde [purchased from Sigma−Aldrich (St. Louis, MO, USA)] dissolved in methanol in an alkaline environment (Table 1) at a high temperature, according to the procedure described previously [38,39,40].

### 4.2. Microorganisms

The research was carried out on eight strains of yeast from the species *Rhodotorula rubra* (KCh 4 and KCh 82), *Rhodotorula marina* KCh 77, *Rhodotorula glutinis* KCh 242, *Yarrowia lipolytica* KCh 71, *Candida viswanathii* KCh 120, *Saccharomyces cerevisiae* KCh 464 and *Candida parapsilosis* KCh 909, whose storage and biocatalytic capacity have been previously described [17,20,29,41].

### 4.3. Screening

Three hundred mL Erlenmeyer flasks were used for biotransformation on an analytical scale, each containing 100 mL of Sabouraud culture medium (3% glucose, 1% aminobac). Transplanted microorganisms were incubated for three days at 24 °C on a rotary shaker (144 rpm). After this time, 10 mg of the substrate was dissolved in DMSO (dimethyl sulfoxide) and added. Samples were collected after 1, 3 and 7 days. Based on the results shown in Table 1, strains that showed the ability to transform the specified substrates with an efficiency of > 70% on the first reaction day were selected, and biotransformations were also performed by analysing samples after 1, 3, 6 and 12 h of reaction. The experiment was carried out in triplicate. Portions of 10 mL of the transformation mixture were extracted with ethyl acetate. The extracts were dried over MgSO_4_, concentrated in vacuo and analysed by gas chromatography (GC) and thin-layer chromatography (TLC).

### 4.4. Gas Chromatography

GC analysis was performed using an Agilent 7890A gas chromatograph, equipped with a flame ionisation detector (FID) (Agilent, Santa Clara, CA, USA). The capillary column DB-5HT (30 m × 0.25 mm × 0.10 µm) was used to determine the composition of the product mixtures. The temperature programme was applied as follows: 80–300 °C, the temperature on the detector: 300 °C, injection 1 µL, flow 1 mL/min, flow H2: 35 mL/min, airflow; 300 mL/min, time of analysis: 18.67 min. The retention times of the substrates and products are described in Table 8.

### 4.5. Preparative Scale

Preparative biotransformations were performed in 2 L Erlenmeyer flasks, each containing 500 mL of culture medium (3% glucose, 1% peptone). The transferred microorganisms were incubated for three days at 24 °C on a rotary shaker. After this time, 100 mg of the substrate dissolved in 2 mL of DMSO were added. After three days, the product was isolated by triple extraction with ethyl acetate (3 extractions with 300 mL), dried with anhydrous magnesium sulfate and concentrated in vacuo. The transformation products were separated by preparative TLC and analysed (TLC, GC, NMR).

### 4.6. TLC and NMR Analysis

The course of biotransformations was monitored using TLC plates (SiO_2_, DC Alufolien Kiesel gel 60 F254 (0.2 mm thick), Merck, Darmstadt, Germany). Products were separated using preparative TLC plates (Silica Gel GF, 20 × 20 cm, 500 μm, Analtech) and a cyclohexane: ethyl acetate mixture (9:1, *v*/*v*) as an eluent, according to a method described previously [29,42]. The product was observed (without additional visualisation) under the UV lamp at a wavelength of 254 nm.

NMR analysis was performed using a DRX 600 MHz Bruker spectrometer (Bruker, Billerica, MA, USA). The prepared samples were dissolved in deuterated chloroform CDCl_3_. The performed analyses include ^1^H NMR, ^13^C NMR, HMBC (two-dimensional analysis), HMQC (heteronuclear correlation) and COSY (correlation spectroscopy) (Supplementary Materials).

## 5. Conclusions

The conducted research allowed for the selection of strains capable of transforming 2′-hydroxybromochalcones.

The presence of a bromine substituent in the ortho, meta and para positions of the A and B rings of chalcones influences the degree of substrate conversion by specific yeast strains.

The enzyme apparatus of the *Yarrowia lipolytica* KCh 71 strain is capable of the biotransformation of all tested substrates; however, the process is catalysed at different rates.

Confirmation of the ability of selected strains to synthesise dihydrochalcones substituted with a bromine atom is of great importance due to the features of the selected substituent and thus, the vast possibilities for the development of new compounds with the desired properties.

## Figures and Tables

**Table 1:** Percentage of biotransformation products of 2′-hydroxybromochalcones on the 1st, 3rd, 7th and 10th day of the reaction.

Strain	Substrate	Degree of Conversion after a Specified Incubation Time			
		1 Day	3 Days	7 Days	10 Days
*Rhodotorula rubra* KCh 4	**1**	7	22	71	75
	**2**	10	13	14	21
	**3**	34	83	99	>99
	**4**	67	87	90	92
*Rhodotorula rubra* KCh 82	**1**	16	24	72	82
	**2**	10	12	21	54
	**3**	34	68	99	>99
	**4**	66	87	88	91
*Rhodotorula marina* KCh 77	**1**	3	11	63	72
	**2**	53	72	90	97
	**3**	23	47	92	99
	**4**	57	81	86	90
*Rhodotorula glutinis* KCh 242	**1**	59	93	97	98
	**2**	93	95	98	99
	**3**	15	38	67	89
	**4**	4	9	11	15
*Saccharomyces cerevisiae* KCh 464	**1**	77	82	98	99
	**2**	2	3	3	4
	**3**	>99	>99	>99	>99
	**4**	63	77	84	92
*Candida viswanathii* KCh 120	**1**	94	96	98	99
	**2**	32	52	94	96
	**3**	98	99	>99	>99
	**4**	6	30	34	75
*Candida parapsilosis* KCh 909	**1**	92	95	97	98
	**2**	80	85	94	95
	**3**	46	92	>99	>99
	**4**	3	15	18	20
*Yarrowia lipolytica* KCh 71	**1**	98	99	>99	>99
	**2**	90	95	97	98
	**3**	74	95	98	92
	**4**	91	93	95	96

**Table 2:** Percentage of biotransformation products of 2′-hydroxybromochalcones in the 1st, 3rd, 6th and 12th hours of the reaction.

Strain	Substrate	Degree of Conversion after Specified Incubation Time			
		1 h	3 h	6 h	12 h
*Rhodotorula rubra* KCh 4	**4**	31	38	45	50
*Rhodotorula glutinis* KCh 242	**2**	21	25	28	31
*Saccharomyces cerevisiae* KCh 464	**1**	31	40	56	71
	**3**	58	81	94	99
	**4**	26	44	57	78
*Candida viswanathii* KCh 120	**1**	81	99	>99	>99
	**3**	23	40	68	94
*Candida parapsilosis* KCh 909	**1**	75	92	94	96
	**2**	29	58	82	88
*Yarrowia lipolytica* KCh 71	**1**	96	98	98	99
	**2**	91	95	96	97
	**3**	10	18	28	42
	**4**	80	93	96	97

**Table 3:** Summary of signals visible in the ^1^H NMR spectrum of the substrate (**1**) and the biotransformation product (**5**).

Proton	Compound	
	1	5
H-2	8.26 (d, 1H, *J* = 15.4 Hz)	3.30–3.36 (m, 2 H)
H-3	7.59 (d, 1 H, *J* = 15.5 Hz)	3.14–3.20 (m, 2 H)
H-3′	7.04 (dd, 1 H, *J* = 8.4, 0.9 Hz)	6.98 (dd, 1 H, *J* = 8.4, 1.2, 0.4 Hz)
H-4′	7.52 (ddd, 1 H, *J* = 8.2, 7.2, 1.4 Hz)	7.45 (ddd, 1 H, *J* = 8.4, 7.1, 1.6 Hz)
H-5′	6.95 (ddd, 1 H, *J* = 8.1, 7.1, 1.0 Hz)	6.87 (ddd, 1 H, *J* = 8.2, 7.1, 1.1 Hz)
H-6′	7.91 (dd, 1 H, *J* = 8.1, 1.4 Hz)	7.76 (dd, 1 H, *J* = 8.1, 1.6 Hz)
H-3″	7.75 (dd, 1 H, *J* = 7.8, 1.4 Hz)	7.55 (dd, 1 H, *J* = 8.0, 1.2 Hz)
H-4″	7.38 (t, 1 H, *J* = 7.5 Hz)	7.24 (td, 1 H, *J* = 7.4, 1.3 Hz)
H-5″	7.28 (td, 1 H, *J* = 7.9, 1.6 Hz)	7.09 (ddd, 1 H, *J* = 7.9, 7.3, 1.9 Hz)
H-6″	7.66 (dd, 1 H, *J* = 8.0, 1.0 Hz)	7.30 (dd, 1 H, *J* = 8.0, 1.2 Hz)
-OH	12.71 (s, 1 H)	12.28 (s, 1 H)

**Table 4:** Summary of signals visible in the ^13^C NMR spectrum of the substrates and the biotransformation products.

Carbon	Compound							
	1	5	2	6	3	7	4	8
C-1	193.59	205.21	193.49	204.86	193.59	205.03	192.87	205.69
C-2	143.86	38.36	143.70	39.75	144.12	39.82	139.10	40.23
C-3	123.14	30.82	121.60	29.57	120.82	29.44	146.72	29.81
C-1′	120.03	119.37	120.02	119.30	120.05	119.34	121.39	120.60
C-2′	163.79	162.55	163,79	162.56	163.77	162.59	162.64	161.42
C-3′	118.85	118.65	118.85	118.74	118.84	118.77	128.80	120.71
C-4′	136.75	136.52	136.80	136.60	136.71	136.62	119.55	139.08
C-5′	119.06	119.08	119.10	119.12	119.05	119.12	110.59	110.61
C-6′	129.89	130.01	129.82	129.85	129.75	129.86	131.98	132.19
C-1″	134.90	140.11	136.84	143.21	133.64	139.83	134.44	140.50
C-2″	126.28	124.46	131.09	127.27	132.45	131.47	129.01	128.52
C-3″	128.15	133.07	123.31	122.72	130.10	130.34	129.25	128.78
C-4″	127.90	127.83	127.63	129.58	125.40	120.24	131.43	126.56
C-5″	131.80	128.30	130.86	130.28	140.10	130.34	129.25	128.78
C-6″	133.83	130.88	133.74	131.61	132.45	131.47	129.01	128.52

**Table 5:** Summary of signals visible in the ^1^H NMR spectrum of the substrate (**2**) and the biotransformation product (**6**).

Proton	Compound	
	2	6
H-2	7.79–7.85 (m, 1 H)	3.29–3.35 (m, 2 H)
H-3	7.64 (d, 1 H, *J* = 15.5 Hz)	3.00–3.08 (m, 2 H)
H-3′	7.04 (d, 1 H, *J* = 8.1 Hz)	6.99 (ddd, 1 H, *J* = 8.4, 1.1, 0.4 Hz)
H-4′	7.52 (t, 1 H, *J* = 7.1)	7.48 (ddd,1 H, *J* = 8.4, 7.2, 1.7 Hz)
H-5′	6.96 (t, 1 H, *J* = 7.2 Hz)	6.89 (dd, 1 H, *J* = 8.2, 7.1, 1.1. Hz)
H-6′	7.92 (d, 1 H, *J* = 7.1 Hz)	7.74 (dd, 1 H, *J* = 8.1, 1.6 Hz)
H-2″	7.79–7.85 (m, 1 H)	7.14–7.20 (m, 1 H)
H-4″	7.54–7.58 (m, 1 H)	7.33–7.37 (m, 1 H)
H-5″	7.04 (d, 1 H, *J* = 8.1 Hz)	7.14–7.20 (m, 1 H)
H-6″	7.54–7.58 (m, 1 H)	7.41–7.42 (m, 1 H)
-OH	12.70 (s, 1 H)	12.23 (s, 1 H)

**Table 6:** Summary of signals visible in the ^1^H NMR spectrum of the substrate (**3**) and the biotransformation product (**7**).

Proton	Compound	
	3	7
H-2	7.84 (d, 1 H, *J* = 15.5 Hz)	3.28–3.34 (m, 2 H)
H-3	7.64 (d, 1 H, *J* = 5.5 Hz)	3.00–3.06 (m, 2 H)
H-3′	7.03 (dd, 1 H, *J* = 8.3, 0.8 Hz)	6.99 (dd, 1 H, *J* = 8.3, 1.1 Hz)
H-4′	7.49–7.53 (m, 1 H)	7.47 (dddd, 1 H, *J* = 8.6, 7.0,1.7, 0.4 Hz)
H-5′	6.95 (ddd, 1 H, *J* = 8.1, 7.1, 1.0 Hz)	6.87 (ddd, 1 H, *J* = 8.2, 7.1, 1.1 Hz)
H-6′	7.90 (dd, 1 H, *J* = 8.1, 1.4 Hz)	7.73 (dd, 1 H, *J* = 8.1, 1.5 Hz)
H-2″	7.56–7.59 (m, 1 H)	7.40–7.44 (m, 1 H)
H-3″	7.49–7.53 (m, 1 H)	7.11–7.15 (m, 1 H)
H-5″	7.49–7.53 (m, 1 H)	7.11–7.15 (m, 1 H)
H-6″	7.56–7.59 (m, 1 H)	7.40–7.44 (m, 1 H)
-OH	12.74 (s, 1 H)	12.23 (s, 1 H)

**Table 7:** Summary of signals visible in the ^1^H NMR spectrum of the substrate (**4**) and the biotransformation product (**8**).

Proton	Compound	
	4	8
H-2	7.56 (d, 1 H, *J* = 15.5 Hz)	3.26–3.32 (m, 2 H)
H-3	7.95 (d, 1 H, *J* = 15.4 Hz)	3.02–3.09 (m, 2 H)
H-3′	6.94 (d, 1 H, *J* = 8.9 Hz)	6.88 (d, 1 H, *J* = 8.9 Hz)
H-4′	7.57 (dd, 1 H, *J* = 8.0, 2.3 Hz)	7.52 (dd, 1 H, *J* = 8.9,2.4 Hz)
H-6′	8.01 (d, 1 H, *J* = 2.3 Hz)	7.82 (d, 1 H, *J* = 2.4 Hz)
H-2″	7.67–7.70 (m, 1 H)	7.20–7.26 (m, 1 H)
H-3″	7.43–7.48 (m, 1 H)	7.29–7.34 (m, 1 H)
H-4″	7.43–7.48 (m, 1 H)	7.20–7.26 (m, 1 H)
H-5″	7.43–7.48 (m, 1 H)	7.29–7.34 (m, 1 H)
H-6″	7.67–7.70 (m, 1 H)	7.20–7.26 (m, 1 H)
-OH	12.74 (s, 1 H)	12.19 (s, 1 H)

**Table 8:** Retention times of substrates and products based on GC.

Retention Times of Substrates (1–4) and Products (5–8) [min]							
1	5	2	6	3	7	4	8
12.26	11.27	12.42	11.53	12.54	11.65	12.09	11.06

## Data Availability

Not applicable.

## Supplementary Materials

The following are available online at https://www.mdpi.com/article/10.3390/molecules27123681/s1, Figure S1. 1H NMR spectral of 1-(2′-hydroxyphenyl)-3-(2″-bromophenyl)-prop-2-en-1-one (**1**) (CDCl3, 600 MHz); Figure S2. Part of the 13C NMR spectral of 1-(2′-hydroxyphenyl)-3-(2″-bromophenyl)-prop-2-en-1-one (**1**) (CDCl3, 600 MHz); Figure S3. 13C NMR spectral of 1-(2′-hydroxyphenyl)-3-(2″-bromophenyl)-prop-2-en-1-one (**1**) (CDCl3, 151 MHz); Figure S4. COSY spectral of 1-(2′-hydroxyphenyl)-3-(2″-bromophenyl)-prop-2-en-1-one (**1**) (CDCl3, 151 MHz); Figure S5. HSQC spectral of 1-(2′-hydroxyphenyl)-3-(2″-bromophenyl)-prop-2-en-1-one (**1**) (CDCl3, 151 MHz); Figure S6. 1H NMR spectral of 1-(2′-hydroxyphenyl)-3-(2″-bromophenyl)-propan-1-one (**5**) (CDCl3, 600 MHz); Figure S7. Part of the 13C NMR spectral of 1-(2′-hydroxyphenyl)-3-(2″-bromophenyl)-propan-1-one (**5**) (CDCl3, 600 MHz); Figure S8. 13C NMR spectral of 1-(2′-hydroxyphenyl)-3-(2″-bromophenyl)-propan-1-one (**5**) (CDCl3, 151 MHz); Figure S9. Part of the 13C NMR spectral of 1-(2′-hydroxyphenyl)-3-(2″-bromophenyl)-propan-1-one (**5**) (CDCl3, 151 MHz); Figure S10. COSY spectral of 1-(2′-hydroxyphenyl)-3-(2″-bromophenyl)-propan-1-one (**5**) (CDCl3, 151 MHz); Figure S11. Part of the COSY spectral of 1-(2′-hydroxyphenyl)-3-(2″-bromophenyl)-propan-1-one (**5**) (CDCl3, 151 MHz); Figure S12. HSQC spectral of 1-(2′-hydroxyphenyl)-3-(2″-bromophenyl)-propan-1-one (**5**) (CDCl3, 151 MHz); Figure S13. HMBC spectral of 1-(2′-hydroxyphenyl)-3-(2″-bromophenyl)-propan-1-one (**5**) (CDCl3, 151 MHz); Figure S14. 1H NMR spectral of 1-(2′-hydroxyphenyl)-3-(3″-bromophenyl)-prop-2-en-1-one (**2**) (CDCl3, 600 MHz); Figure S15. Part of the 13C NMR spectral of 1-(2′-hydroxyphenyl)-3-(3″-bromophenyl)-prop-2-en-1-one (**2**) (CDCl3, 600 MHz); Figure S16. 13C NMR spectral of 1-(2′-hydroxyphenyl)-3-(3″-bromophenyl)-prop-2-en-1-one (**2**) (CDCl3, 151 MHz); Figure S17. COSY spectral of 1-(2′-hydroxyphenyl)-3-(3″-bromophenyl)-prop-2-en-1-one (**2**) (CDCl3, 151 MHz); Figure S18. HSQC spectral of 1-(2′-hydroxyphenyl)-3-(3″-bromophenyl)-prop-2-en-1-one (**2**) (CDCl3, 151 MHz); Figure S19. 1H NMR spectral of 1-(2′-hydroxyphenyl)-3-(3″-bromophenyl)-propan-1-one (**6**) (CDCl3, 600 MHz); Figure S20. Part of the 13C NMR spectral of 1-(2′-hydroxyphenyl)-3-(3″-bromophenyl)-propan-1-one (**6**) (CDCl3, 600 MHz); Figure S21. 13C NMR spectral of 1-(2′-hydroxyphenyl)-3-(3″-bromophenyl)-propan-1-one (**6**) (CDCl3, 151 MHz); Figure S22. COSY spectral of 1-(2′-hydroxyphenyl)-3-(3″-bromophenyl)-propan-1-one (**6**) (CDCl3, 151 MHz); Figure S23. HSQC spectral of 1-(2′-hydroxyphenyl)-3-(3″-bromophenyl)-propan-1-one (**6**) (CDCl3, 151 MHz); Figure S24. HMBC spectral of 1-(2′-hydroxyphenyl)-3-(3″-bromophenyl)-propan-1-one (**6**) (CDCl3, 151 MHz); Figure S25. 1H NMR spectral of 1-(2′-hydroxyphenyl)-3-(4″-bromophenyl)-prop-2-en-1-one (**3**) (CDCl3, 600 MHz); Figure S26. Part of the 1H NMR spectral of 1-(2′-hydroxyphenyl)-3-(4″-bromophenyl)-prop-2-en-1-one (**3**) (CDCl3, 600 MHz); Figure S27. 13C NMR spectral of 1-(2′-hydroxyphenyl)-3-(4″-bromophenyl)-prop-2-en-1-one (**3**) (CDCl3, 151 MHz); Figure S28. COSY spectral of 1-(2′-hydroxyphenyl)-3-(4″-bromophenyl)-prop-2-en-1-one (**3**) (CDCl3, 151 MHz); Figure S29. HSQC spectral of 1-(2′-hydroxyphenyl)-3-(4″-bromophenyl)-prop-2-en-1-one (**3**) (CDCl3, 151 MHz); Figure S30. 1H NMR spectral of 1-(2′-hydroxyphenyl)-3-(4″-bromophenyl)-propan-1-one (**7**) (CDCl3, 600 MHz); Figure S31. Part of the 13C NMR spectral of 1-(2′-hydroxyphenyl)-3-(4″-bromophenyl)-propan-1-one (**7**) (CDCl3, 600 MHz); Figure S32. 13C NMR spectral of 1-(2′-hydroxyphenyl)-3-(4″-bromophenyl)-propan-1-one (**7**) (CDCl3, 151 MHz); Figure S33. COSY spectral of 1-(2′-hydroxyphenyl)-3-(4″-bromophenyl)-propan-1-one (**7**) (CDCl3, 151 MHz); Figure S34. HSQC spectral of 1-(2′-hydroxyphenyl)-3-(4″-bromophenyl)-propan-1-one (**7**) (CDCl3, 151 MHz); Figure S35. HMBC spectral of 1-(2′-hydroxyphenyl)-3-(4″-bromophenyl)-propan-1-one (**7**) (CDCl3, 151 MHz); Figure S36. 1H NMR spectral of 1-(5′-bromo-2′-hydroxyphenyl)-3-phenyl-propan-1-one (**4**) (CDCl3, 600 MHz); Figure S37. Part of the 1H NMR spectral of 1-(5′-bromo-2′-hydroxyphenyl)-3-phenyl-propan-1-one (**4**) (CDCl3, 600 MHz); Figure S38. 13C NMR spectral of 1-(5′-bromo-2′-hydroxyphenyl)-3-phenyl-propan-1-one (**4**) (CDCl3, 151 MHz); Figure S39. COSY spectral of 1-(5′-bromo-2′-hydroxyphenyl)-3-phenyl-propan-1-one (**4**) (CDCl3, 151 MHz); Figure S40. HSQC spectral of 1-(5′-bromo-2′-hydroxyphenyl)-3-phenyl-propan-1-one (**4**) (CDCl3, 151 MHz); Figure S41. 1H NMR spectral of 1-(5′-bromo-2′-hydroxyphenyl)-3-phenyl-prop-2-en-1-one (**8**) (CDCl3, 600 MHz); Figure S42. Part of the 13C NMR spectral of 1-(5′-bromo-2′-hydroxyphenyl)-3-phenyl-prop-2-en-1-one (**8**) (CDCl3, 600 MHz); Figure S43. 13C NMR spectral of 1-(5′-bromo-2′-hydroxyphenyl)-3-phenyl-prop-2-en-1-one (**8**) (CDCl3, 151 MHz); Figure S44. COSY spectral of 1-(5′-bromo-2′-hydroxyphenyl)-3-phenyl-prop-2-en-1-one (**8**) (CDCl3, 151 MHz); Figure S45. HSQC spectral of 1-(5′-bromo-2′-hydroxyphenyl)-3-phenyl-prop-2-en-1-one (**8**) (CDCl3, 151 MHz); Figure S46. HMBC spectral of 1-(5′-bromo-2′-hydroxyphenyl)-3-phenyl-prop-2-en-1-one (**8**) (CDCl3, 151 MHz); Figure S47. Preparative TLC plate showing the separation of the post-reaction mixture after biotransformation of 2′-hydroxy-2″-bromochalcone (**1**); Figure S48. Preparative TLC plate showing the separation of the post-reaction mixture after biotransformation of 2′-hydroxy-3″-bromochalcone (**2**); Figure S49. Preparative TLC plate showing the separation of the post-reaction mixture after biotransformation of 2′-hydroxy-4″-bromochalcone (**3**); Figure S50. Preparative TLC plate showing the separation; of the post-reaction mixture after biotransformation of 2′-hydroxy-5′-bromochalcone (**4**); Figure S51. Selected chromatograms showing the composition of the reaction mixtures during the biotransformation of the tested compounds.
