# Supplementary material for: Regiospecific Hydrogenation of Bromochalcone by Unconventional Yeast Strains
Source: Molecules. 2022 Jun 8;27(12):3681. doi: 10.3390/molecules27123681 (PMC9228445; doi:10.3390/molecules27123681)
Supplement: Supplementary file 1 [file molecules-27-03681-s001.zip › molecules-1748877-supplementary.pdf]

Supplementary material

Regiospecific hydrogenation of bromochalcone by unconventional yeast strains

Mateusz Łużny[1], Dagmara Kaczanowska[1], Barbara Gawdzik[2], Alicja Wzorek[2], Aleksandra Pawlak [3], Bożena Obmińska-Mrukowicz [3], Monika Dymarska[1], Ewa Kozłowska[1], Edyta Kostrzewa-Susłow[1] and Tomasz Janeczko[1,*]

[1] Department of Food Chemistry and Biocatalysis, Wrocław University of Environmental and Life Sciences, Norwida 25, 50-375 Wrocław, Poland

[2] Institute of Chemistry, Jan Kochanowski University in Kielce, Uniwersytecka 7, 25-406 Kielce, Poland;

[3] Department of Pharmacology and Toxicology, Wrocław University of Environmental and Life Sciences, C.K. Norwida 31, 50-375 Wrocław, Poland

* Correspondence: janeczko13@interia.pl (T.J.); Tel.: +48-713-205-195 (T.J.)

e.a.kozlowska@gmail.com (E.K.), kaczanowskad@gmail.com (D.K.), b.gawdzik@ujk.edu.pl (B.G.), alicja.wzorek@ujk.edu.pl (A.W.), aleksandra.pawlak@upwr.edu.pl (A.P.), beatriz.hernandez-suarez@upwr.edu.pl (B.H.S.), monika.dymarska@upwr.edu.pl (M.D.), ewa.kozlowska1@upwr.edu.pl (E.K.), edyta.kostrzewa-suslow@upwr.edu.pl (E.K-S.)

Contents:

Figure S1. ^{1}H NMR spectral of 1-(2′-hydroxyphenyl)-3-(2″-bromophenyl)-prop-2-en-1-one (**1**) ($CDCl_3$, 600 MHz)

Figure S2. Part of the ^{13}C NMR spectral of 1-(2′-hydroxyphenyl)-3-(2″-bromophenyl)-prop-2-en-1-one (**1**) ($CDCl_3$, 600 MHz)

Figure S3. ^{13}C NMR spectral of 1-(2′-hydroxyphenyl)-3-(2″-bromophenyl)-prop-2-en-1-one (**1**) ($CDCl_3$, 151 MHz)

Figure S4. COSY spectral of 1-(2′-hydroxyphenyl)-3-(2″-bromophenyl)-prop-2-en-1-one (**1**) ($CDCl_3$, 151 MHz)

Figure S5. HSQC spectral of 1-(2′-hydroxyphenyl)-3-(2″-bromophenyl)-prop-2-en-1-one (**1**) ($CDCl_3$, 151 MHz)

Figure S6. ^{1}H NMR spectral of 1-(2′-hydroxyphenyl)-3-(2″-bromophenyl)-propan-1-one (**5**) ($CDCl_3$, 600 MHz)

Figure S7. Part of the ^{13}C NMR spectral of 1-(2′-hydroxyphenyl)-3-(2″-bromophenyl)-propan-1-one (**5**) ($CDCl_3$, 600 MHz)

Figure S8. ^{13}C NMR spectral of 1-(2′-hydroxyphenyl)-3-(2″-bromophenyl)-propan-1-one (**5**) ($CDCl_3$, 151 MHz)

Figure S9. Part of the ^{13}C NMR spectral of 1-(2′-hydroxyphenyl)-3-(2″-bromophenyl)-propan-1-one (**5**) ($CDCl_3$, 151 MHz)

Figure S10. COSY spectral of 1-(2′-hydroxyphenyl)-3-(2″-bromophenyl)-propan-1-one (**5**) ($CDCl_3$, 151 MHz)

Figure S11. Part of the COSY spectral of 1-(2′-hydroxyphenyl)-3-(2″-bromophenyl)-propan-1-one (**5**) ($CDCl_3$, 151 MHz)

Figure S12. HSQC spectral of 1-(2′-hydroxyphenyl)-3-(2″-bromophenyl)-propan-1-one (**5**) ($CDCl_3$, 151 MHz)

Figure S13. HMBC spectral of 1-(2′-hydroxyphenyl)-3-(2″-bromophenyl)-propan-1-one (**5**) ($CDCl_3$, 151 MHz)

Figure S14. ^{1}H NMR spectral of 1-(2′-hydroxyphenyl)-3-(3″-bromophenyl)-prop-2-en-1-one (**2**) ($CDCl_3$, 600 MHz)

Figure S15. Part of the ^{13}C NMR spectral of 1-(2′-hydroxyphenyl)-3-(3″-bromophenyl)-prop-2-en-1-one (**2**) ($CDCl_3$, 600 MHz)

Figure S16. ^{13}C NMR spectral of 1-(2′-hydroxyphenyl)-3-(3″-bromophenyl)-prop-2-en-1-one (**2**) ($CDCl_3$, 151 MHz)

Figure S17. COSY spectral of 1-(2′-hydroxyphenyl)-3-(3″-bromophenyl)-prop-2-en-1-one (**2**) ($CDCl_3$, 151 MHz)

Figure S18. HSQC spectral of 1-(2′-hydroxyphenyl)-3-(3″-bromophenyl)-prop-2-en-1-one (**2**) ($CDCl_3$, 151 MHz)

Figure S19. ^{1}H NMR spectral of 1-(2′-hydroxyphenyl)-3-(3″-bromophenyl)-propan-1-one (**6**) ($CDCl_3$, 600 MHz)

Figure S20. Part of the ^{13}C NMR spectral of 1-(2′-hydroxyphenyl)-3-(3″-bromophenyl)-propan-1-one (**6**) ($CDCl_3$, 600 MHz)

Figure S21. ^{13}C NMR spectral of 1-(2′-hydroxyphenyl)-3-(3″-bromophenyl)-propan-1-one (**6**) ($CDCl_3$, 151 MHz)

Figure S22. COSY spectral of 1-(2′-hydroxyphenyl)-3-(3″-bromophenyl)-propan-1-one (**6**) ($CDCl_3$, 151 MHz)

Figure S23. HSQC spectral of 1-(2′-hydroxyphenyl)-3-(3″-bromophenyl)-propan-1-one (**6**) ($CDCl_3$, 151 MHz)

Figure S24. HMBC spectral of 1-(2′-hydroxyphenyl)-3-(3″-bromophenyl)-propan-1-one (**6**) ($CDCl_3$, 151 MHz)

Figure S25. ^{1}H NMR spectral of 1-(2′-hydroxyphenyl)-3-(4″-bromophenyl)-prop-2-en-1-one (**3**) ($CDCl_3$, 600 MHz)

Figure S26. Part of the ^{1}H NMR spectral of 1-(2′-hydroxyphenyl)-3-(4″-bromophenyl)-prop-2-en-1-one (**3**) ($CDCl_3$, 600 MHz)

Figure S27. ^{13}C NMR spectral of 1-(2′-hydroxyphenyl)-3-(4″-bromophenyl)-prop-2-en-1-one (**3**) ($CDCl_3$, 151 MHz)

Figure S28. COSY spectral of 1-(2′-hydroxyphenyl)-3-(4″-bromophenyl)-prop-2-en-1-one (**3**) ($CDCl_3$, 151 MHz)

Figure S29. HSQC spectral of 1-(2′-hydroxyphenyl)-3-(4″-bromophenyl)-prop-2-en-1-one (**3**) ($CDCl_3$, 151 MHz)

Figure S30. ^{1}H NMR spectral of 1-(2′-hydroxyphenyl)-3-(4″-bromophenyl)-propan-1-one (**7**) ($CDCl_3$, 600 MHz)

Figure S31. Part of the ^{13}C NMR spectral of 1-(2′-hydroxyphenyl)-3-(4″-bromophenyl)-propan-1-one (**7**) ($CDCl_3$, 600 MHz)

Figure S32. ^{13}C NMR spectral of 1-(2′-hydroxyphenyl)-3-(4″-bromophenyl)-propan-1-one (**7**) ($CDCl_3$, 151 MHz)

Figure S33. COSY spectral of 1-(2′-hydroxyphenyl)-3-(4″-bromophenyl)-propan-1-one (**7**) ($CDCl_3$, 151 MHz)

Figure S34. HSQC spectral of 1-(2′-hydroxyphenyl)-3-(4″-bromophenyl)-propan-1-one (**7**) ($CDCl_3$, 151 MHz)

Figure S35. HMBC spectral of 1-(2′-hydroxyphenyl)-3-(4″-bromophenyl)-propan-1-one (**7**) ($CDCl_3$, 151 MHz)

Figure S36. ^{1}H NMR spectral of 1-(5′-bromo-2′-hydroxyphenyl)-3-phenyl-propan-1-one (**4**) ($CDCl_3$, 600 MHz)

Figure S37. Part of the ^{1}H NMR spectral of 1-(5′-bromo-2′-hydroxyphenyl)-3-phenyl-propan-1-one (**4**) ($CDCl_3$, 600 MHz)

Figure S38. ^{13}C NMR spectral of 1-(5′-bromo-2′-hydroxyphenyl)-3-phenyl-propan-1-one (**4**) ($CDCl_3$, 151 MHz)

Figure S39. COSY spectral of 1-(5′-bromo-2′-hydroxyphenyl)-3-phenyl-propan-1-one (**4**) ($CDCl_3$, 151 MHz)

Figure S40. HSQC spectral of 1-(5′-bromo-2′-hydroxyphenyl)-3-phenyl-propan-1-one (**4**) ($CDCl_3$, 151 MHz)

Figure S41. ^{1}H NMR spectral of 1-(5′-bromo-2′-hydroxyphenyl)-3-phenyl-prop-2-en-1-one (**8**) ($CDCl_3$, 600 MHz)

Figure S42. Part of the ^{13}C NMR spectral of 1-(5′-bromo-2′-hydroxyphenyl)-3-phenyl-prop-2-en-1-one (**8**) ($CDCl_3$, 600 MHz)

Figure S43. ^{13}C NMR spectral of 1-(5′-bromo-2′-hydroxyphenyl)-3-phenyl-prop-2-en-1-one (**8**) ($CDCl_3$, 151 MHz)

Figure S44. COSY spectral of 1-(5′-bromo-2′-hydroxyphenyl)-3-phenyl-prop-2-en-1-one (**8**) ($CDCl_3$, 151 MHz)

Figure S45. HSQC spectral of 1-(5′-bromo-2′-hydroxyphenyl)-3-phenyl-prop-2-en-1-one (**8**) ($CDCl_3$, 151 MHz)

Figure S46. HMBC spectral of 1-(5′-bromo-2′-hydroxyphenyl)-3-phenyl-prop-2-en-1-one (**8**) ($CDCl_3$, 151 MHz)

Figure S47. Preparative TLC plate showing the separation of the post-reaction mixture after biotransformation of 2'-hydroxy-2"-bromochalcone (**1**)

Figure S48. Preparative TLC plate showing the separation of the post-reaction mixture after biotransformation of 2'-hydroxy-3''-bromochalcone (**2**)

Figure S49. Preparative TLC plate showing the separation of the post-reaction mixture after biotransformation of 2'-hydroxy-4''-bromochalcone (**3**)

Figure S50. Preparative TLC plate showing the separation of the post-reaction mixture after biotransformation of 2'-hydroxy-5′-bromochalcone (**4**)

Figure S51. Selected chromatograms showing the composition of the reaction mixtures during the biotransformation of the tested compounds:

a) GC analysis obtained after 12 hours transformation of 2'-hydroxy-2''-bromochalcone (**1**)

b) GC analysis obtained after 3 days transformation of 2'-hydroxy-2''-bromochalcone (**2**)

c) GC analysis obtained after 3 hours transformation of 2'-hydroxy-5′-bromochalcone (**4**)

d) GC analysis obtained after 10 days transformation of 2'-hydroxy-5′-bromochalcone (**4**)

Figure S1. ^{1}H NMR spectral of 1-(2'-hydroxyphenyl)-3-(2"-bromophenyl)-prop-2-en-1-one (**1**) ($CDCl_3$, 600 MHz)

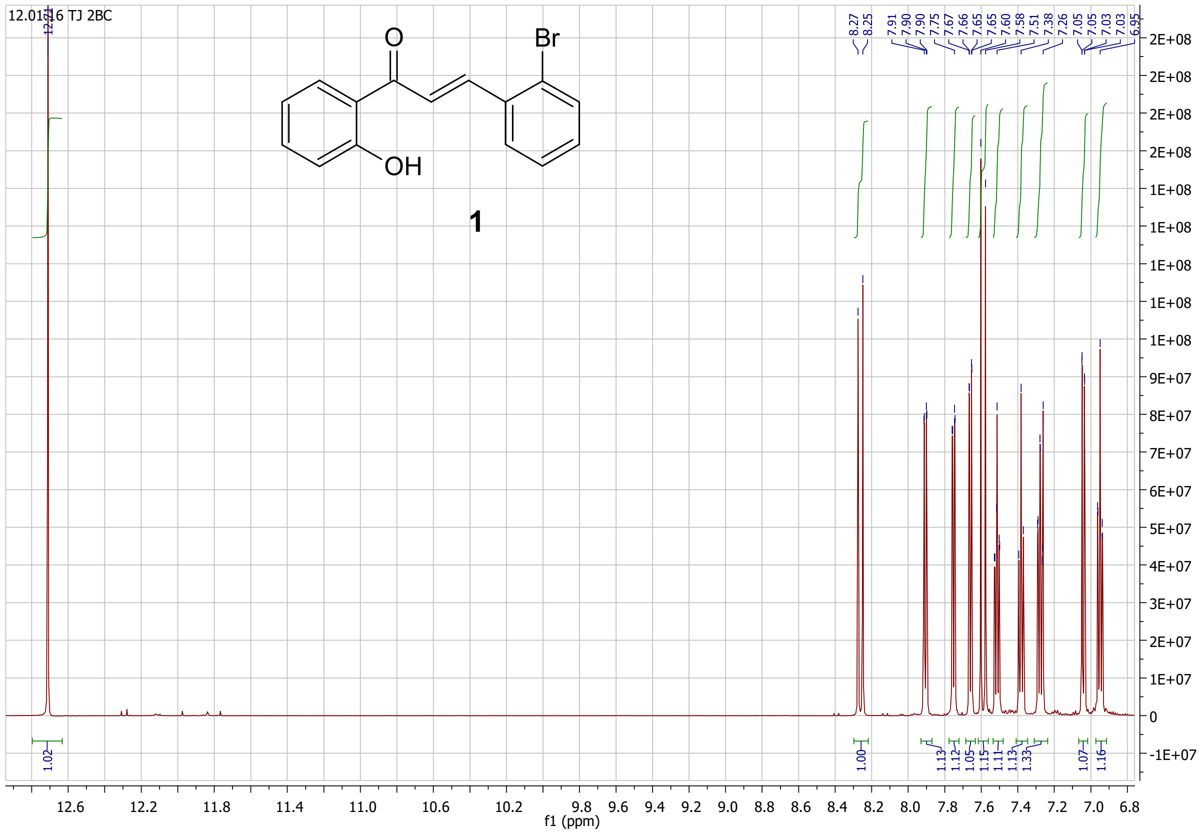

Figure S2. Part of the ^{13}C NMR spectral of 1-(2'-hydroxyphenyl)-3-(2"-bromophenyl)-prop-2-en-1-one (**1**) ($CDCl_3$, 600 MHz)

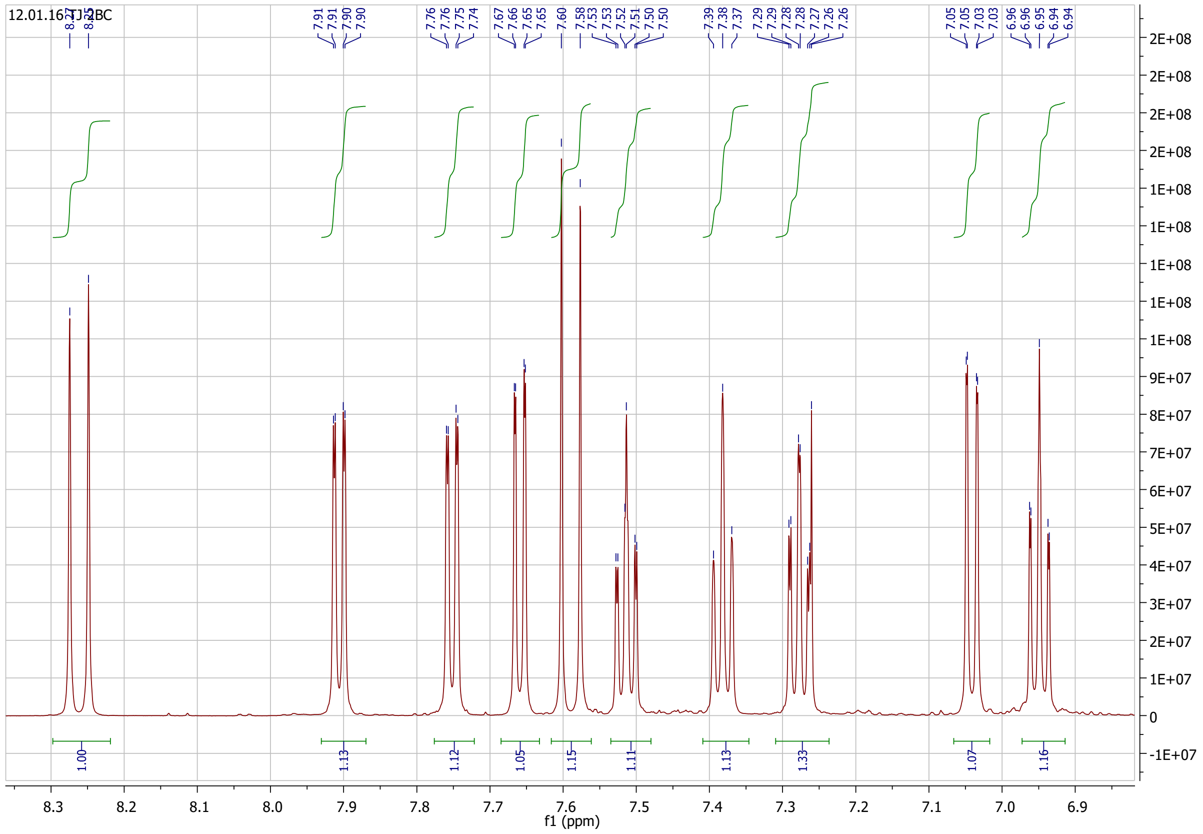

Figure S3. ^{13}C NMR spectral of 1-(2'-hydroxyphenyl)-3-(2"-bromophenyl)-prop-2-en-1-one (**1**) ($CDCl_3$, 151 MHz)

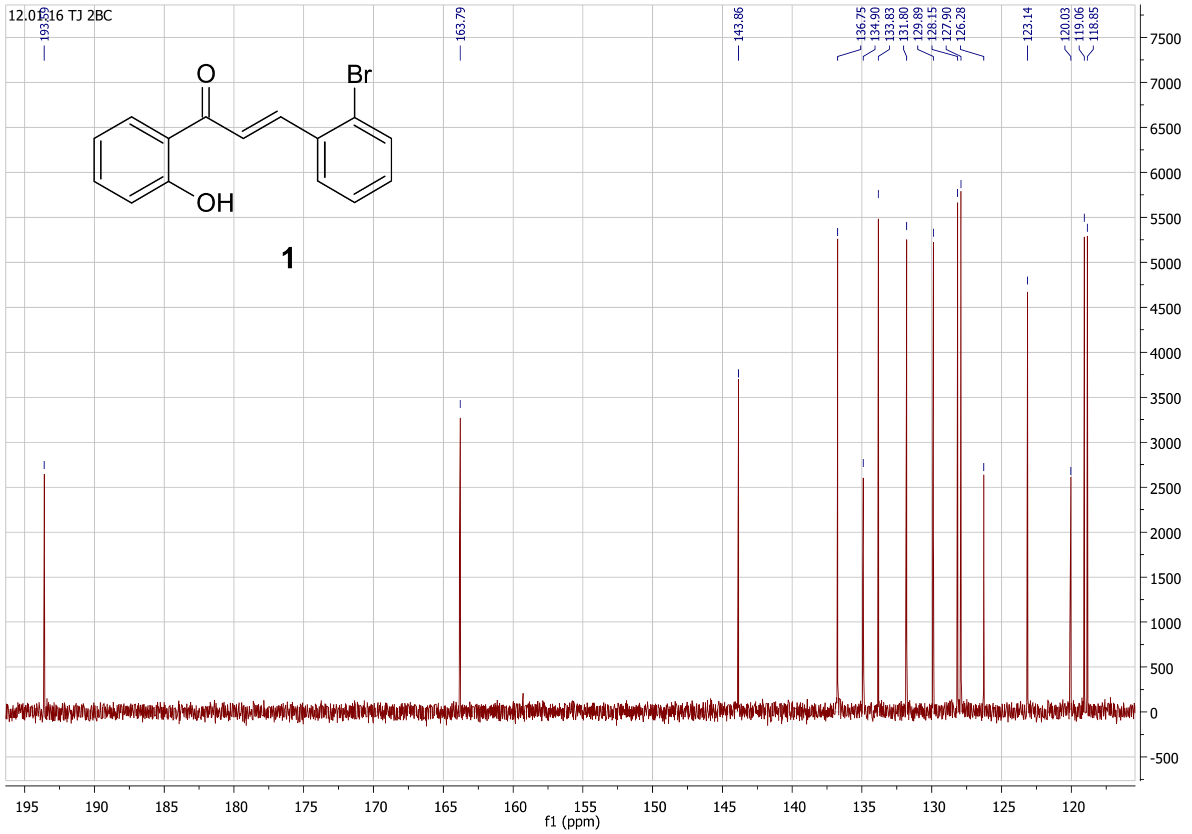

Figure S4. COSY spectral of 1-(2'-hydroxyphenyl)-3-(2"-bromophenyl)-prop-2-en-1-one (**1**) ($CDCl_3$, 151 MHz)

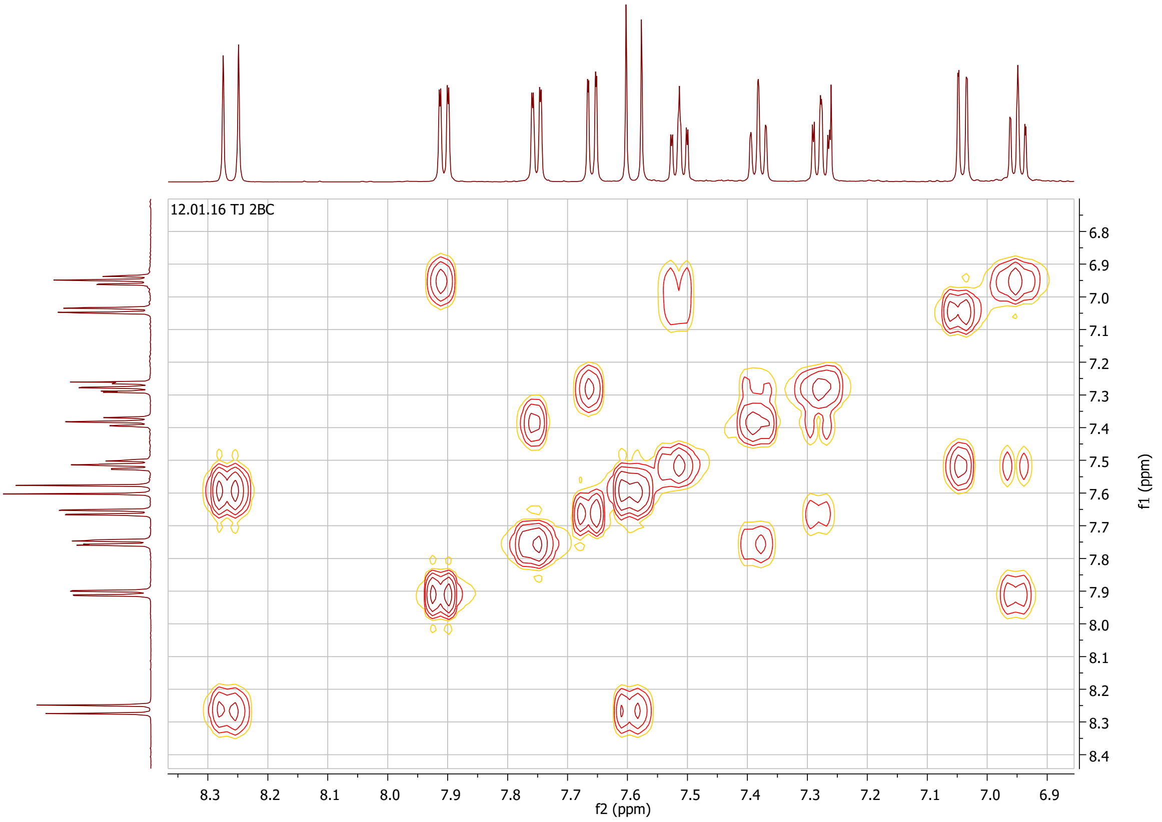

Figure S5. HSQC spectral of 1-(2'-hydroxyphenyl)-3-(2''-bromophenyl)-prop-2-en-1-one (**1**) ($CDCl_3$, 151 MHz)

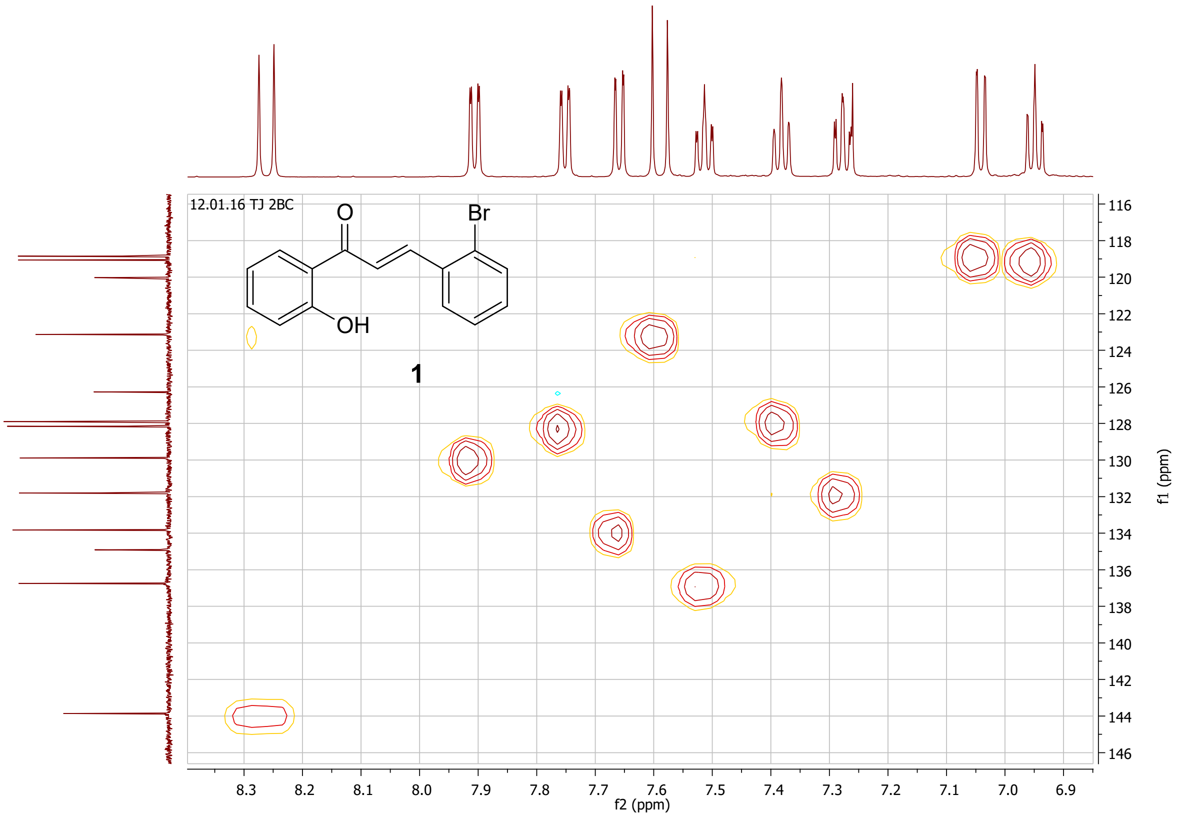

Figure S6. ^{1}H NMR spectral of 1-(2'-hydroxyphenyl)-3-(2''-bromophenyl)-propan-1-one (**5**) ($CDCl_3$, 600 MHz)

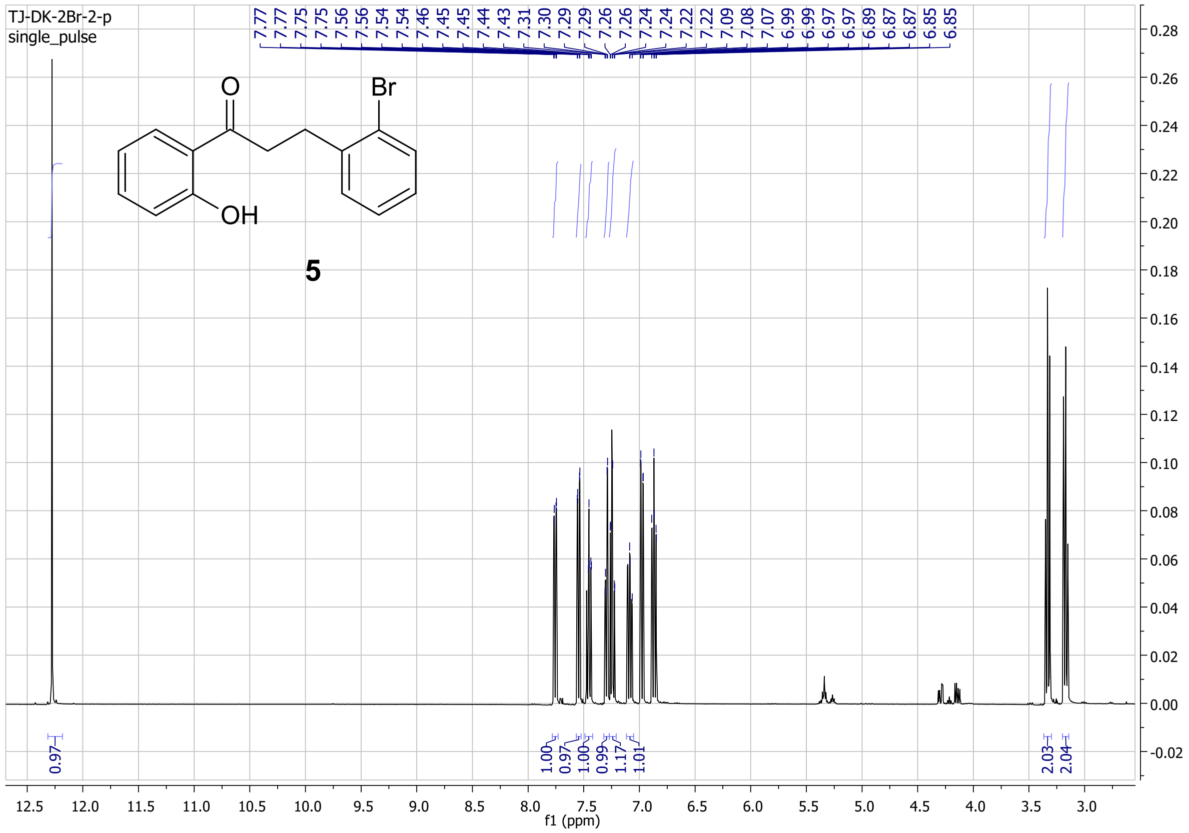

Figure S7. Part of the ^{13}C NMR spectral of 1-(2'-hydroxyphenyl)-3-(2"-bromophenyl)-propan-1-one (**5**) ($CDCl_3$, 600 MHz)

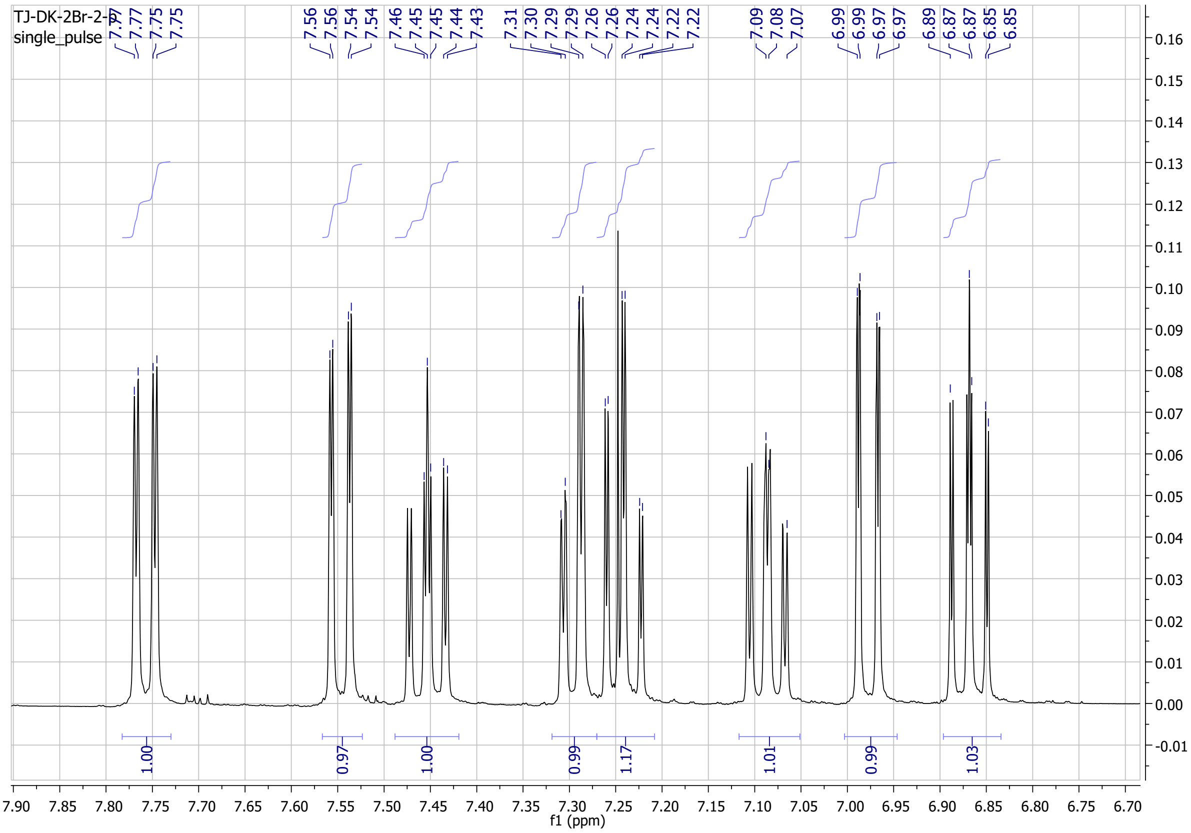

Figure S8. ^{13}C NMR spectral of 1-(2'-hydroxyphenyl)-3-(2"-bromophenyl)-propan-1-one (**5**) ($CDCl_3$, 151 MHz)

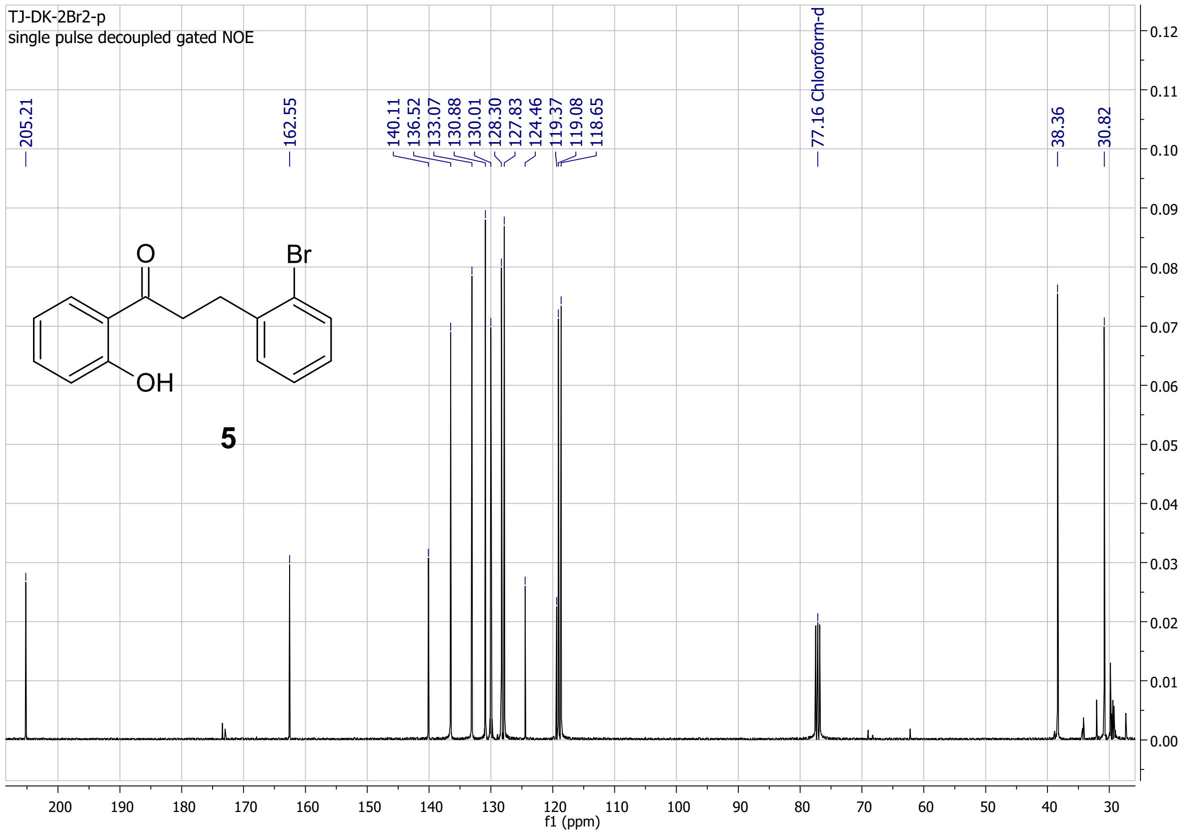

Figure S9. Part of the ^{13}C NMR spectral of 1-(2'-hydroxyphenyl)-3-(2''-bromophenyl)-propan-1-one (**5**) ($CDCl_3$, 151 MHz)

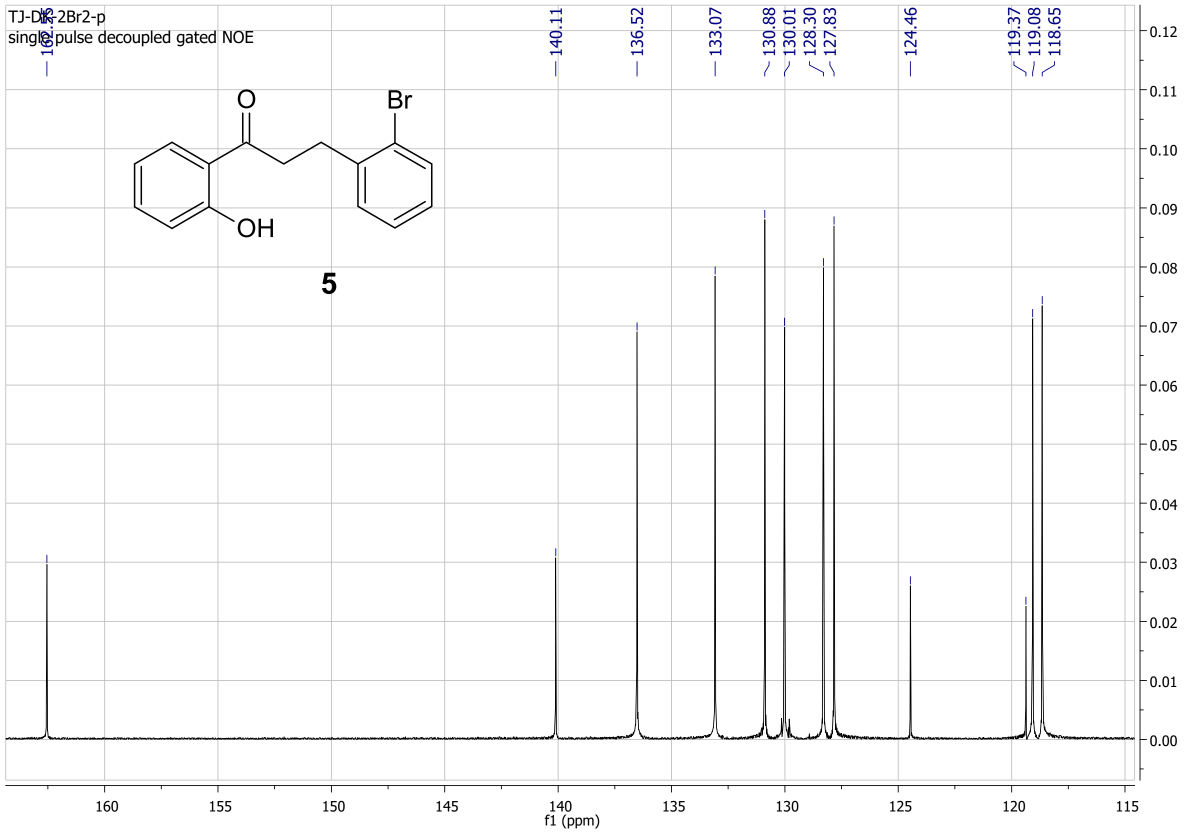

Figure S10. COSY spectral of 1-(2'-hydroxyphenyl)-3-(2''-bromophenyl)-propan-1-one (**5**) ($CDCl_3$, 151 MHz)

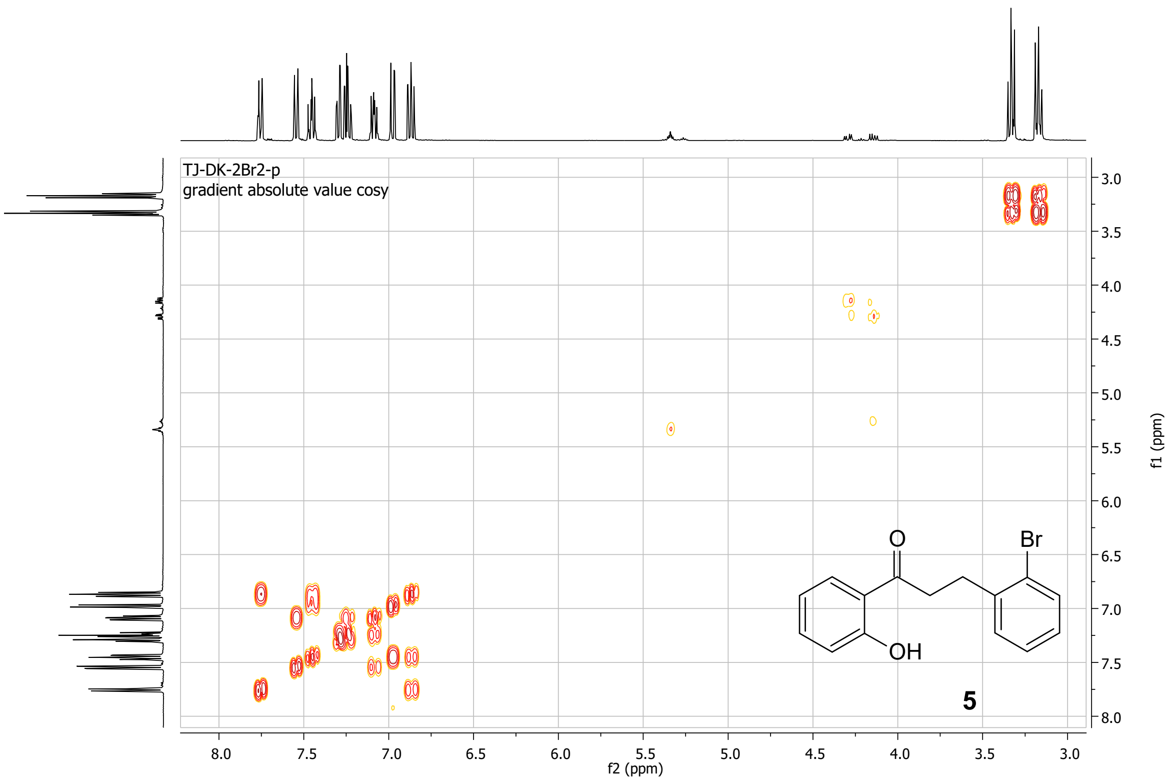

Figure S11. Part of the COSY spectral of 1-(2'-hydroxyphenyl)-3-(2"-bromophenyl)-propan-1-one (**5**) ($CDCl_3$, 151 MHz)

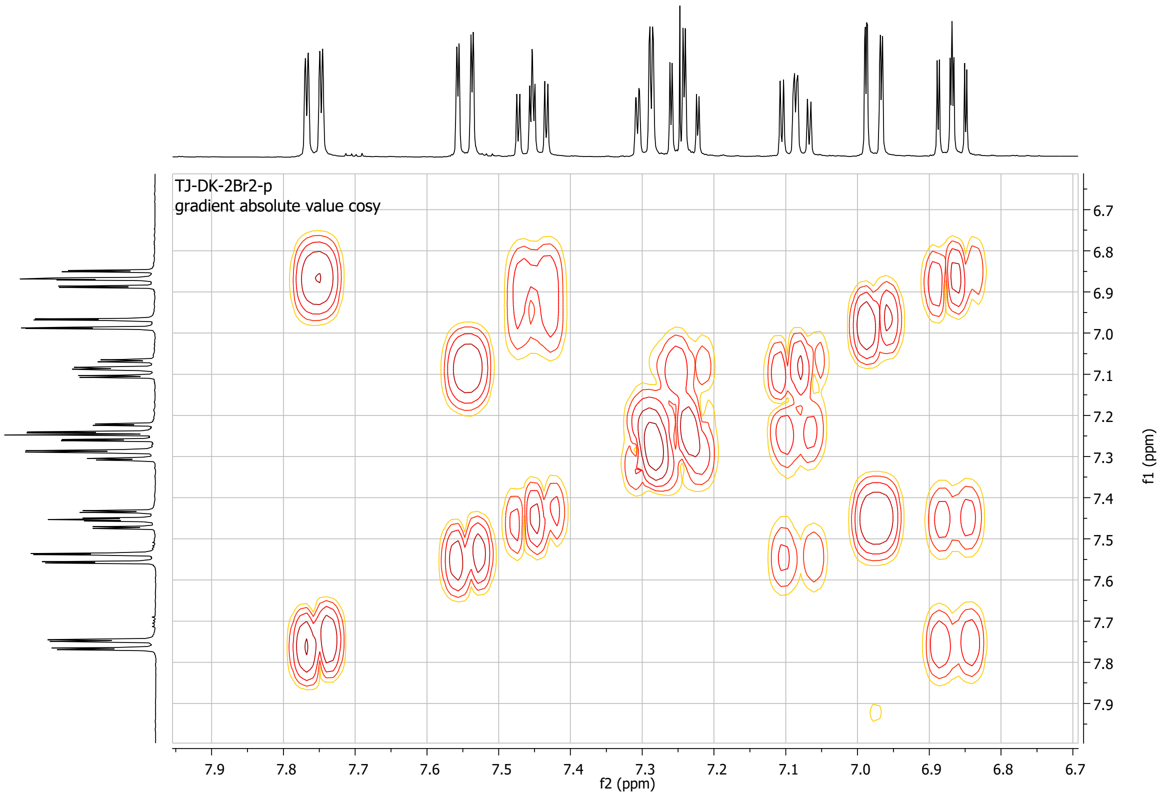

Figure S12. HSQC spectral of 1-(2'-hydroxyphenyl)-3-(2"-bromophenyl)-propan-1-one (**5**) ($CDCl_3$, 151 MHz)

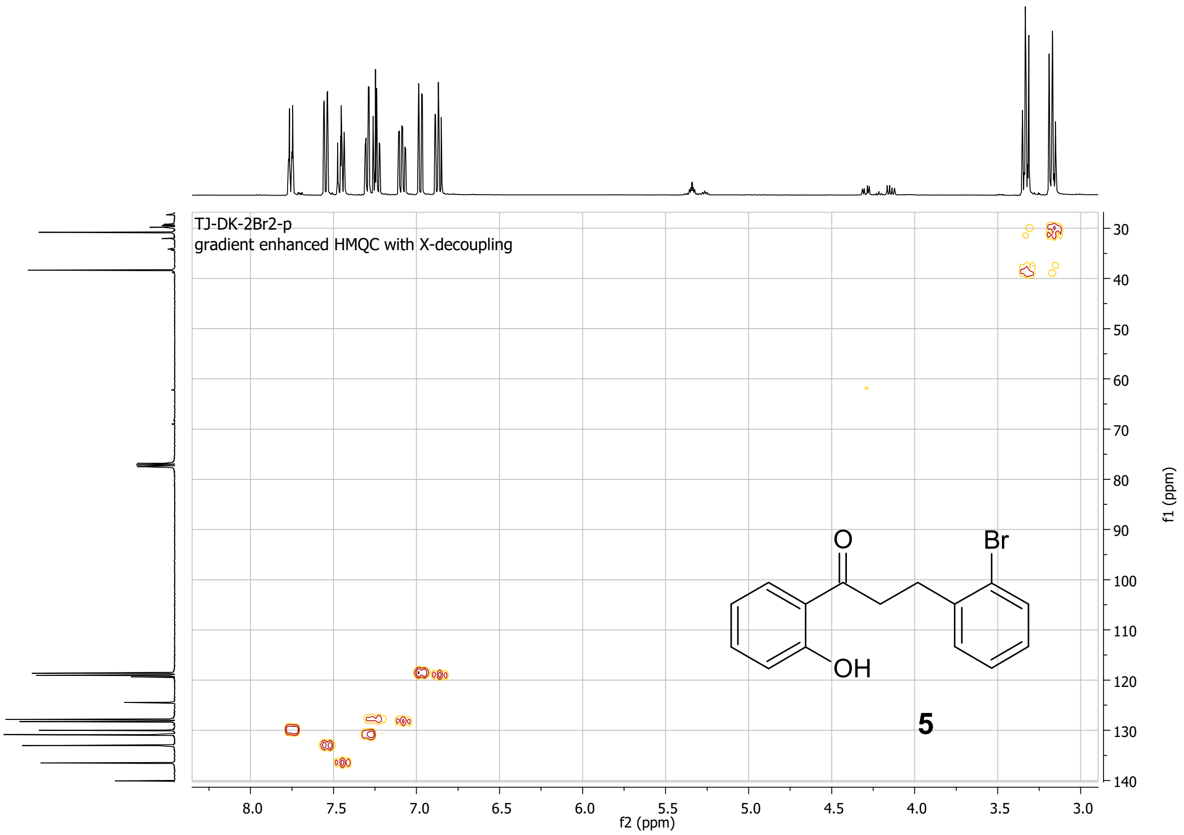

Figure S13. HMBC spectral of 1-(2’-hydroxyphenyl)-3-(2”-bromophenyl)-propan-1-one (**5**) ($CDCl_3$, 151 MHz)

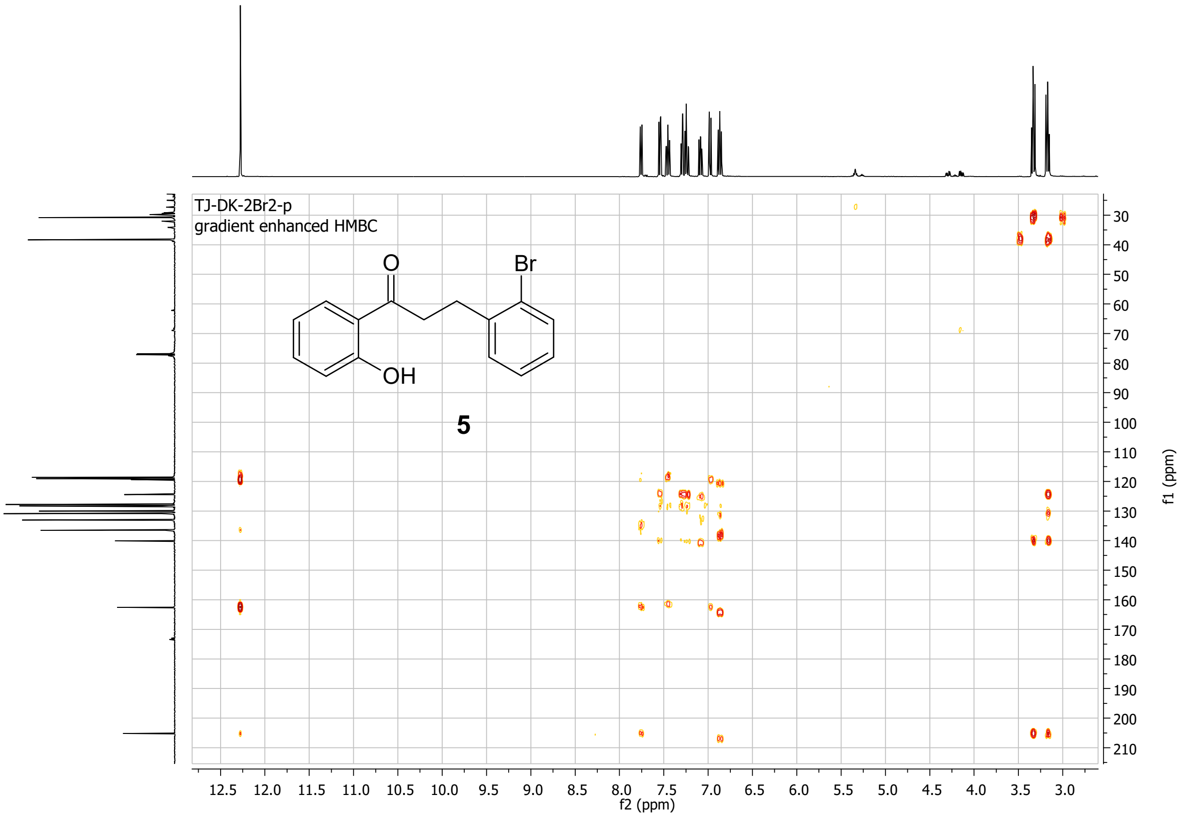

Figure S14. ^{1}H NMR spectral of 1-(2’-hydroxyphenyl)-3-(3”-bromophenyl)-prop-2-en-1-one (**2**) ($CDCl_3$, 600 MHz)

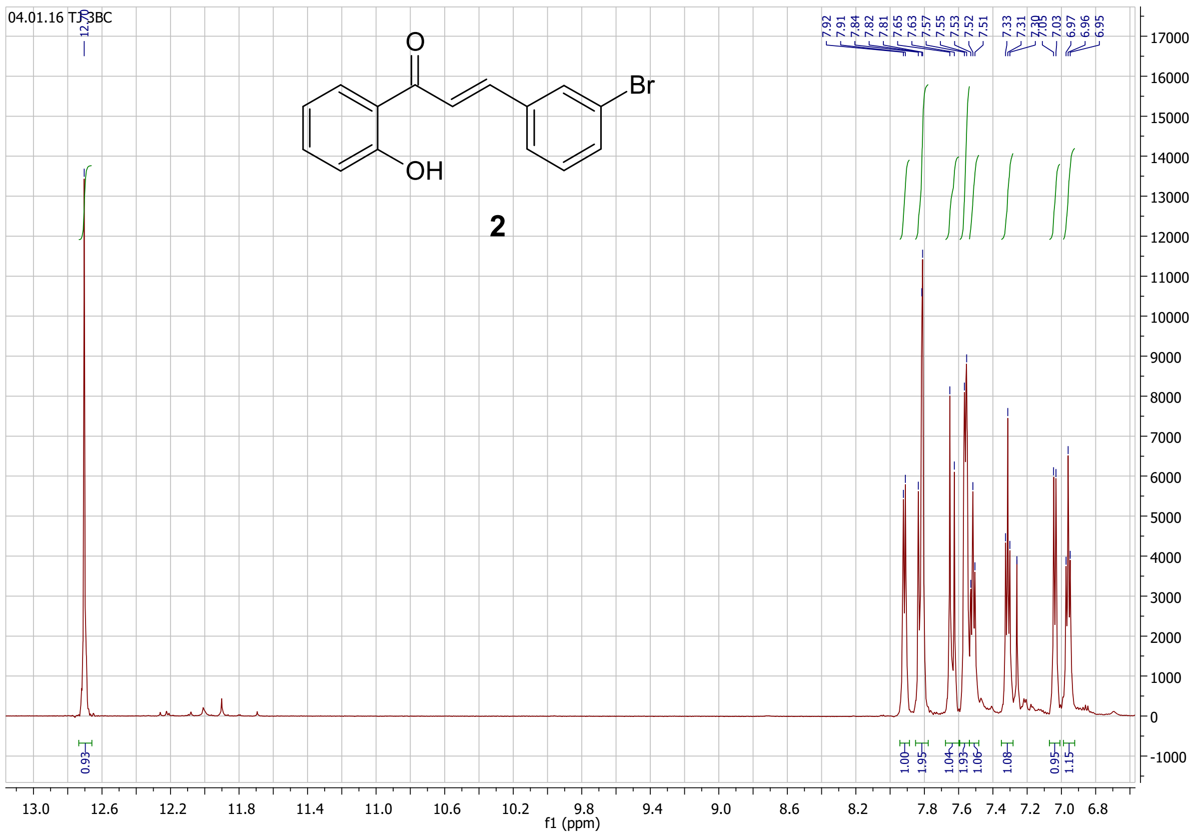

Figure S15. Part of the ^{13}C NMR spectral of 1-(2’-hydroxyphenyl)-3-(3”-bromophenyl)-prop-2-en-1-one (**2**) ($CDCl_3$, 600 MHz)

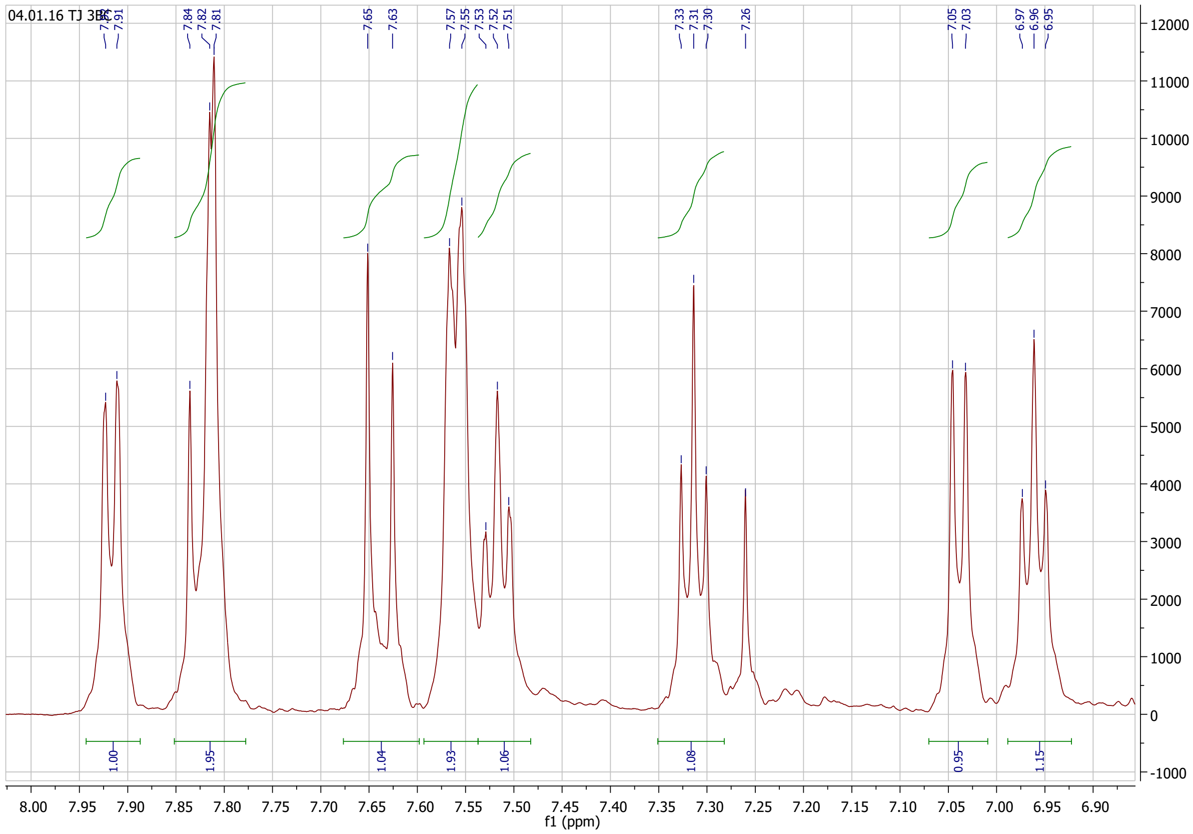

Figure S16. ^{13}C NMR spectral of 1-(2’-hydroxyphenyl)-3-(3”-bromophenyl)-prop-2-en-1-one (**2**) ($CDCl_3$, 151 MHz)

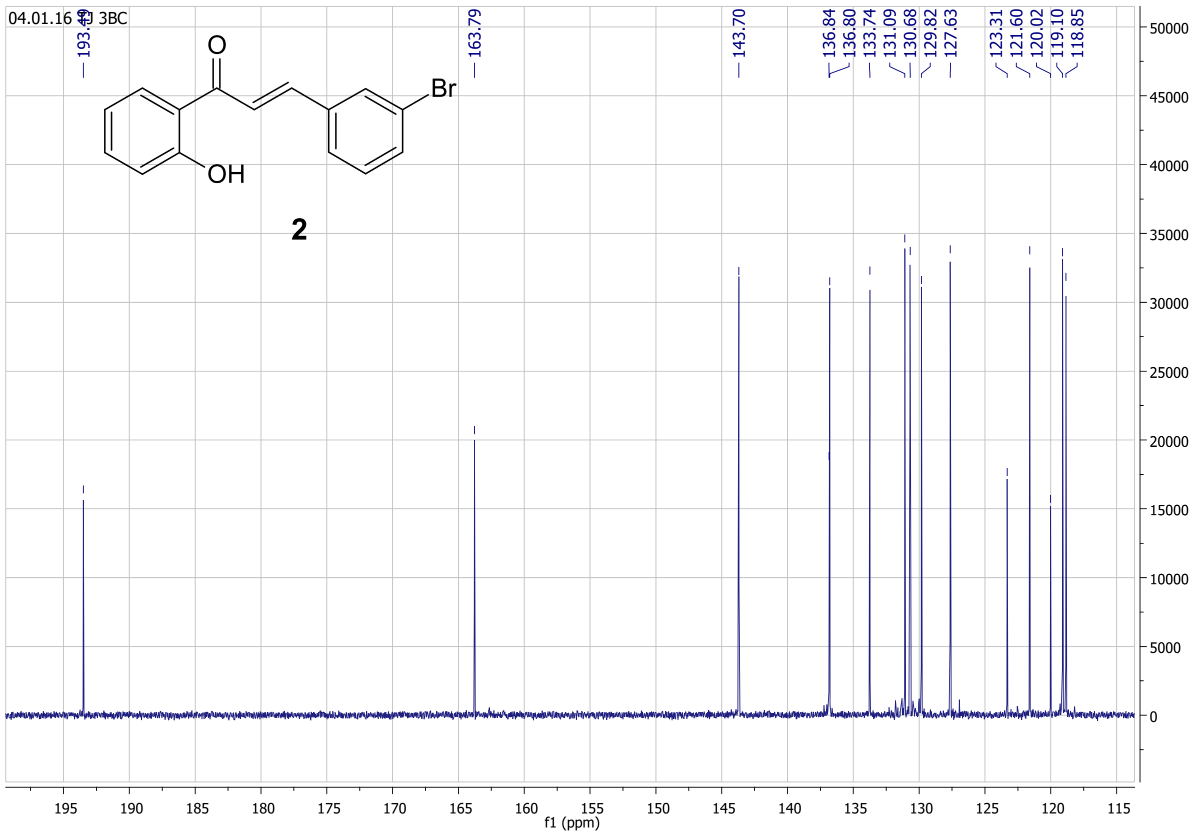

Figure S17. COSY spectral of 1-(2’-hydroxyphenyl)-3-(3”-bromophenyl)-prop-2-en-1-one (**2**) ($CDCl_3$, 151 MHz)

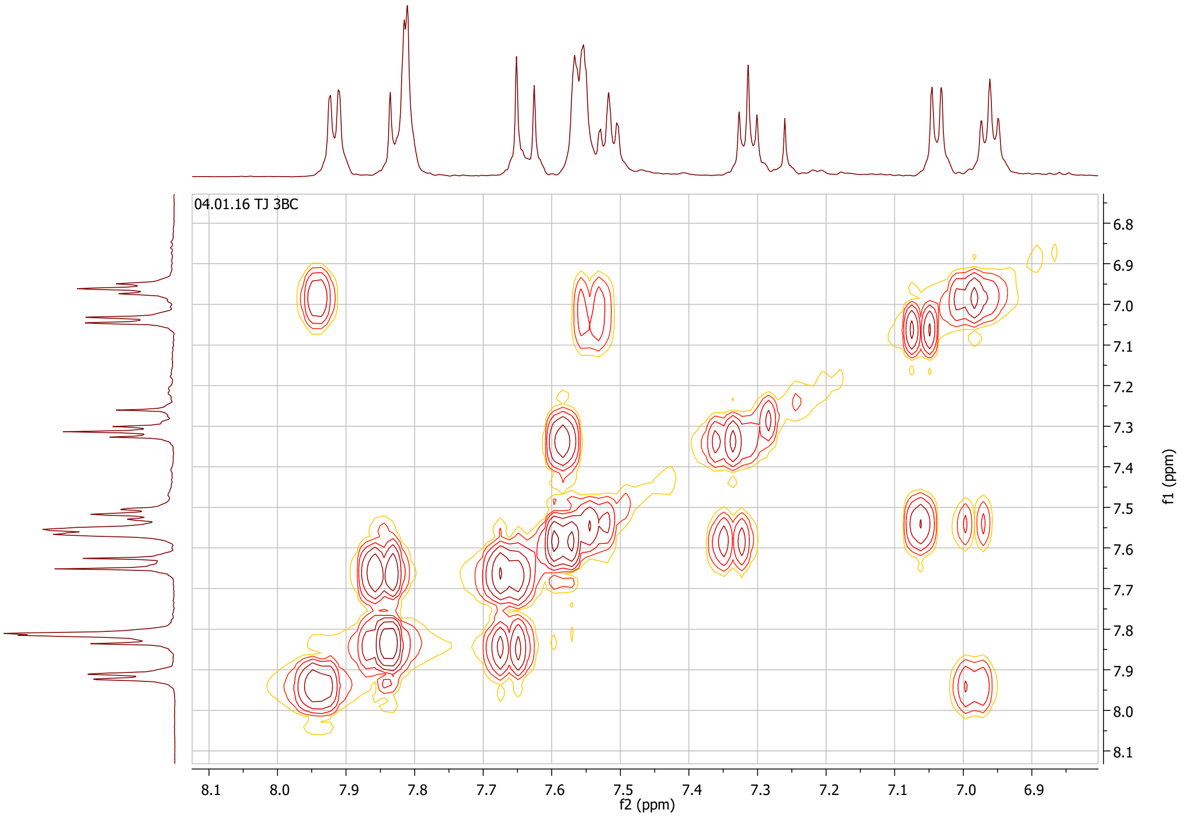

Figure S18. HSQC spectral of 1-(2’-hydroxyphenyl)-3-(3”-bromophenyl)-prop-2-en-1-one (**2**) ($CDCl_3$, 151 MHz)

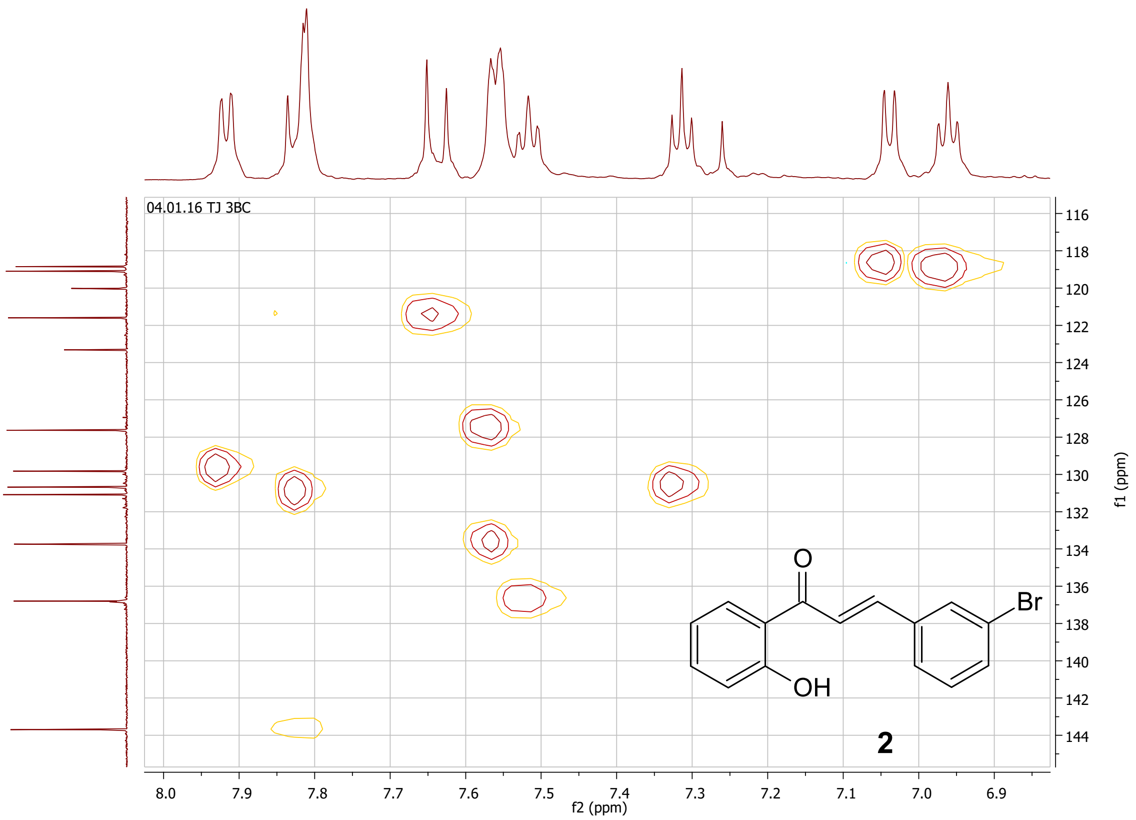

Figure S19. ^{1}H NMR spectral of 1-(2'-hydroxyphenyl)-3-(3''-bromophenyl)-propan-1-one (**6**) ($CDCl_3$, 600 MHz)

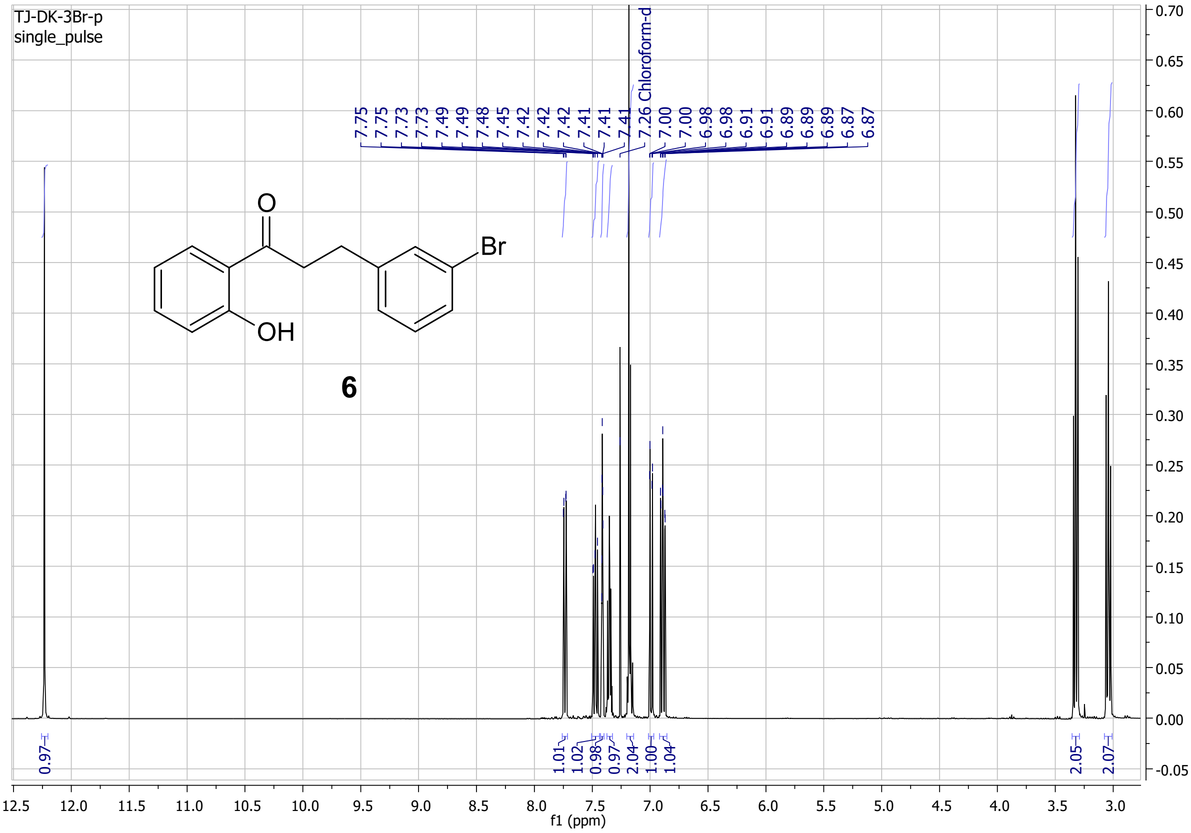

Figure S20. Part of the ^{13}C NMR spectral of 1-(2’-hydroxyphenyl)-3-(3”-bromophenyl)-propan-1-one (**6**) ($CDCl_3$, 600 MHz)

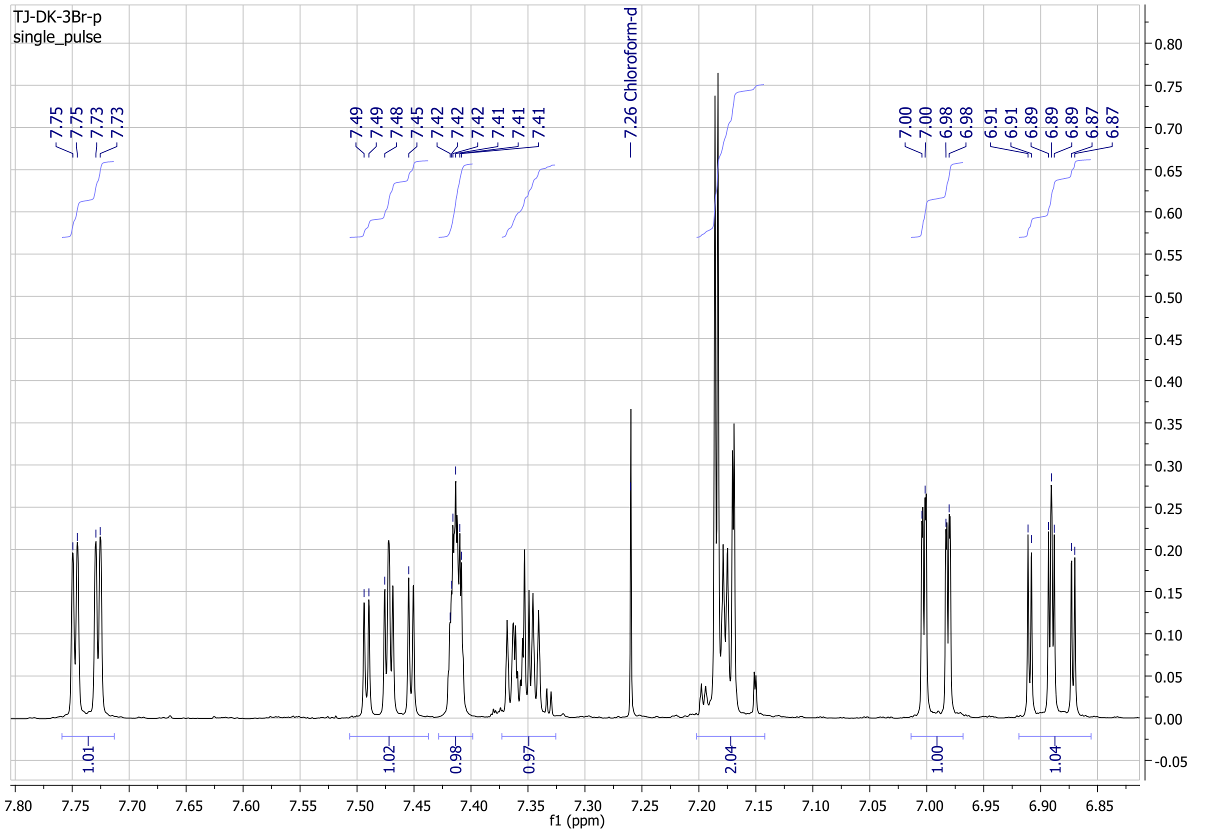

Figure S21. ^{13}C NMR spectral of 1-(2’-hydroxyphenyl)-3-(3”-bromophenyl)-propan-1-one (**6**) ($CDCl_3$, 151 MHz)

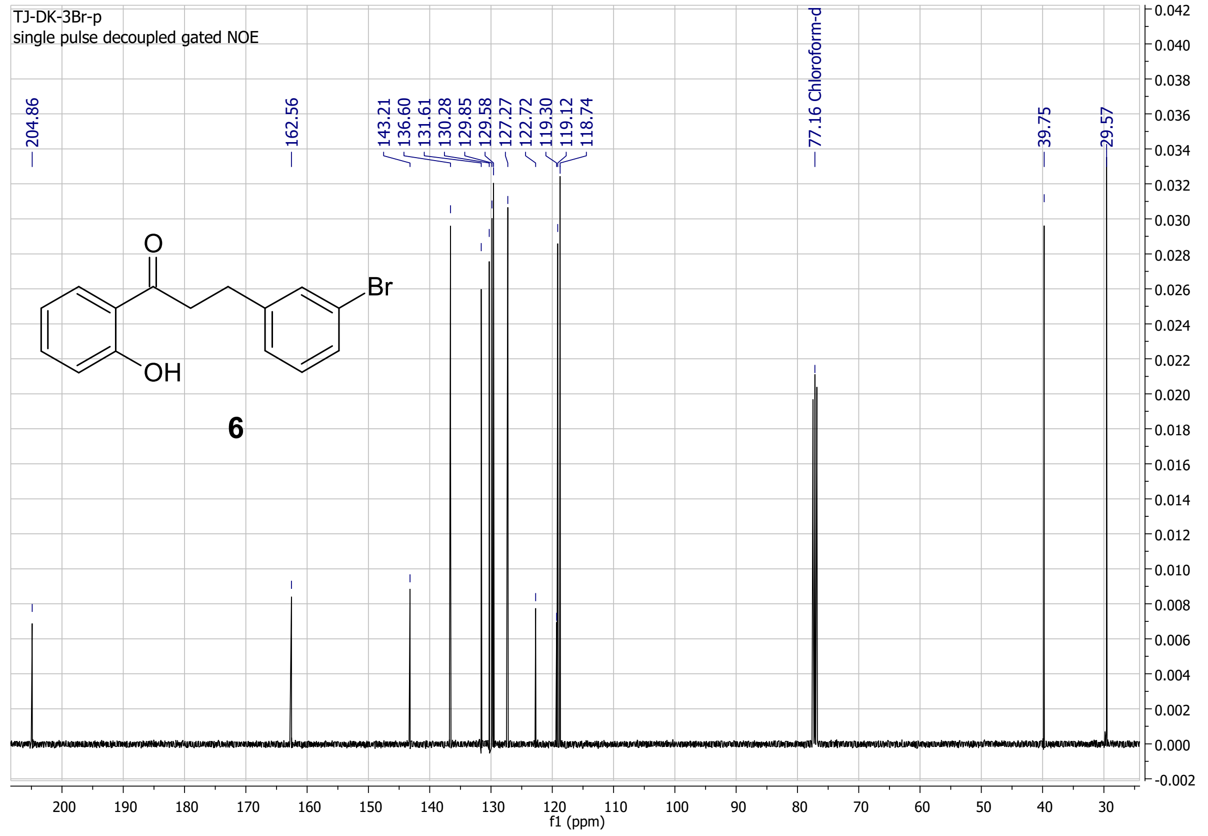

Figure S22. COSY spectral of 1-(2'-hydroxyphenyl)-3-(3"-bromophenyl)-propan-1-one (**6**) ($CDCl_3$, 151 MHz)

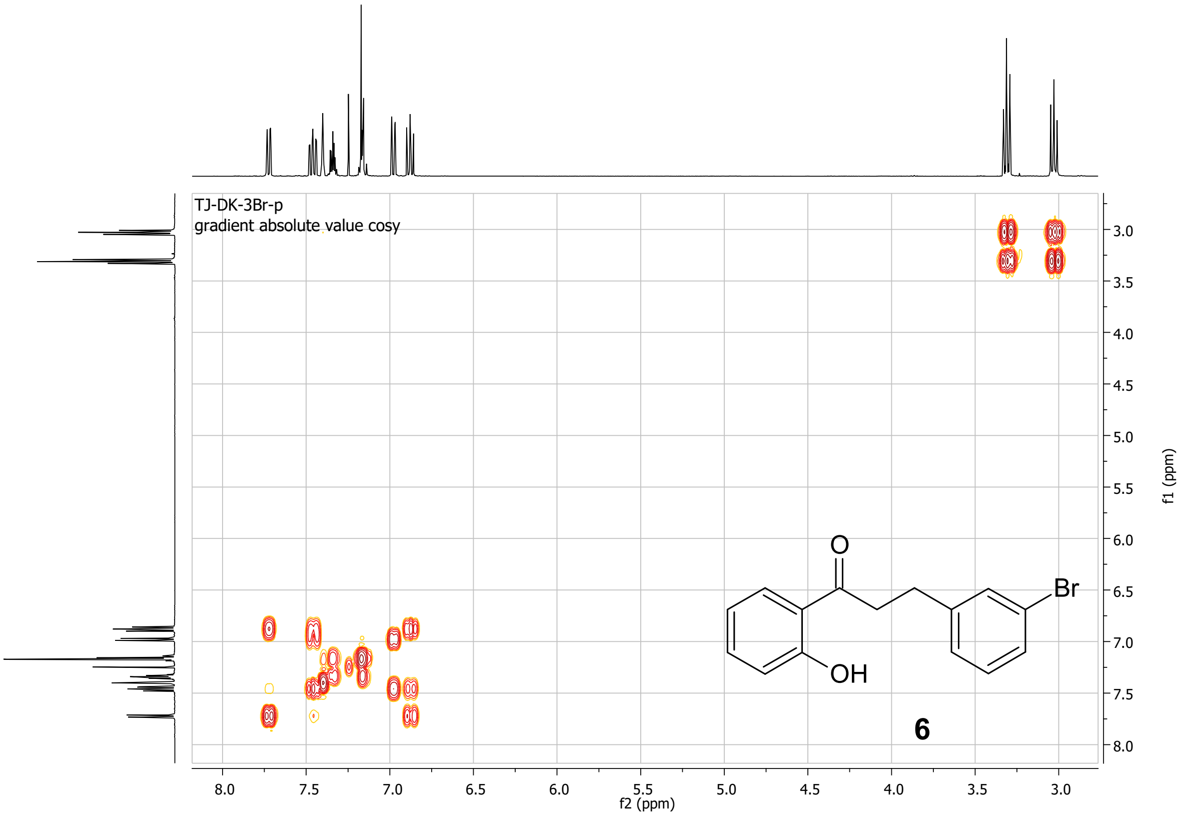

Figure S23. HSQC spectral of 1-(2'-hydroxyphenyl)-3-(3"-bromophenyl)-propan-1-one (**6**) ($CDCl_3$, 151 MHz)

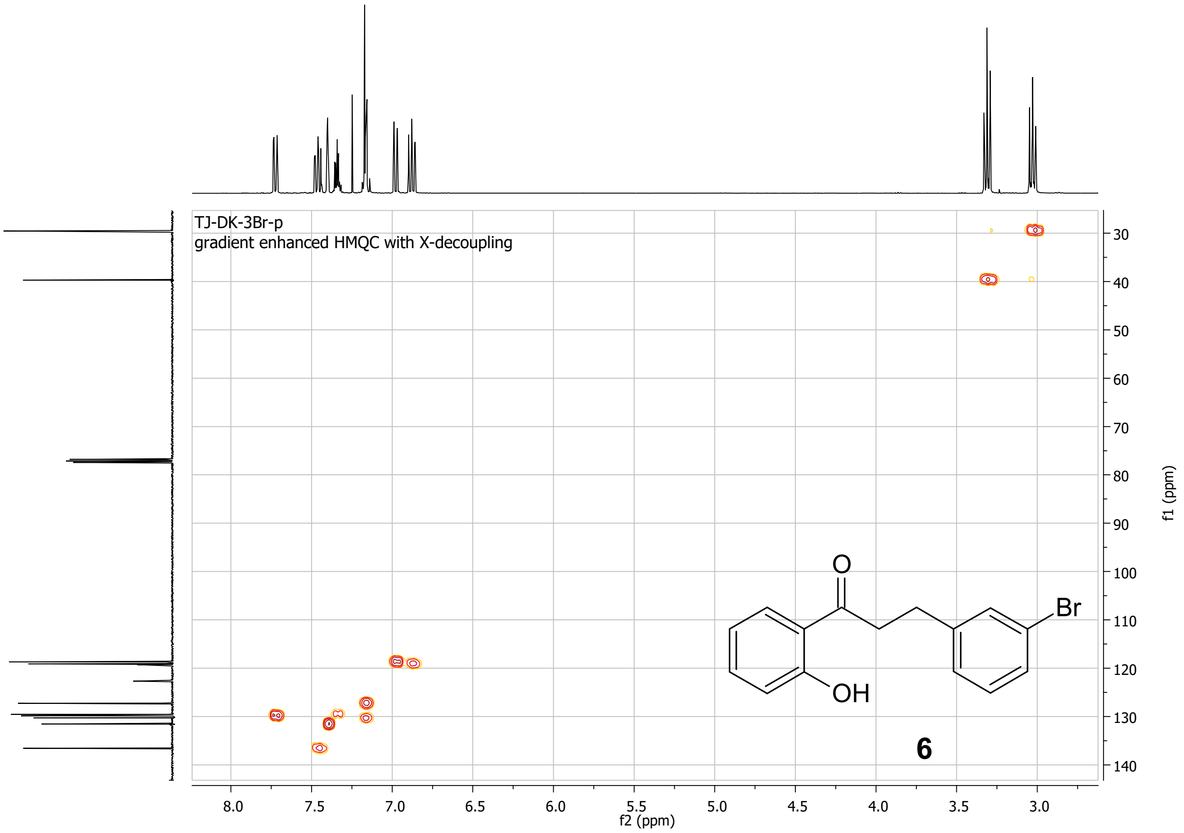

Figure S24. HMBC spectral of 1-(2'-hydroxyphenyl)-3-(3''-bromophenyl)-propan-1-one (**6**) ($CDCl_3$, 151 MHz)

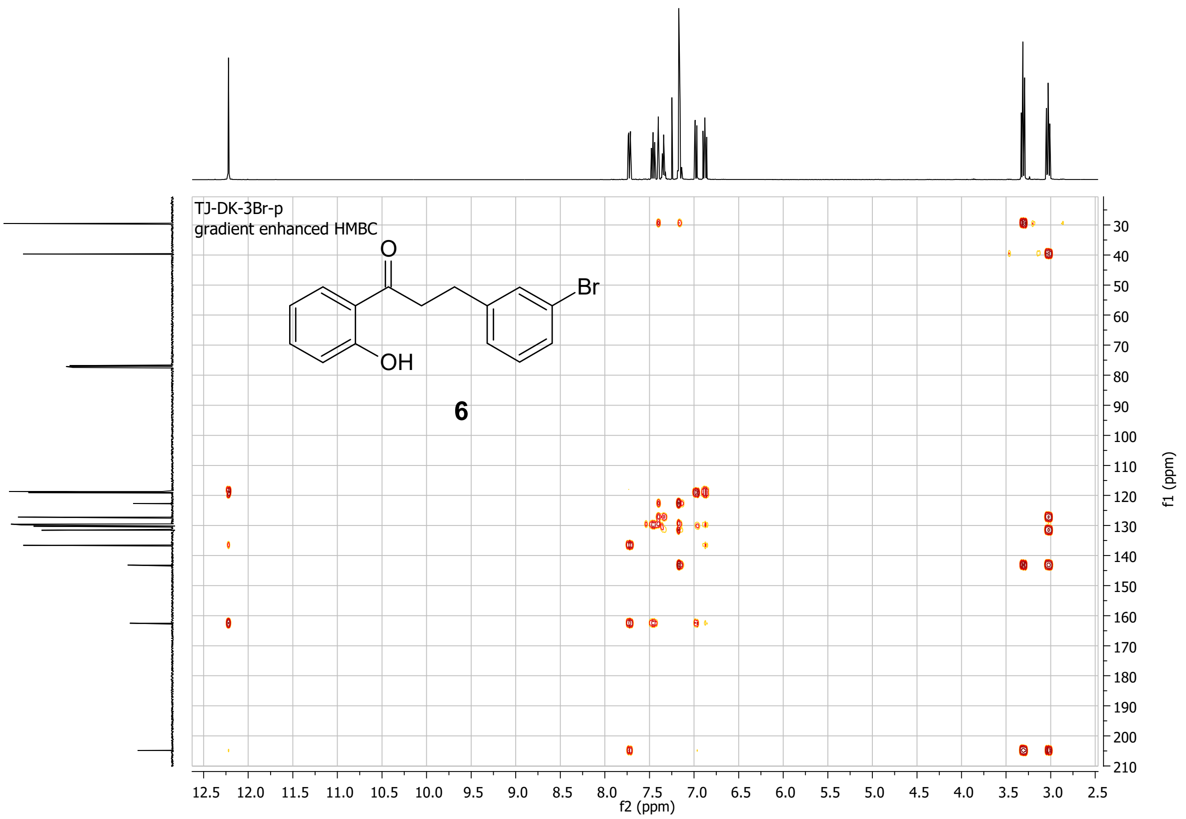

Figure S25. ^{1}H NMR spectral of 1-(2'-hydroxyphenyl)-3-(4''-bromophenyl)-prop-2-en-1-one (**3**) ($CDCl_3$, 600 MHz)

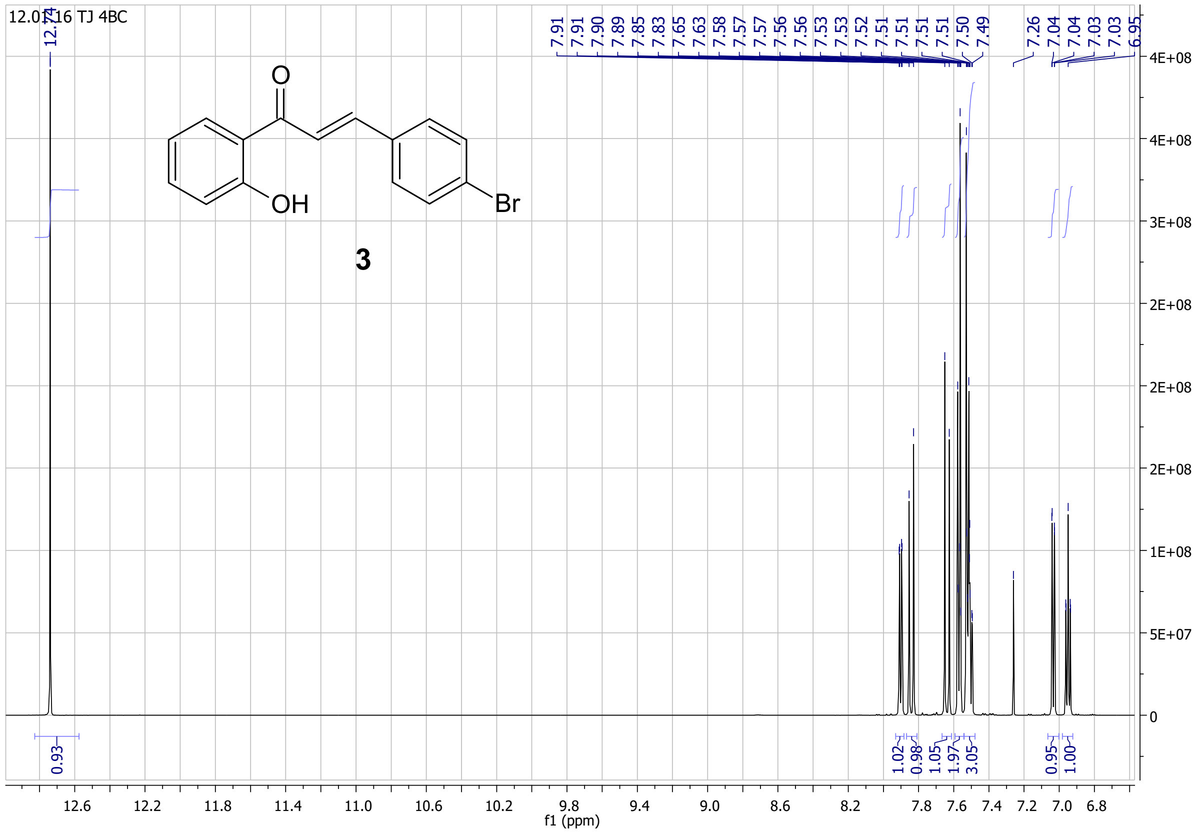

Figure S26. Part of the ^{1}H NMR spectral of 1-(2'-hydroxyphenyl)-3-(4''-bromophenyl)-prop-2-en-1-one (**3**) ($CDCl_3$, 600 MHz)

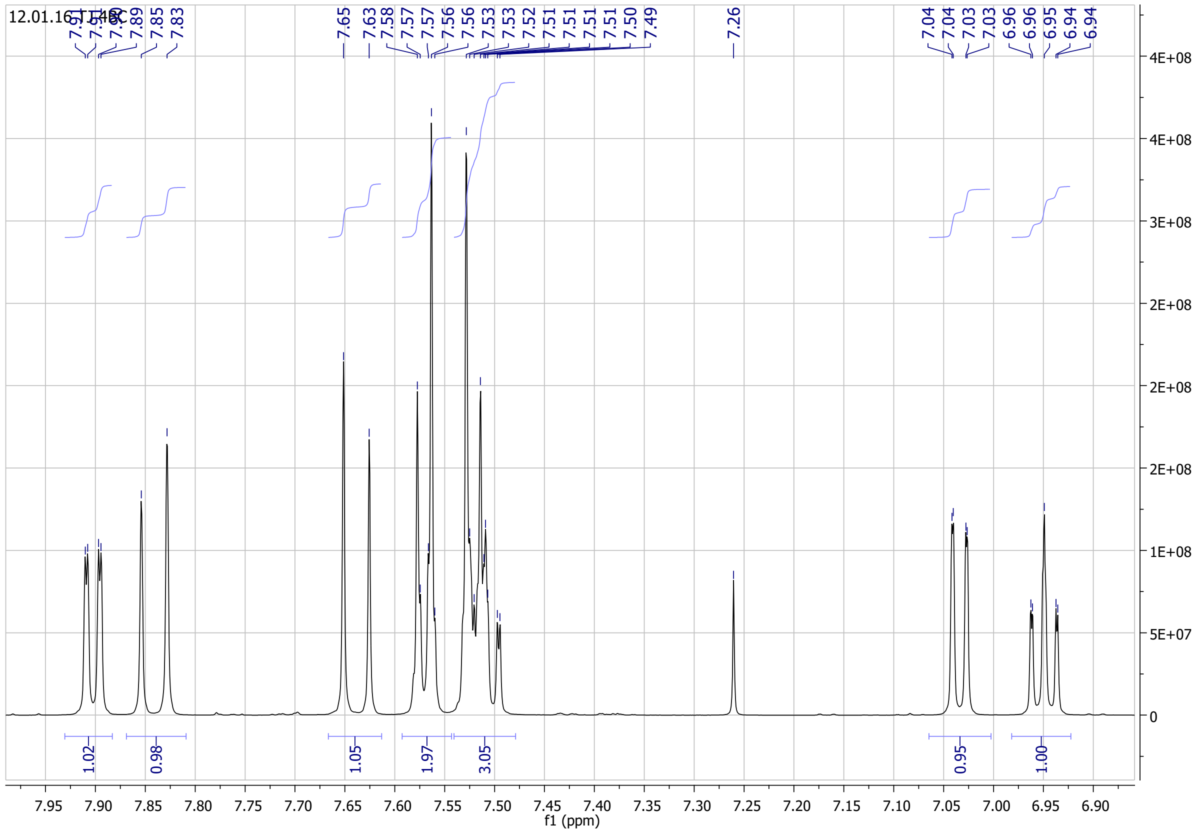

Figure S27. ^{13}C NMR spectral of 1-(2'-hydroxyphenyl)-3-(4''-bromophenyl)-prop-2-en-1-one (**3**) ($CDCl_3$, 151 MHz)

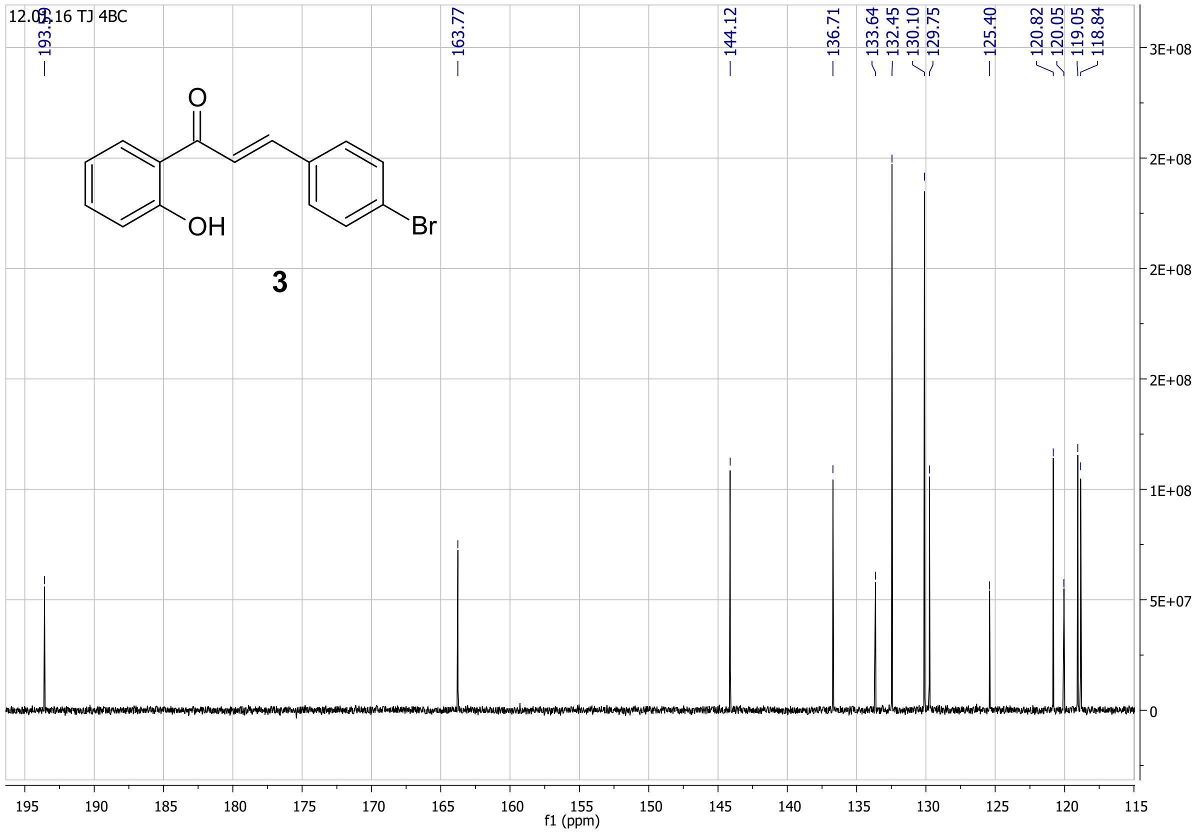

Figure S28. COSY spectral of 1-(2’-hydroxyphenyl)-3-(4”-bromophenyl)-prop-2-en-1-one (**3**) ($CDCl_3$, 151 MHz)

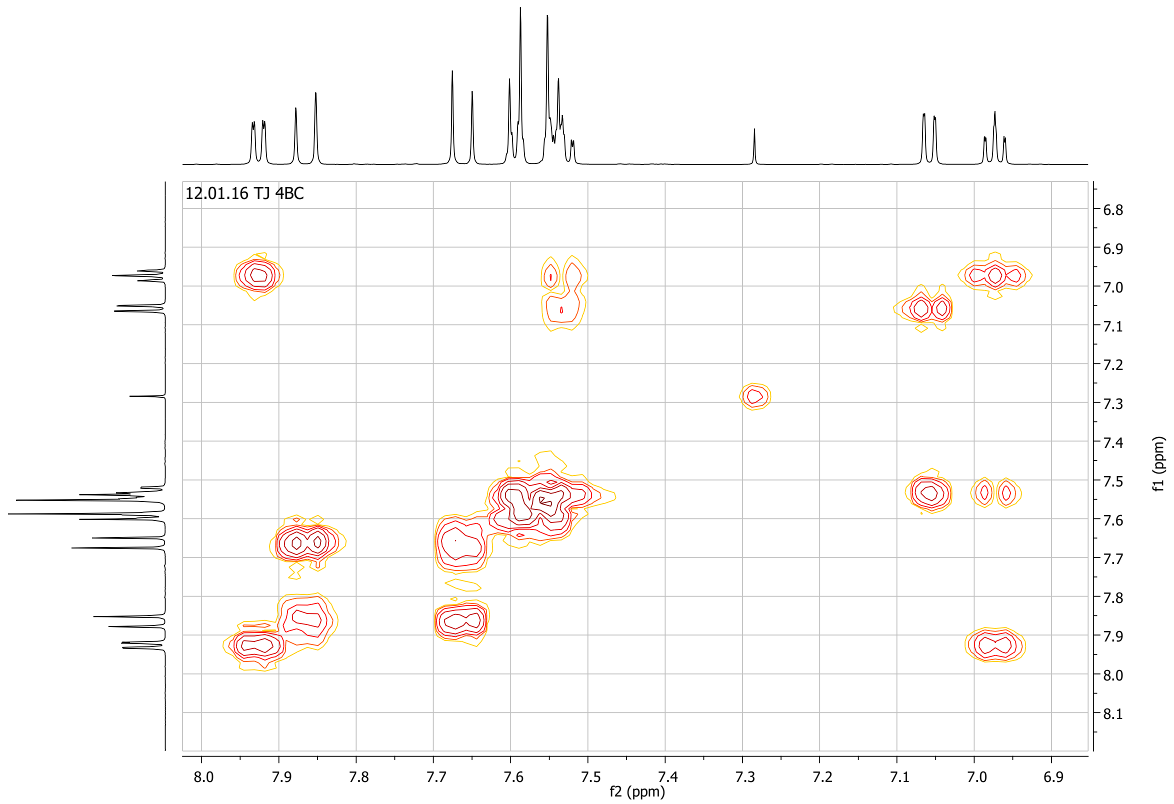

Figure S29. HSQC spectral of 1-(2’-hydroxyphenyl)-3-(4”-bromophenyl)-prop-2-en-1-one (**3**) ($CDCl_3$, 151 MHz)

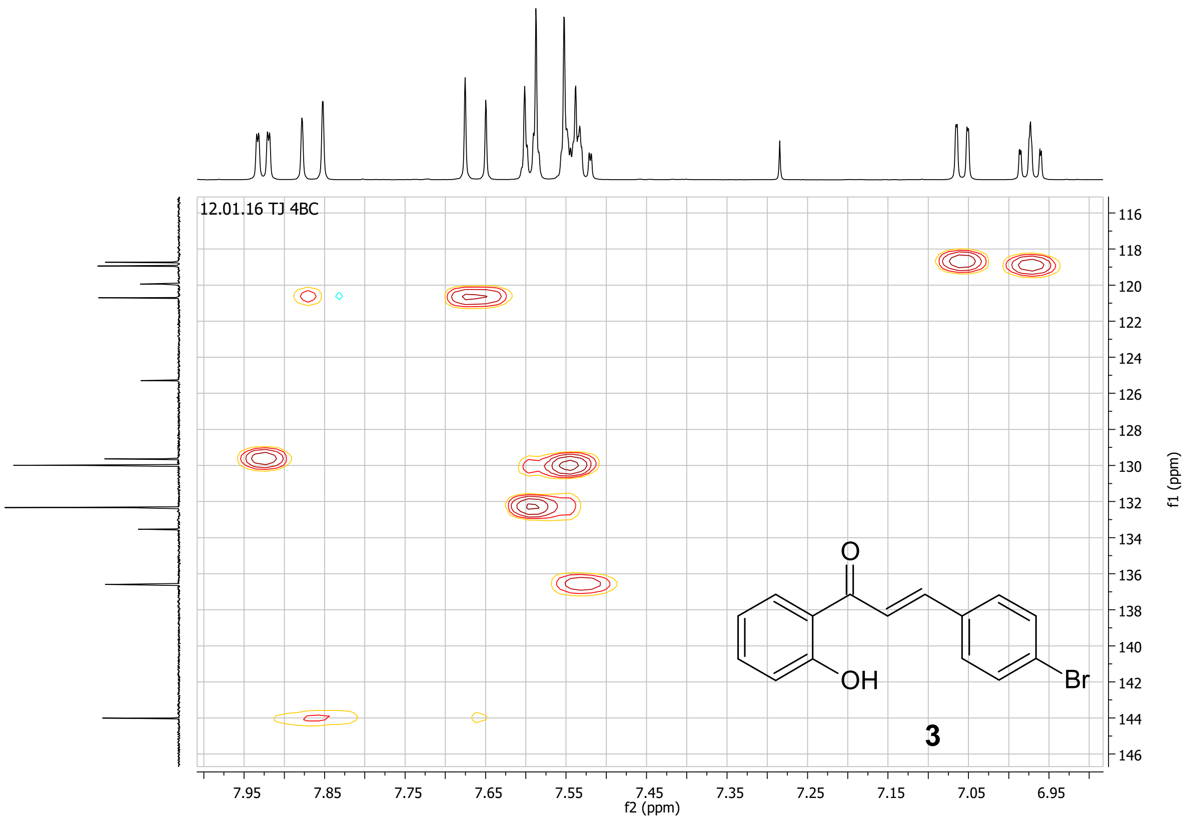

Figure S30. ^{1}H NMR spectral of 1-(2’-hydroxyphenyl)-3-(4”-bromophenyl)-propan-1-one (**7**) ($CDCl_3$, 600 MHz)

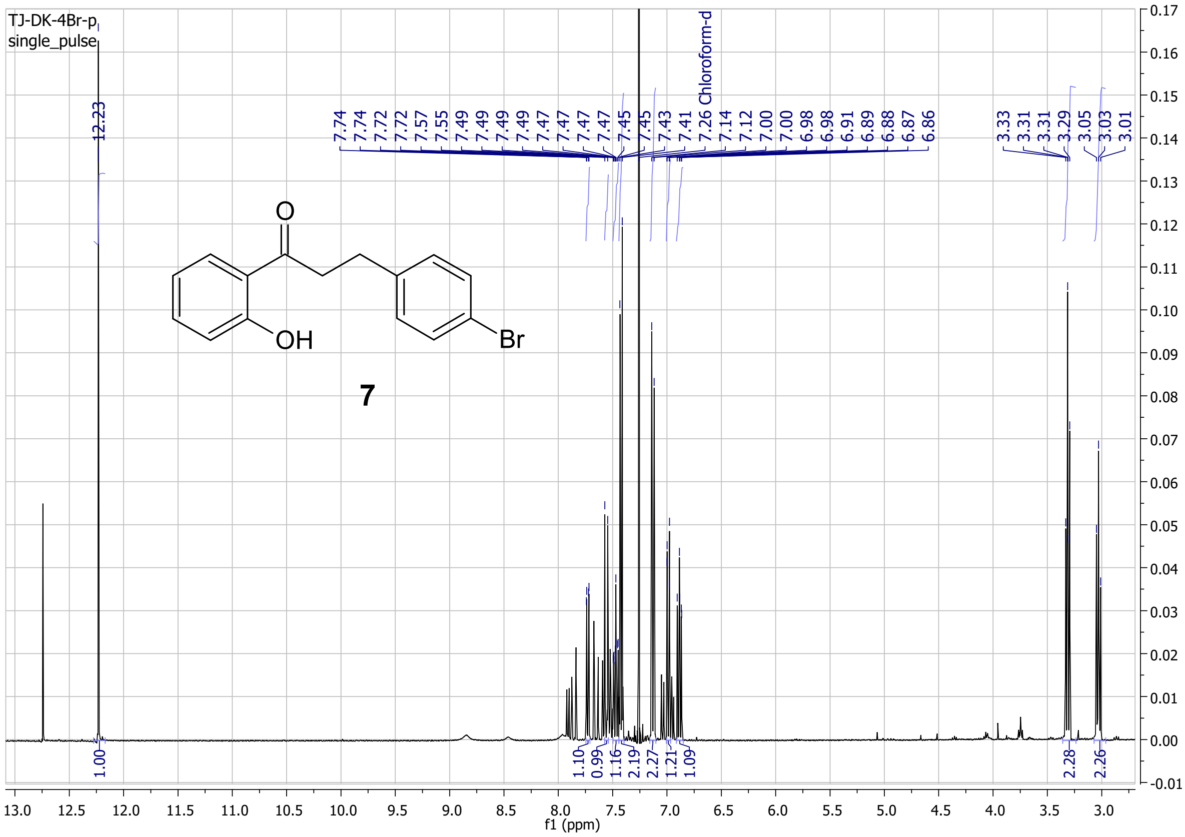

Figure S31. Part of the ^{13}C NMR spectral of 1-(2’-hydroxyphenyl)-3-(4”-bromophenyl)-propan-1-one (**7**) ($CDCl_3$, 600 MHz)

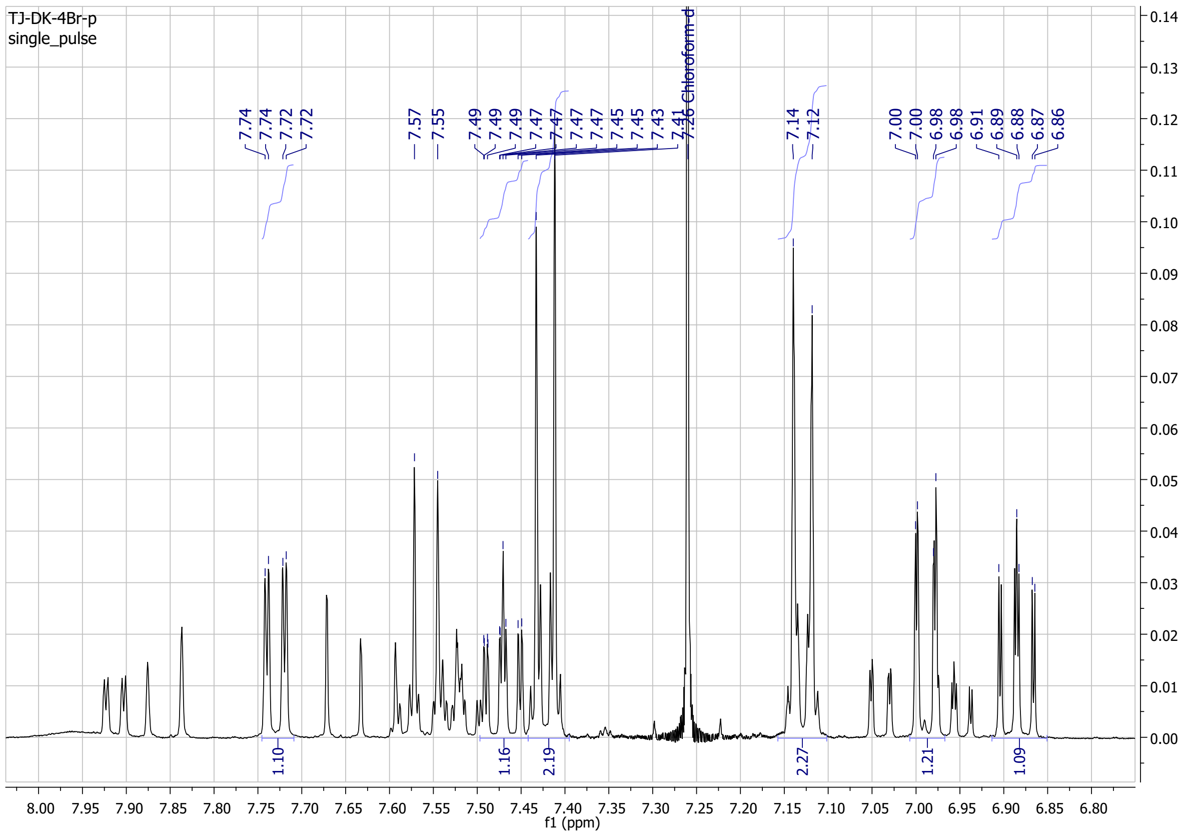

Figure S32. ^{13}C NMR spectral of 1-(2'-hydroxyphenyl)-3-(4"-bromophenyl)-propan-1-one (**7**) ($CDCl_3$, 151 MHz)

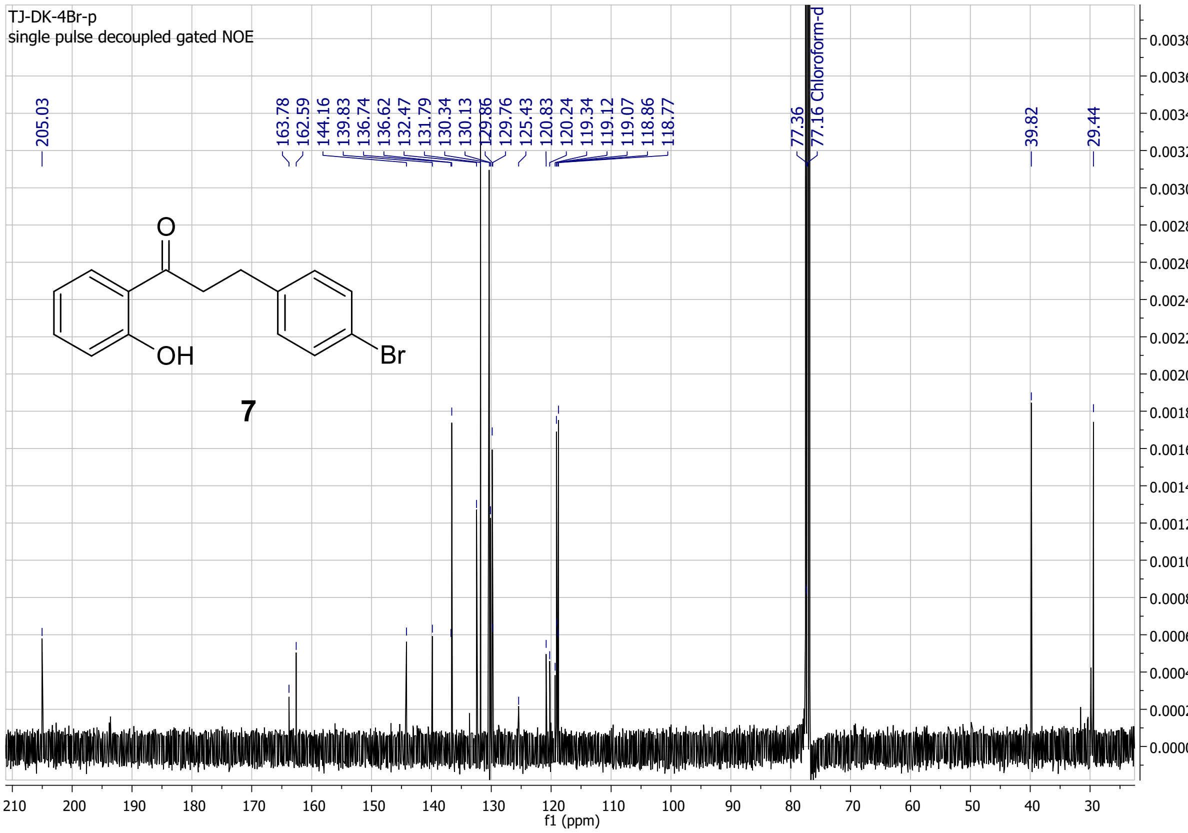

Figure S33. COSY spectral of 1-(2'-hydroxyphenyl)-3-(4"-bromophenyl)-propan-1-one (**7**) ($CDCl_3$, 151 MHz)

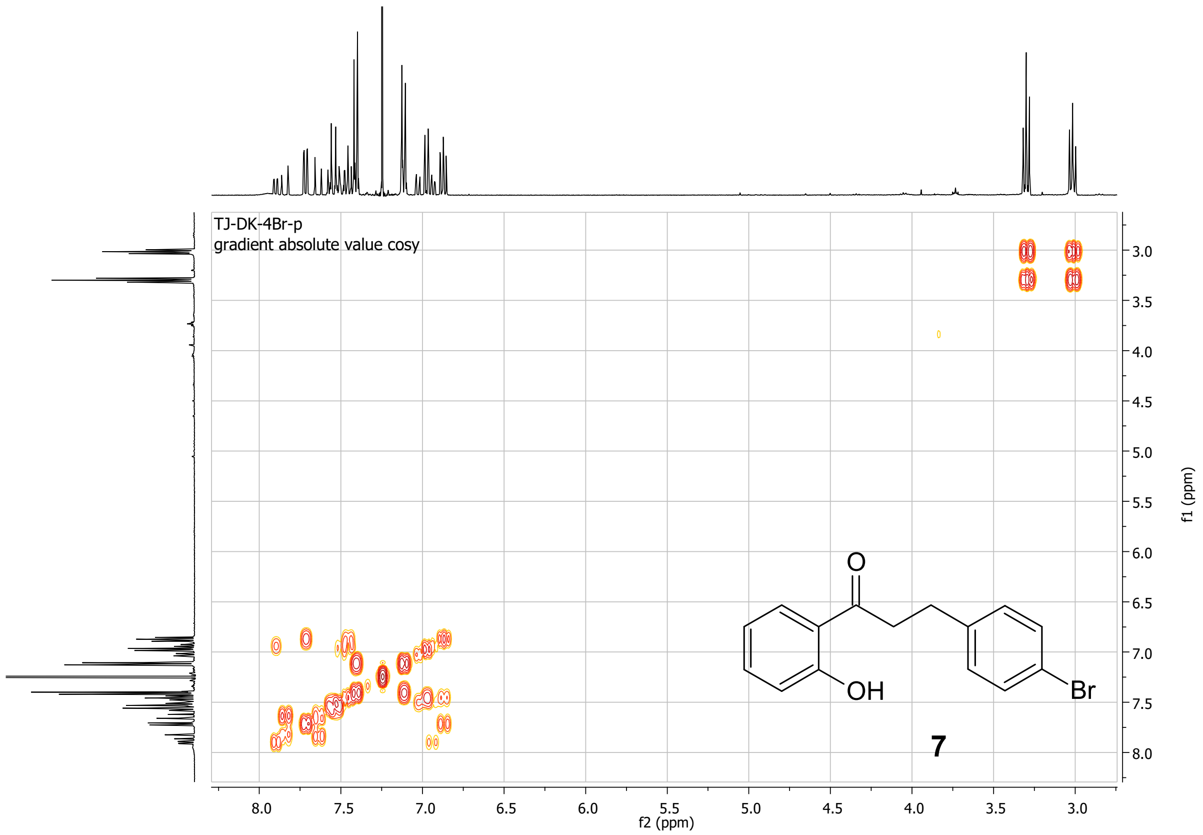

Figure S34. HSQC spectral of 1-(2'-hydroxyphenyl)-3-(4"-bromophenyl)-propan-1-one (**7**) ($CDCl_3$, 151 MHz)

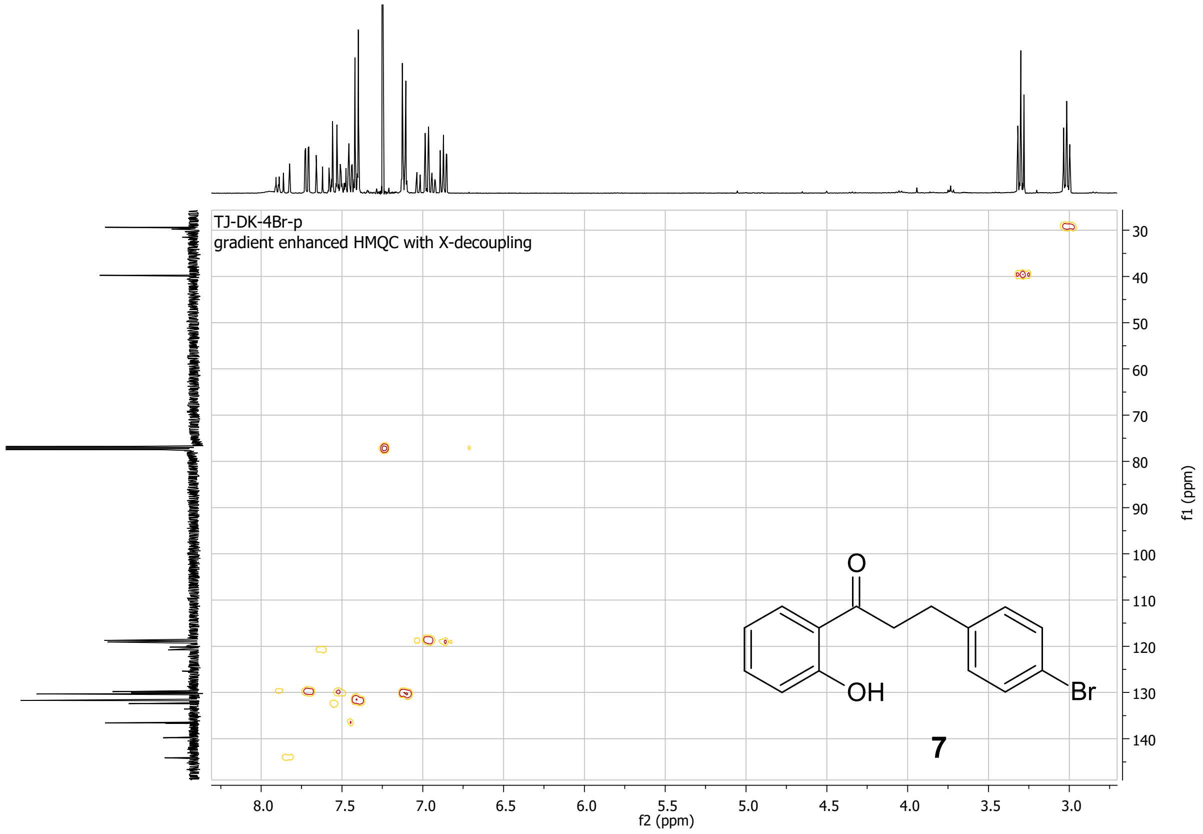

Figure S35. HMBC spectral of 1-(2'-hydroxyphenyl)-3-(4"-bromophenyl)-propan-1-one (**7**) ($CDCl_3$, 151 MHz)

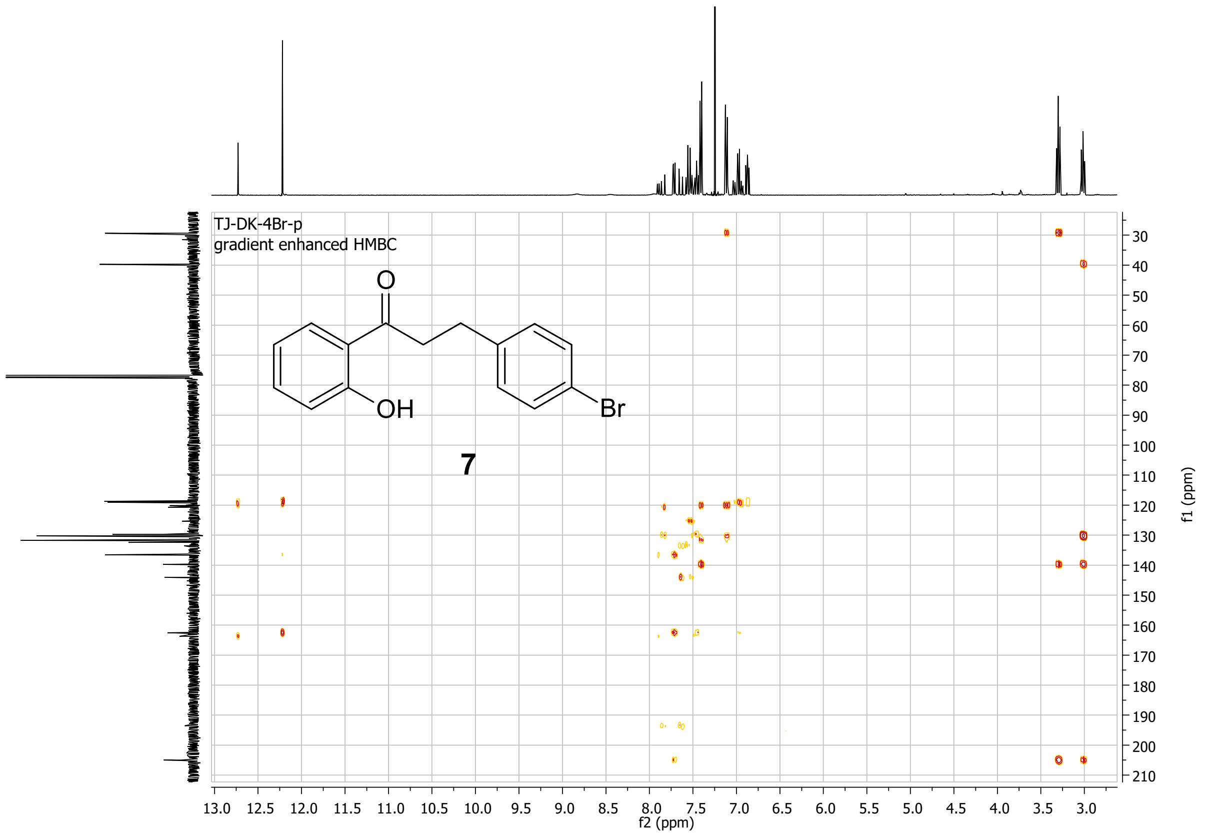

Figure S36. ^{1}H NMR spectral of 1-(5’-bromo-2’-hydroxyphenyl)-3-phenyl-propan-1-one (**4**) ($CDCl_3$, 600 MHz)

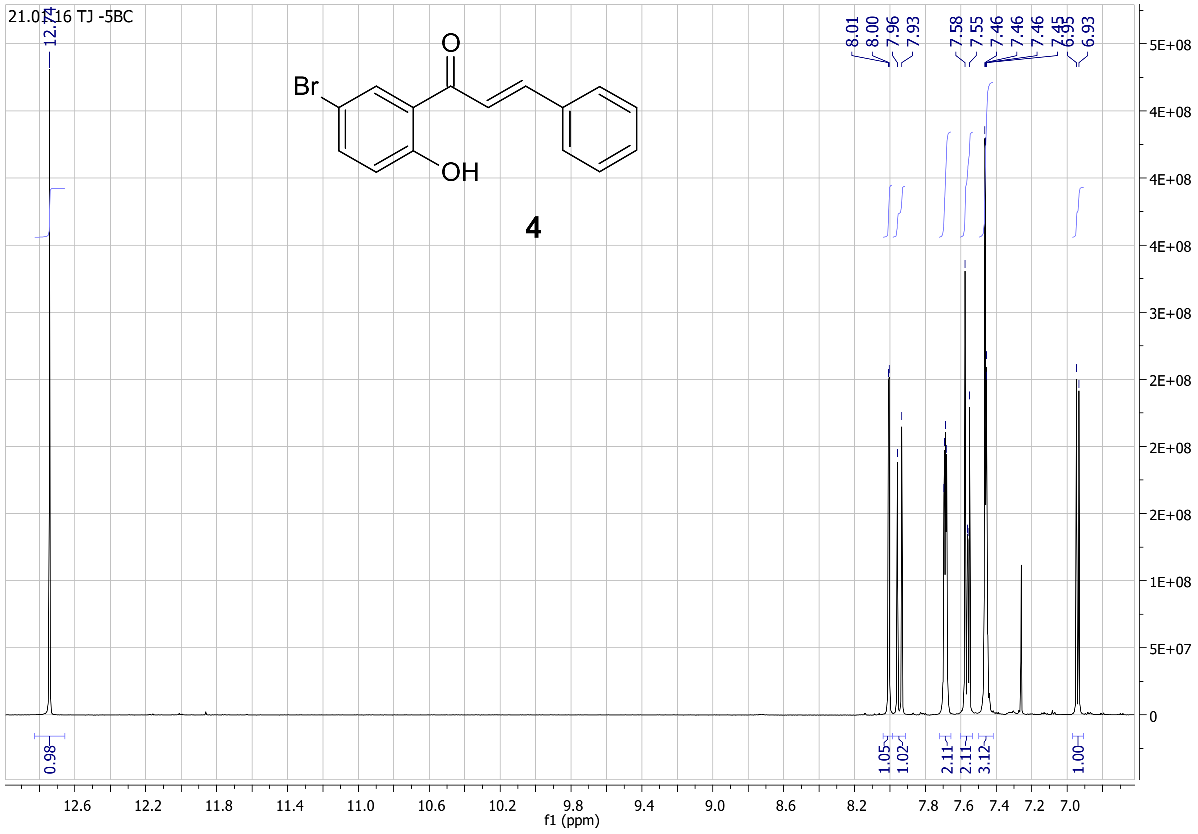

Figure S37. Part of the ^{1}H NMR spectral of 1-(5’-bromo-2’-hydroxyphenyl)-3-phenyl-propan-1-one (**4**)

($CDCl_3$, 600 MHz)

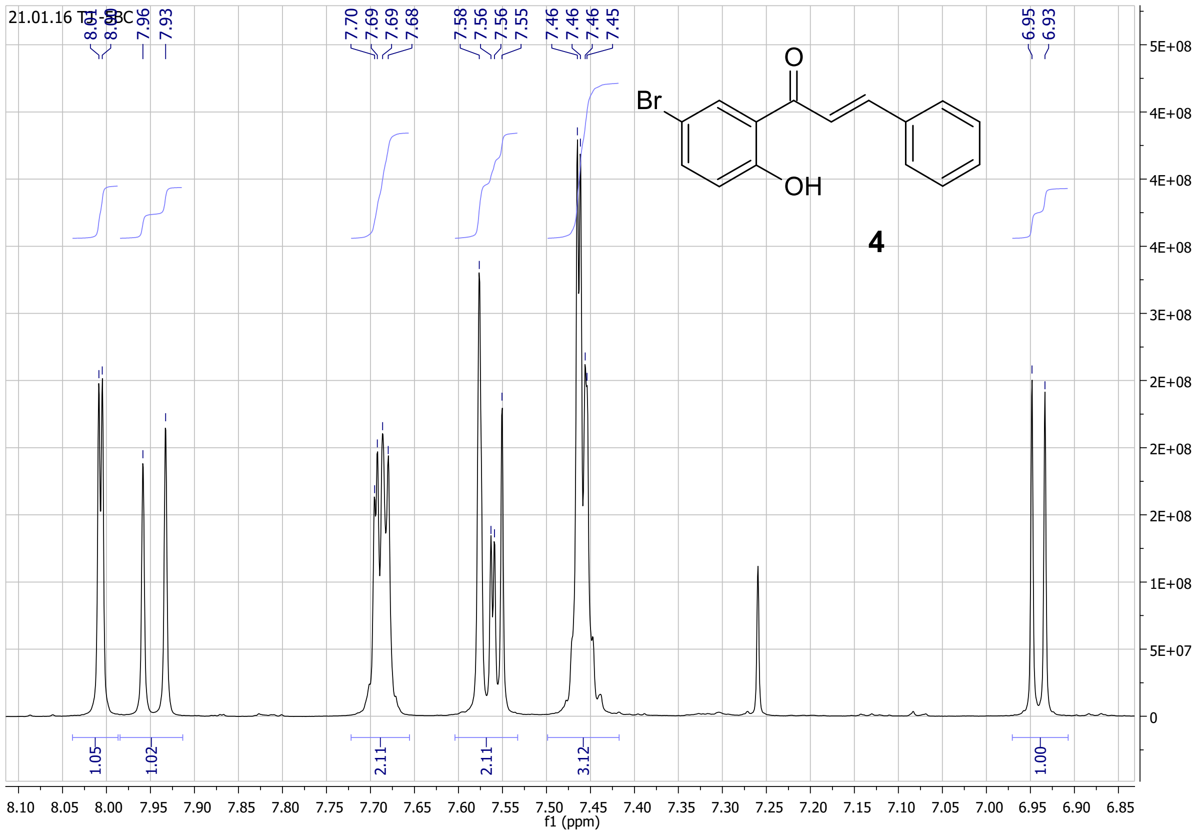

Figure S38. ^{13}C NMR spectral of 1-(5'-bromo-2'-hydroxyphenyl)-3-phenyl-propan-1-one (**4**) ($CDCl_3$, 151 MHz)

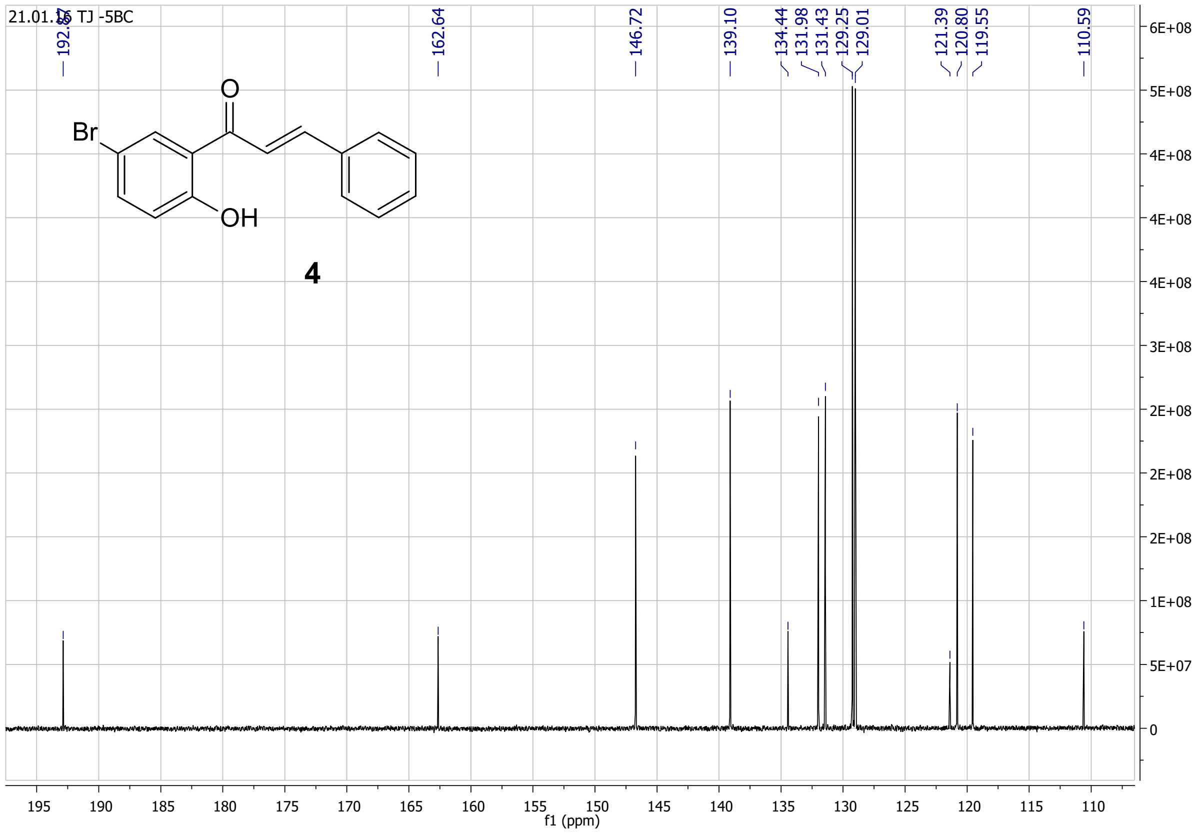

Figure S39. COSY spectral of 1-(5'-bromo-2'-hydroxyphenyl)-3-phenyl-propan-1-one (**4**) ($CDCl_3$, 151 MHz)

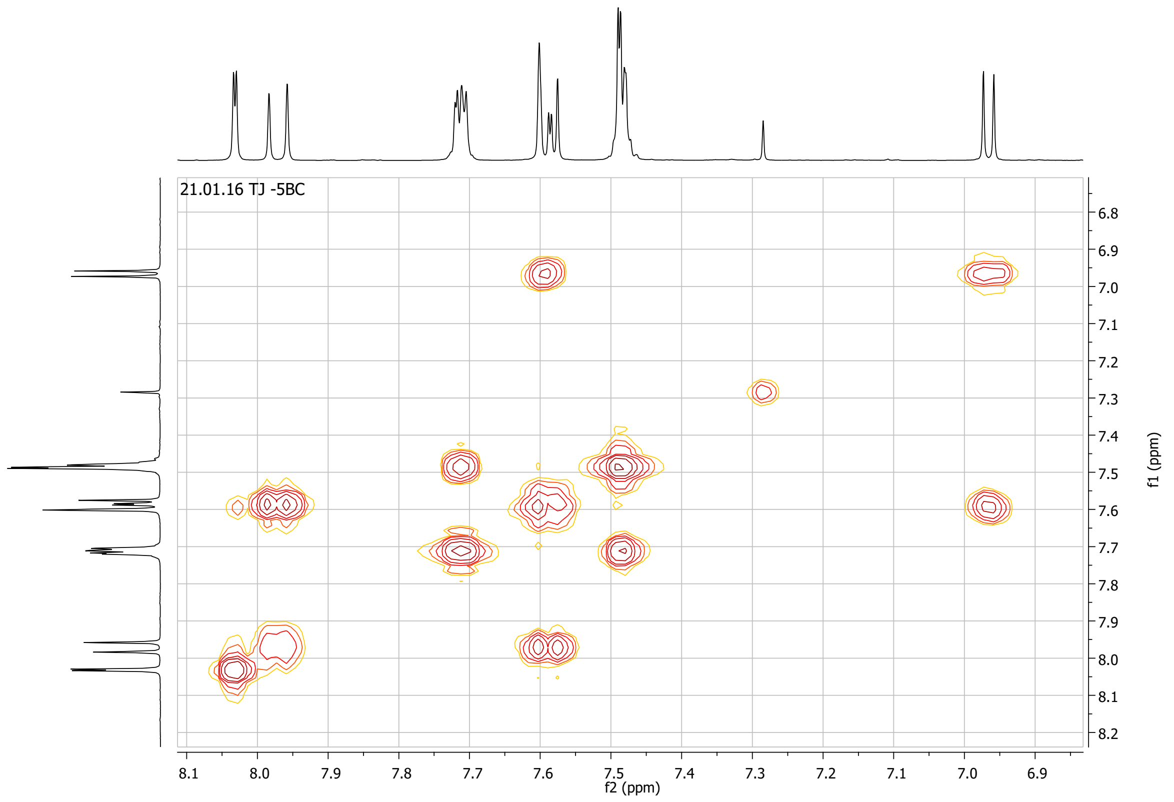

Figure S40. HSQC spectral of 1-(5'-bromo-2'-hydroxyphenyl)-3-phenyl-propan-1-one (**4**) ($CDCl_3$, 151 MHz)

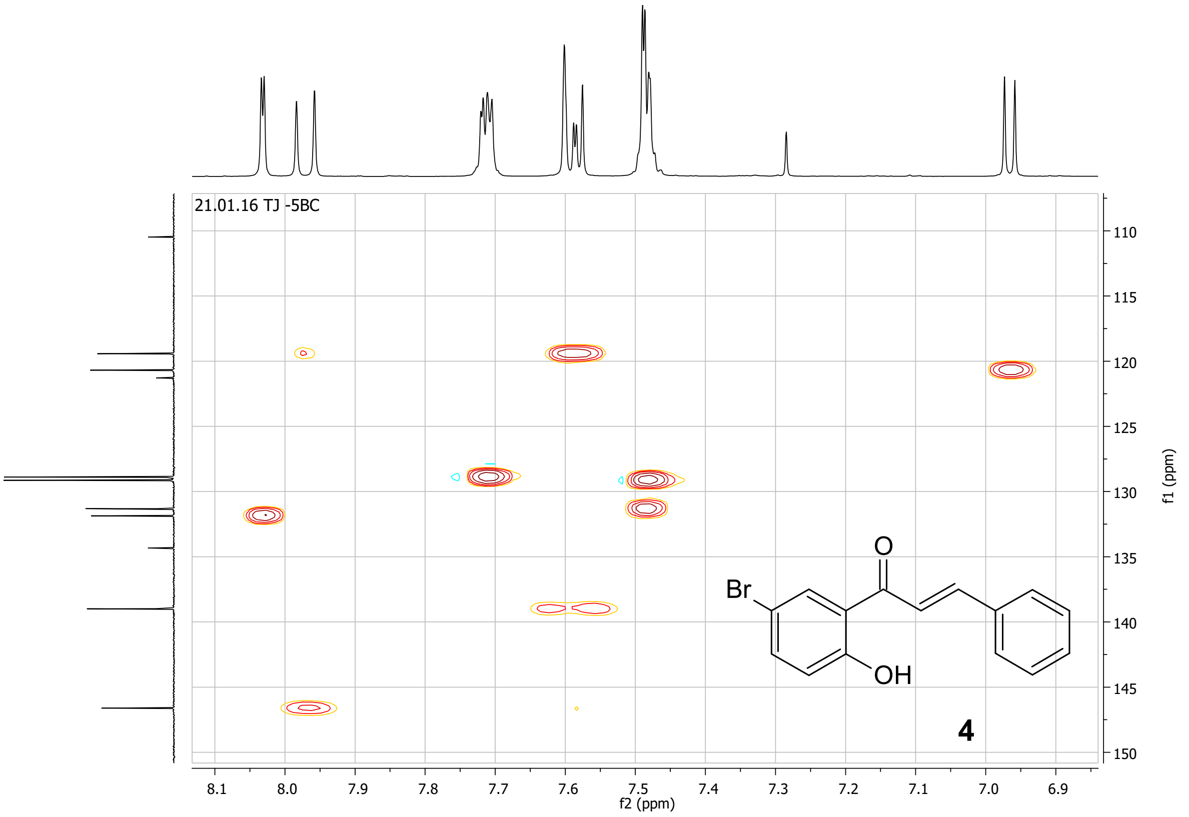

Figure S41. ^{1}H NMR spectral of 1-(5'-bromo-2'-hydroxyphenyl)-3-phenyl-prop-2-en-1-one (**8**) ($CDCl_3$, 600 MHz)

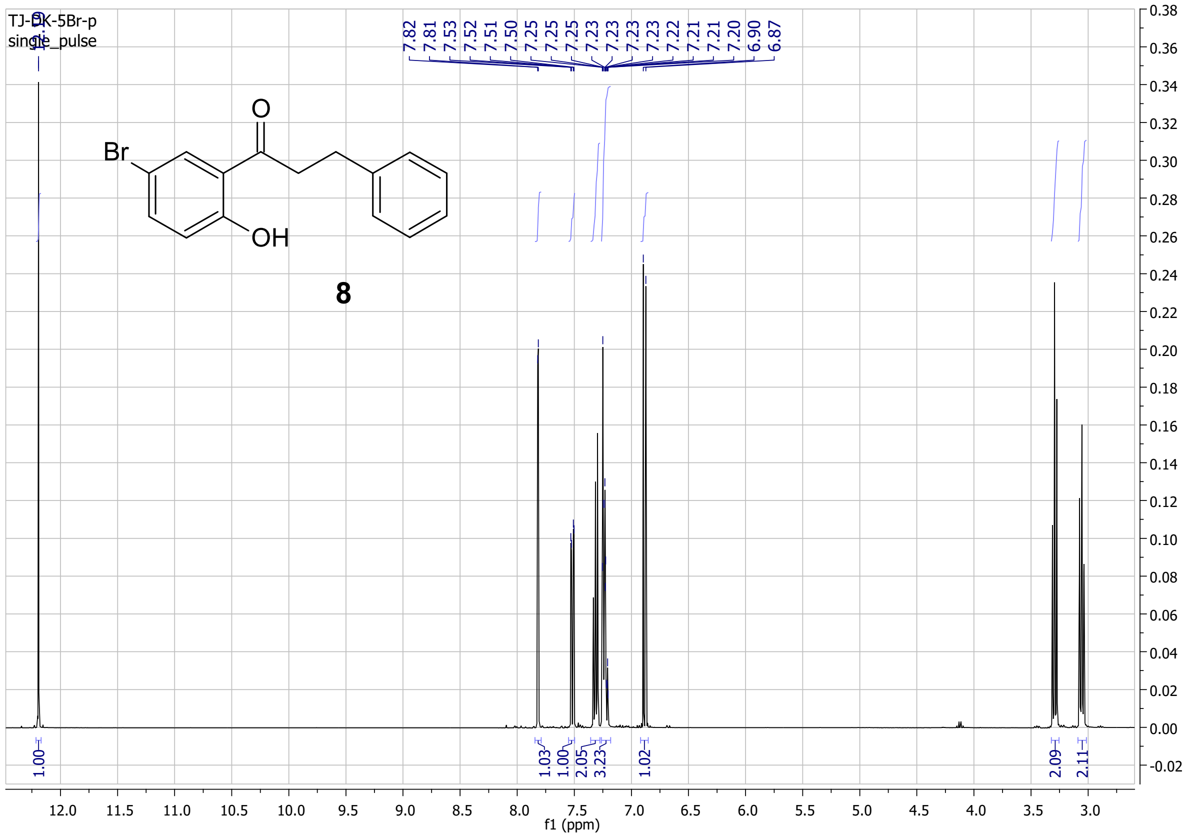

Figure S42. Part of the ^{13}C NMR spectral of 1-(5’-bromo-2’-hydroxyphenyl)-3-phenyl-prop-2-en-1-one (**8**) ($CDCl_3$, 600 MHz)

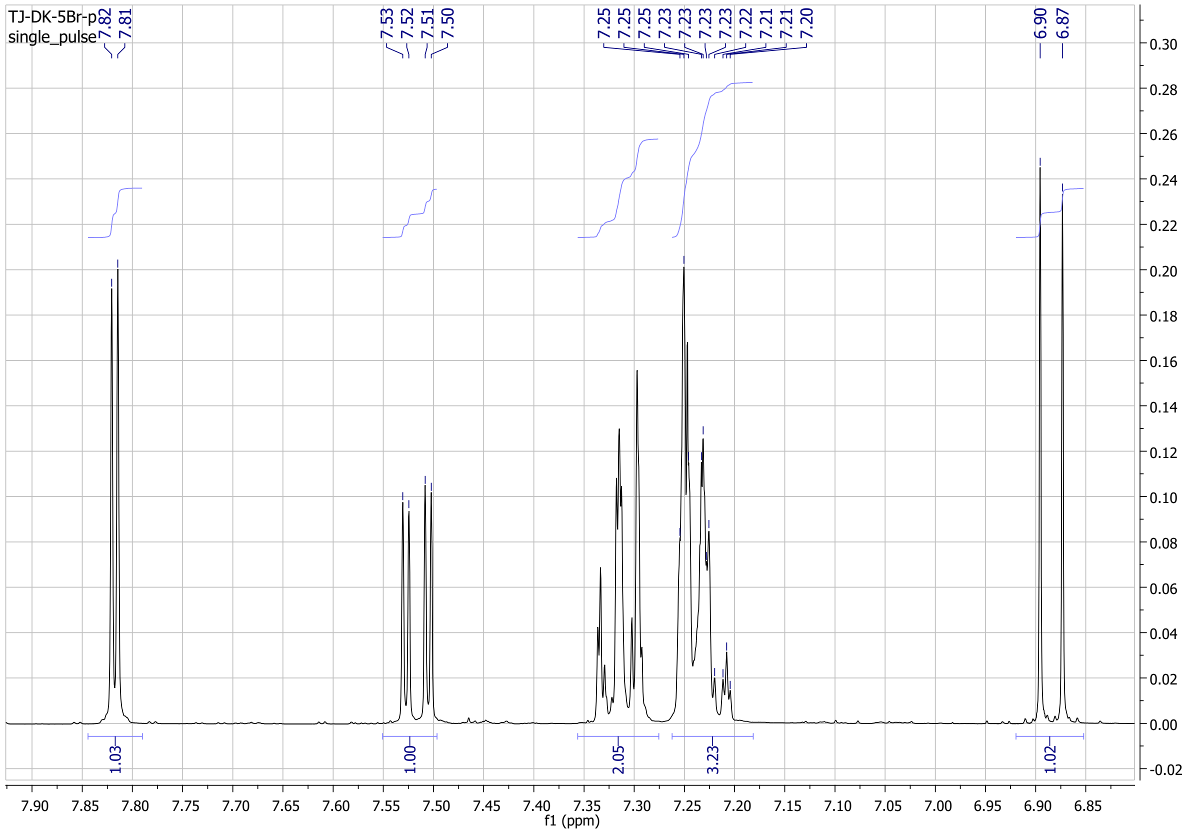

Figure S43. ^{13}C NMR spectral of 1-(5’-bromo-2’-hydroxyphenyl)-3-phenyl-prop-2-en-1-one (**8**) ($CDCl_3$, 151 MHz)

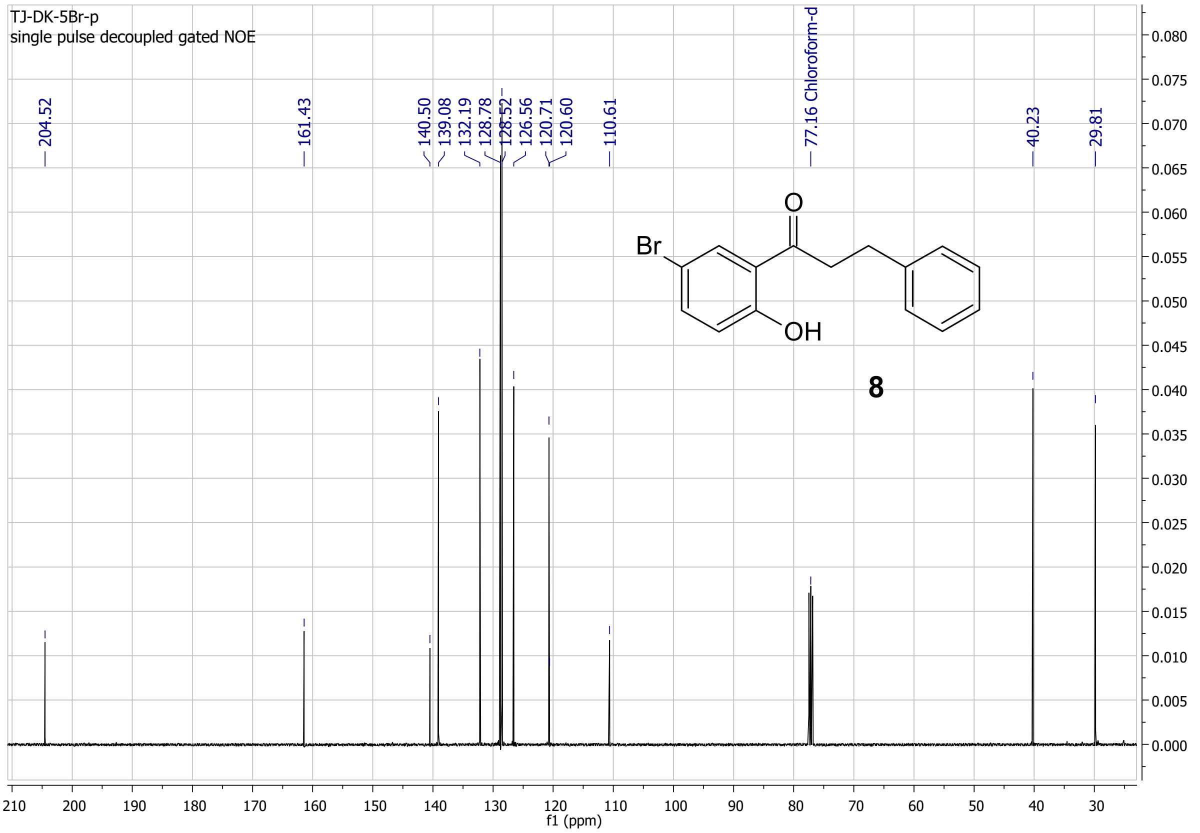

Figure S44. COSY spectral of 1-(5’-bromo-2’-hydroxyphenyl)-3-phenyl-prop-2-en-1-one (**8**) ($CDCl_3$, 151 MHz)

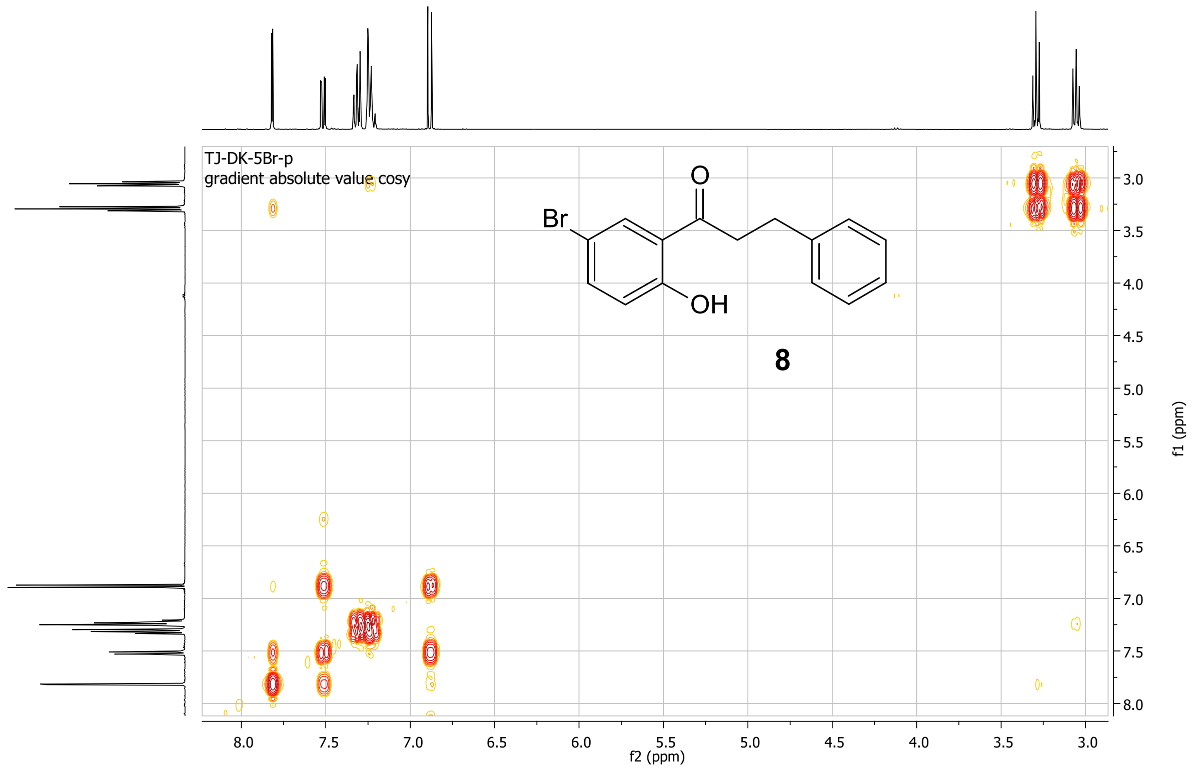

Figure S45. HSQC spectral of 1-(5'-bromo-2'-hydroxyphenyl)-3-phenyl-prop-2-en-1-one (**8**) ($CDCl_3$, 151 MHz)

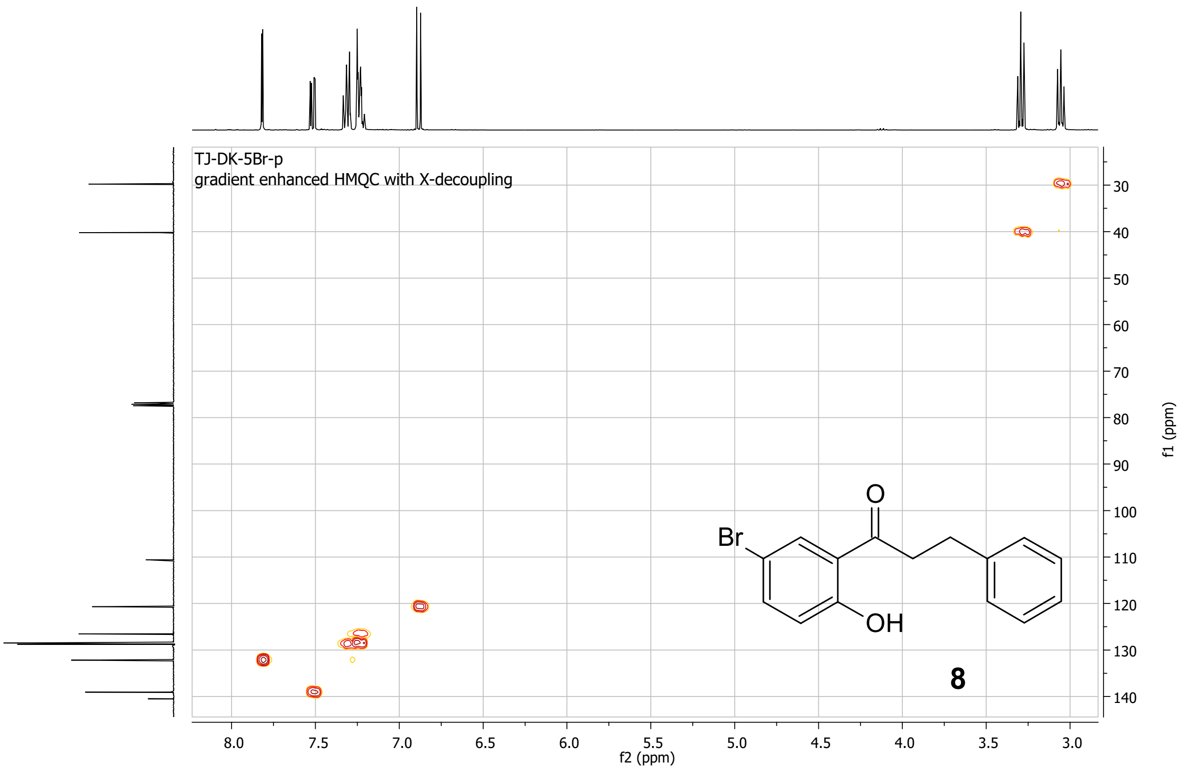

Figure S46. HMBC spectral of 1-(5'-bromo-2'-hydroxyphenyl)-3-phenyl-prop-2-en-1-one (**8**) ($CDCl_3$, 151 MHz)

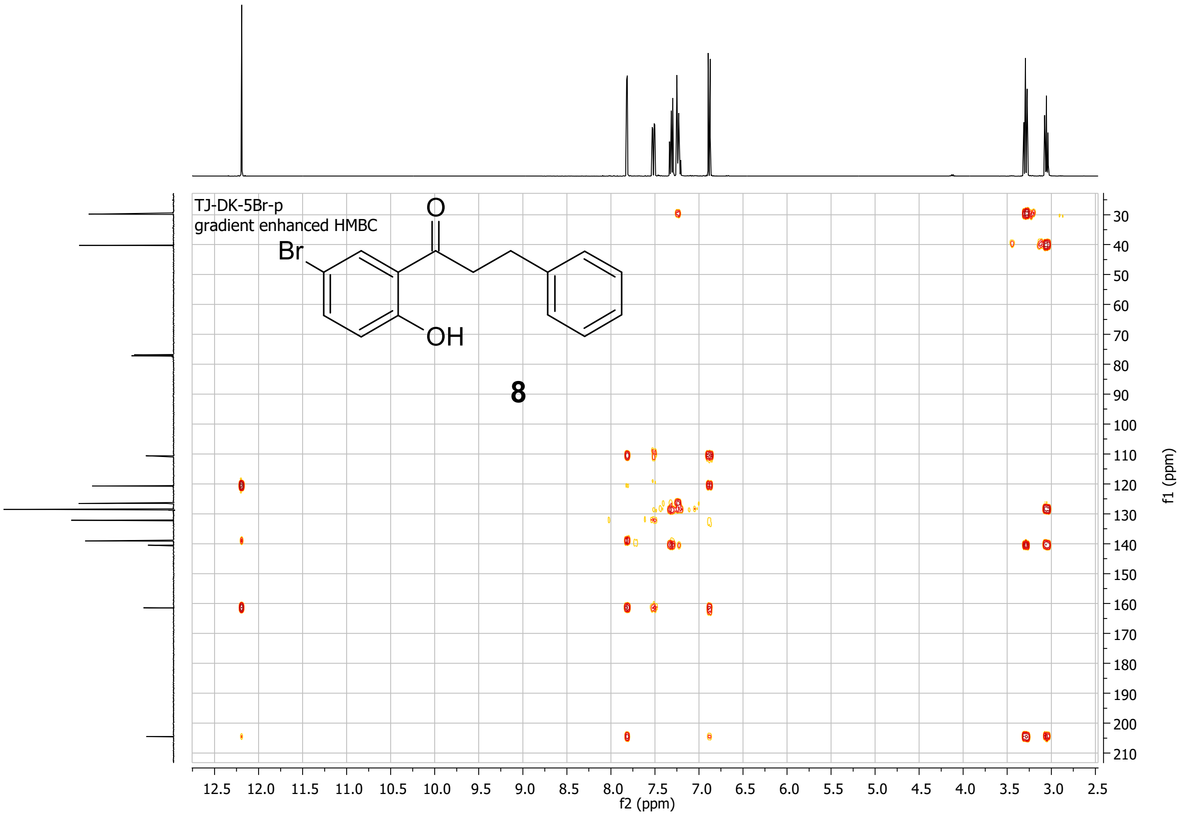

Figure S47. Preparative TLC plate showing the separation of the post-reaction mixture after biotransformation of 2'-hydroxy-2"-bromochalcone (**1**)

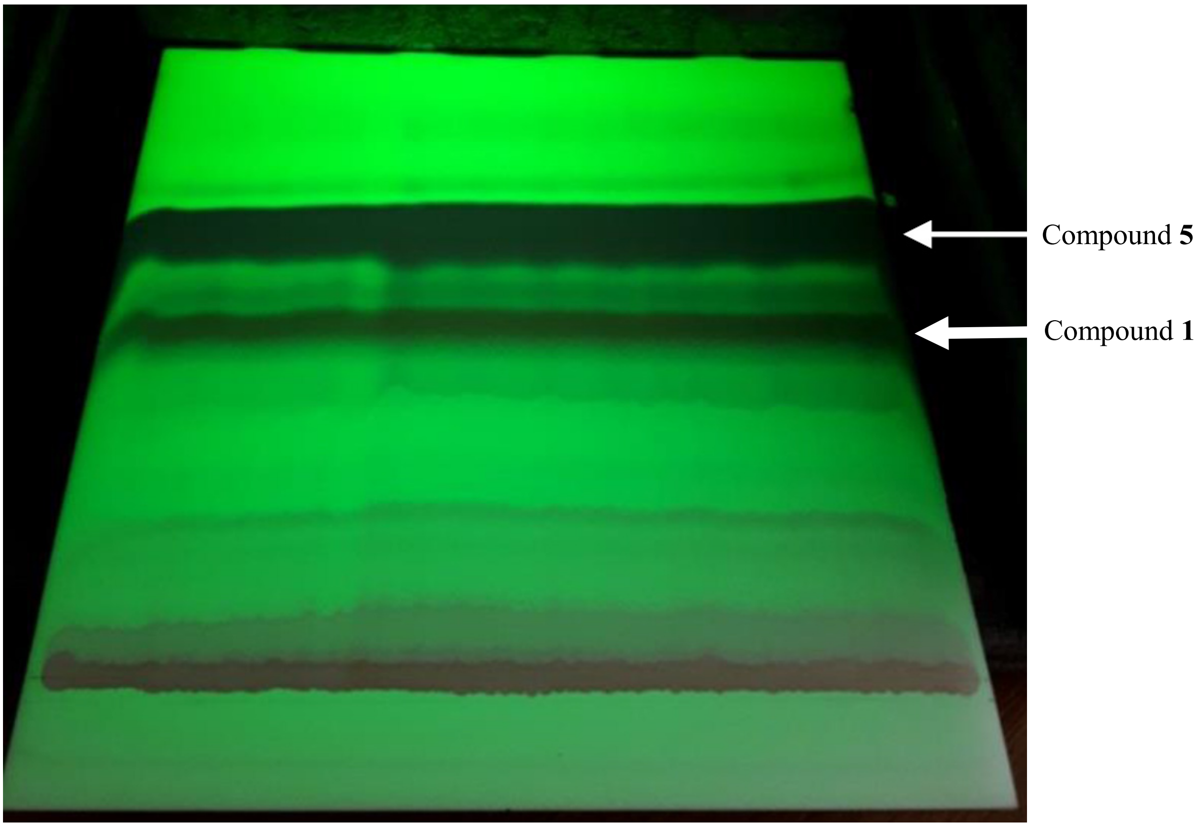

Figure S48. Preparative TLC plate showing the separation of the post-reaction mixture after biotransformation of 2'-hydroxy-3"-bromochalcone (**2**)

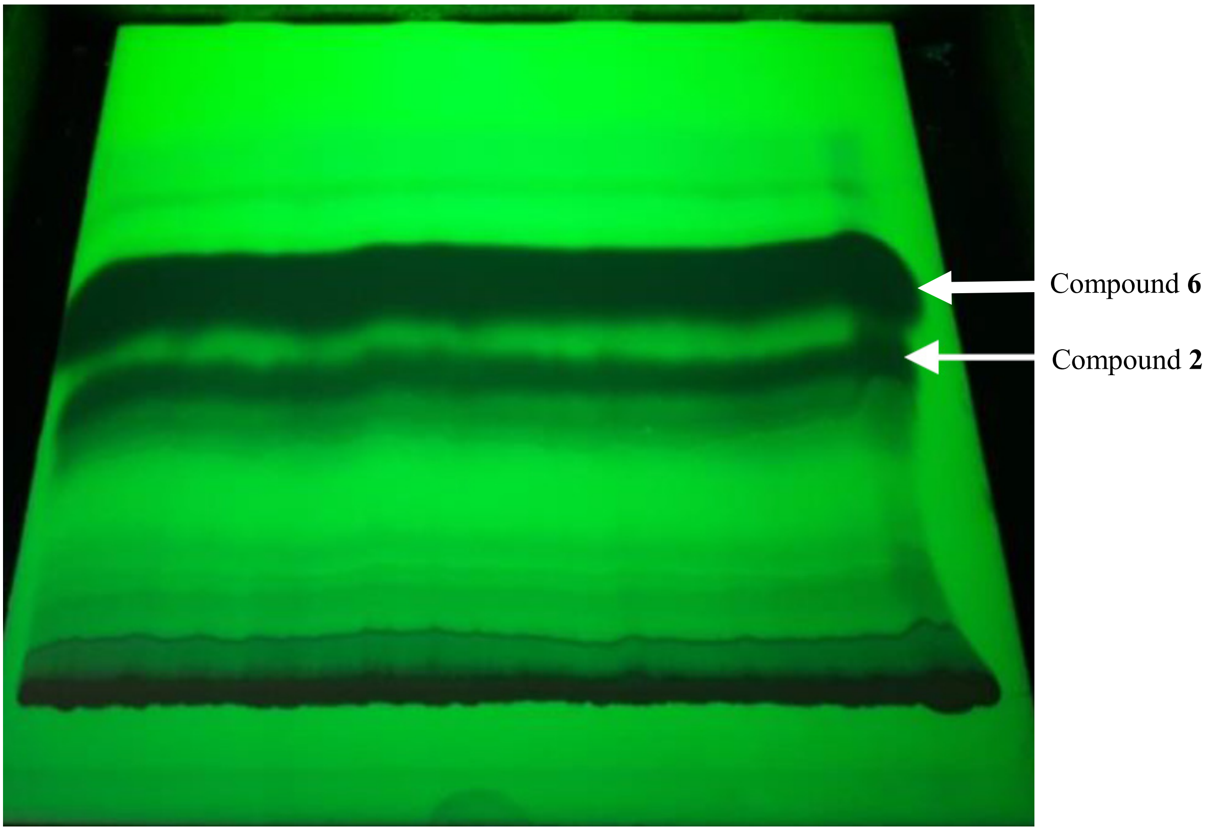

Figure S49. Preparative TLC plate showing the separation of the post-reaction mixture after biotransformation of 2'-hydroxy-4''-bromochalcone (**3**)

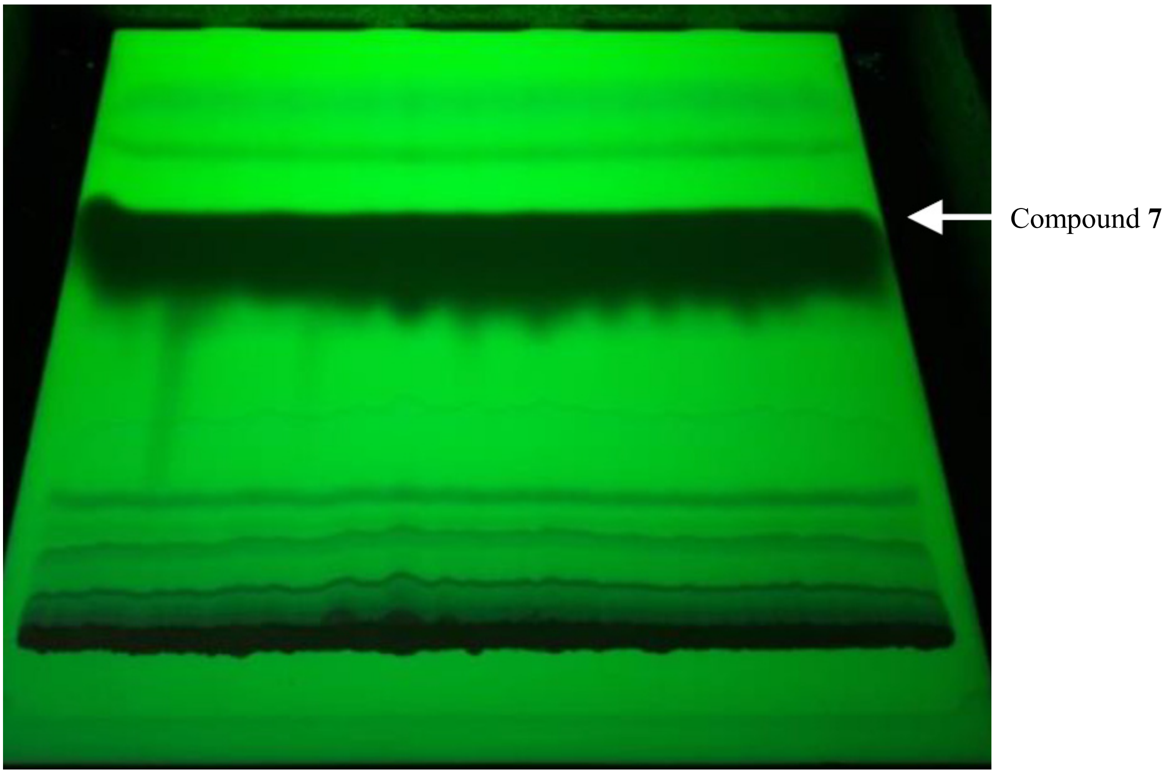

Figure S50. Preparative TLC plate showing the separation of the post-reaction mixture after biotransformation of 2'-hydroxy-5'-bromochalcone (**4**)

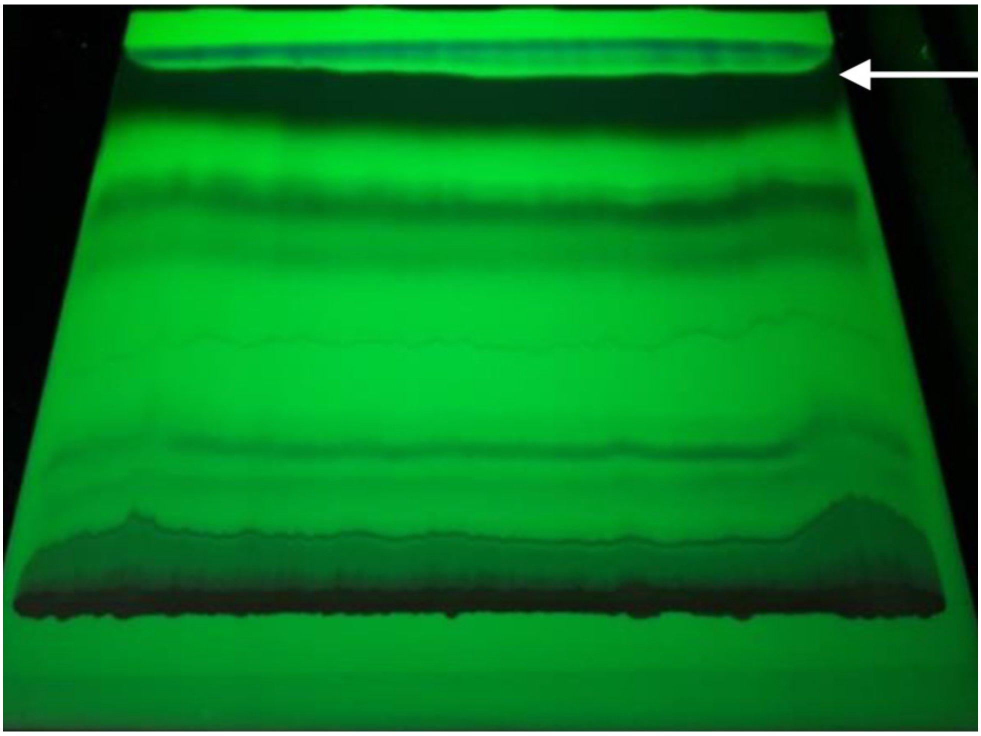

Compound **8**

Figure S51. Selected chromatograms showing the composition of the reaction mixtures during the biotransformation of the tested compounds:

a) GC analysis obtained after 12 hours transformation of 2'-hydroxy-2"-bromochalcone (**1**)

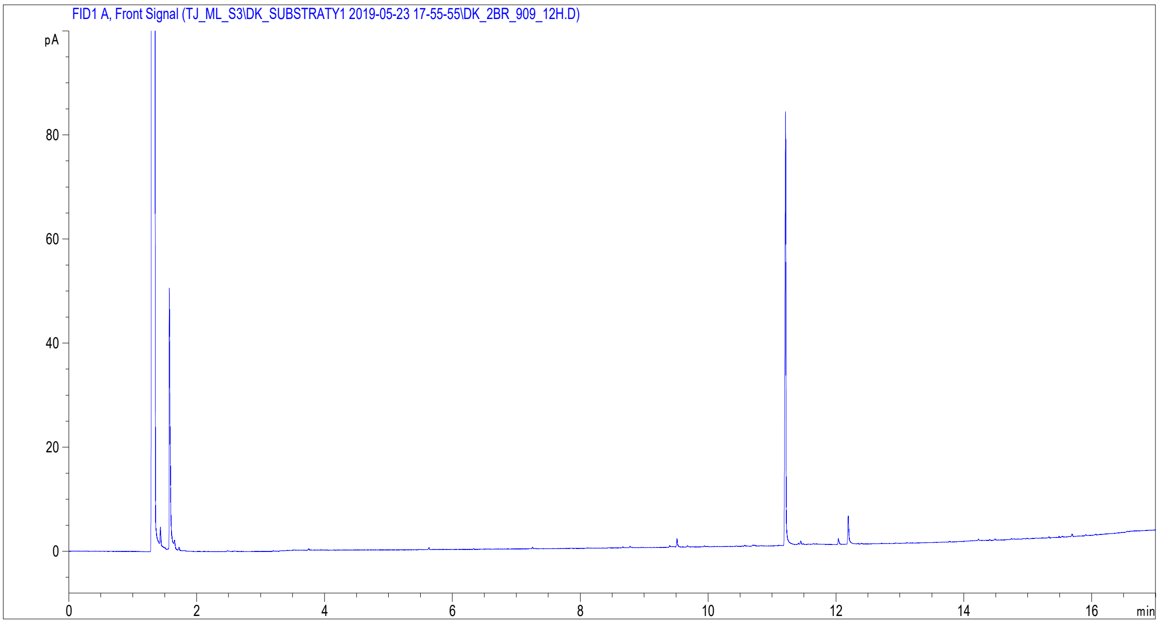

b) GC analysis obtained after 3 days transformation of 2'-hydroxy-2"-bromochalcone (**2**)

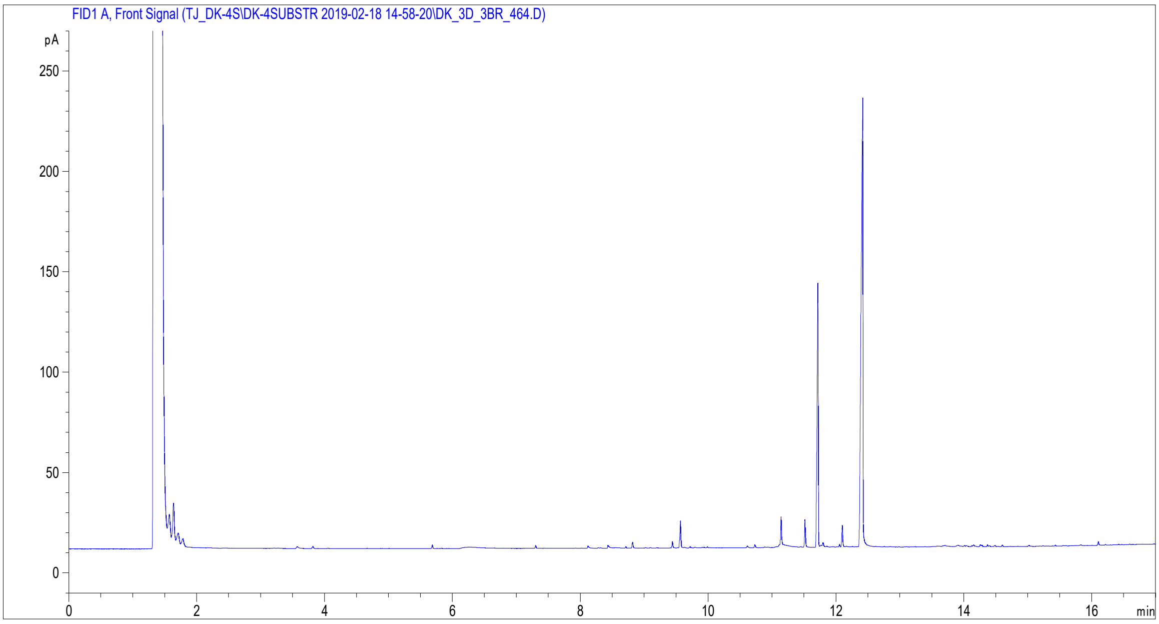

c) GC analysis obtained after 3 hours transformation of 2'-hydroxy-5'-bromochalcone (**4**)

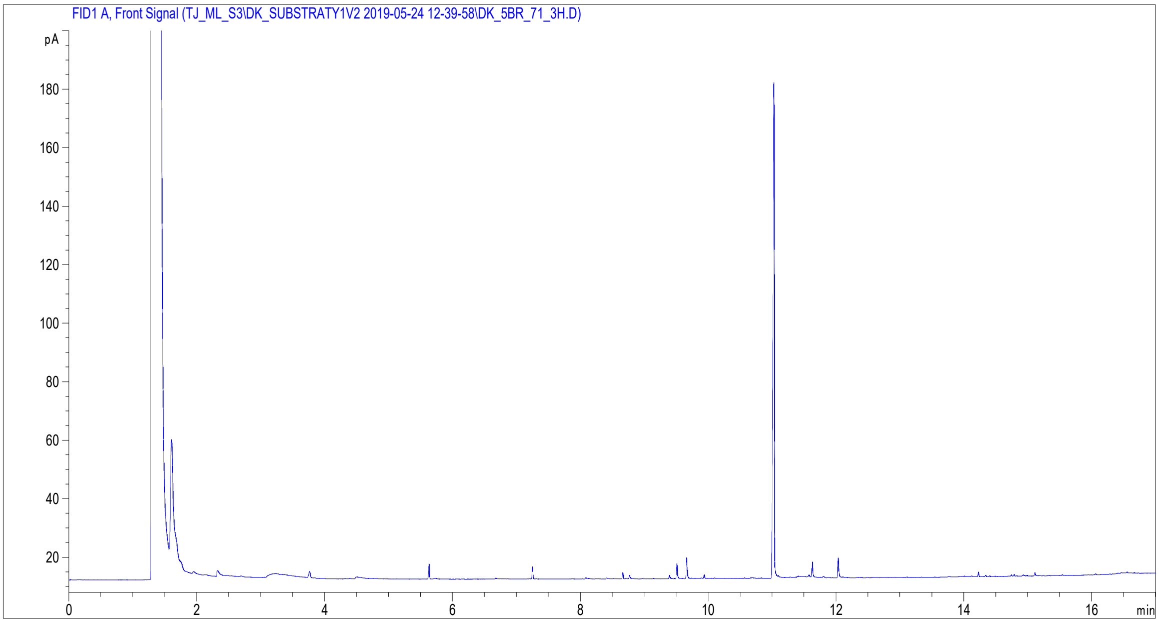

d) GC analysis obtained after 10 days transformation of 2'-hydroxy-5'-bromochalcone (**4**)

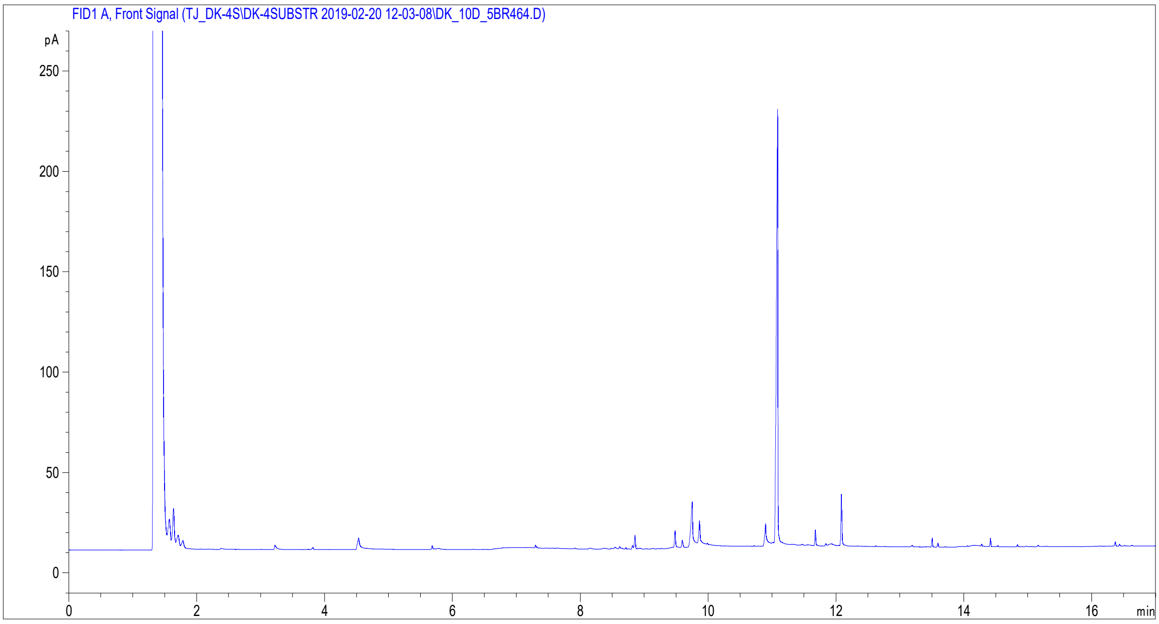
FID1 A, Front Signal (TJ_DK-4S\DK-4SUBSTR 2019-02-20 12-03-08\DK_10D_5BR464.D)
pA
250
200
150
100
50
0
0
2
4
6
8
10
12
14
16
min
